# Everything changes but nothing changes: gender stereotypes in the Italian population

**DOI:** 10.1007/s00737-024-01437-1

**Published:** 2024-02-06

**Authors:** Rosana Carvalho Silva, Marika Vezzoli, Valentina Menesello, Mattia Meattini, Riccardo Sartori, Alessandra Minelli

**Affiliations:** 1 Department of Molecular and Translational Medicine, University of Brescia, Brescia, Italy; 2 Genetics Unit, IRCCS Istituto Centro San Giovanni Di Dio Fatebenefratelli, Brescia, Italy; 3 Department of Human Sciences, University of Verona, Verona, Italy

**Keywords:** Gender stereotypes, Online survey, Gender role, Gender equality, Internalized beliefs

## Abstract

**Purpose:**

Gender stereotypes refer to consensual or cultural shared beliefs about the attributes of men and women, influencing society behaviors, interpersonal relationships, education, and workplace. The literature has shown the existence of gender stereotypes on career choices, internalization of roles, and school and social experiences and demonstrates the impact of demographic factors on stereotypes. However, all the studies conducted in Italy available in scientific literature analyzed small sample sizes within specific schools of university settings, with a limited age range.

**Methods:**

To assess the current state of gender stereotypes in Italy, we conducted an online survey from October 2022 to January 2023 on the general population residing in Italy. The questionnaire comprised sociodemographic factors and questions about gender stereotypes, investigating six fields: games, jobs, personality traits, home and family activities, sports, and moral judgments.

**Results:**

The study involved 1854 participants, mostly women (70.1%) with an undergraduate or postgraduate degree (57.5%). The statistical and descriptive analyses revealed that gender stereotypes influenced respondents’ beliefs, with statistically significant effects observed in most questions when stratifying by age, gender, and degree. Principal component analysis was performed to assess latent variables in different fields, revealing significant main stereotypes in each category. No statistically significant differences between men and women were found for the fields home and family activities, games, and moral judgments, confirming that stereotypes affect both men and women in the same way.

**Conclusions:**

Our results show the persistence of gender stereotypes in any fields investigated, although our cohort is predominantly composed of high educational level women living in the North of Italy. This demonstrates that the long-standing gender stereotypes are prevalent, pernicious, and, unfortunately, internalized at times even by successful women pushbacking and sabotaging them unconsciously.

**Supplementary Information:**

The online version contains supplementary material available at 10.1007/s00737-024-01437-1.

## Introduction

Gender stereotypes refer to consensual or cultural shared beliefs about the attributes of men and women, from sex-based categorization and biological differences to social and cultural group representations (Eagly et al. 2020; Ellemers 2018). Constructs like communality and agency are used in gender stereotype studies, with communality related to affectionate traits, orienting people toward others, being more prevalent in females, and agency related to goal-achievement, including assertiveness and competitiveness, prevailing in males (Sczesny et al. 2018).

These stereotypes influence societal behaviors, impacting relationships, education, and the workplace, with physical and psychological effects. Stereotypes associate high-level intellectual abilities with men, discouraging women from pursuing certain careers, and these stereotypes are endorsed by young children, shaping their interests (Bian et al. 2017). This can be observed in the disparity between men and women in science, technology, engineering, and mathematics (STEM), in which women are largely underrepresented (Botella et al. 2019; Reinking & Martin 2018). Women are also underrepresented in occupations requiring competitiveness and physical resistance and overrepresented in jobs requiring empathy and communality. There is still a disparity in housework and childcare, often due to internalized beliefs (Cortes & Pan 2018; Hentschel et al. 2019).

Few studies have been performed about gender stereotypes using representative samples. A meta-analysis conducted in the USA integrating 16 public opinion polls on gender stereotypes, extending over 70 years and including about 30,000 adults, showed that affectionate and emotional characteristics were more related to women, with this relation increasing over time. Features such as ambition and courage were more associated with men, without changes over time. Although women gained in competence perception relative to men, gender equality inferences on this domain, including intelligence and creativity, increased with time (Eagly et al. 2020). Another study compared gender stereotype data collected in the USA in 1980 to data collected in 2014 to analyze if stereotypes have changed with more women participating in workforce, athletics, and education. Results showed that stereotypes remained stable, except for an increase in stereotyping of female roles (Haines et al. 2016).

Few gender stereotype studies were performed in Italy. Ramaci and collaborators explored the influence of gender stereotypes on adolescents’ career choices through self-report questionnaires assessed in 120 Italian high school students, highlighting the impact of age, gender, and parents’ profession on vocational decision-making (Ramaci et al. 2017). Professional and social identity diverged as individuals grew older and males presented higher perceived occupational self-efficacy in military, scientific-technological, and agrarian occupations compared to females (Ramaci et al. 2017). Siyanova-Chanturia and colleagues investigated real-time processing of gender stereotypes, investigating 28 university students, 30 older adults, and 85 children aged 8–11 in the province of Modena, Italy. The participants were required to make quick judgments on whether two auditorily presented words, one representing a masculine or feminine stereotype and the other a male or female kinship term, could both be used to describe the same individual. Participants responded faster when the target gender was congruent with the stereotypical gender use of the preceding prime, pointing to gender asymmetric perceptions and mental representations of words (Siyanova-Chanturia et al. 2015). Cerbara and collaborators conducted a survey through a structured questionnaire in schools of Rome, Italy, on 412 children aged 8–11 and examined the adherence to gender roles among children, emphasizing the internalization of traditional gender roles and its association with age and prosociality (Cerbara et al. 2022). Results showed the internalization of traditional gender roles, with boys accepting more male roles and girls accepting more female roles, with acceptance decreasing as age increases, suggesting that gender stereotypes are internalized at a young age, being influenced by primary socialization (Cerbara et al. 2022). Finally, Musso and collaborators studied 213 Italian adolescents in Apulia and 214 Nigerian adolescents in Enugu State, in secondary school, measuring school empowerment and engagement, STEM-gender stereotypes, and socioeconomic status (Musso et al. 2022). Nigerian girls and boys had higher levels of school empowerment and engagement, and STEM-gender stereotypes compared to Italian peers, and boys scored higher on school empowerment and STEM-gender stereotypes. Higher school empowerment was related to lower STEM-gender stereotypes in both Italian and Nigerian girls’ groups, while higher school engagement was associated with lower STEM-gender stereotypes only in Nigerian groups.

In summary, the national studies investigated different aspects of gender stereotypes and their effects on specific populations. Despite the differences in methodologies and designs, all studies highlighted the impact of gender stereotypes on diverse aspects of individuals’ lives, including career choices, language processing, and adherence to traditional gender roles. The studies focused on small groups in particular settings, involving individuals with a narrow age range, and examined restricted aspects of gender stereotypes, emphasizing the need for broader investigations. To address this gap, we conducted an online survey among individuals residing in Italy, with the objective of depicting the current state of gender stereotypes.

## Materials and methods

### Study design

We conducted an online survey to perform an exploratory research analysis using a Google Form from the beginning of October 2022 to the end of January 2023. Eligible participants were aged 15 or older, residing in Italy. To ensure a diverse audience, the survey link was widely spread, distributed among university colleagues, health professionals, and students in different regions. Social networks maximized the reach of the survey. We used a snowball sampling procedure by asking participants to disseminate the survey link among contacts. No Ethics formal approval was needed for this survey. To participate, individuals had to answer a yes–no question confirming their voluntary willingness to participate in the survey. Once confirmed, participants were directed to anonymously complete a self-report questionnaire.

### Measures

The questionnaire consisted of two sections: sociodemographic factors and gender stereotypes. The online survey took about 10 min to complete and was written in Italian. The sociodemographic variables included gender, age, education, field of study, occupation, marital status, region of residence, and geographical origin of one’s family. Since there are no validated scales available on this topic, we created an ad hoc questionnaire to assess gender stereotypes, comprising 56 questions developed based on a literature review. The items investigated six fields (games, profession/jobs, personality traits, home and family activities, sports, and moral judgments) randomly displayed in the questionnaire. Three items were reversed questions inserted to control the reliability of each subject’s answers. Gender stereotypes were assessed using a Likert scale, including 1 (“completely agree”), 2 (“strongly agree”), 3 (“somewhat agree”), 4 (“slightly disagree”), 5 (“strongly disagree”), and 6 (“no difference between men and women”).

### Statistical analysis

The chi-square test was applied to analyze the variables. Since the questionnaire contained six latent constructs, for each of them, we applied principal component analysis (PCA) (Jolliffe & Cadima 2016) to extract a small number of latent variables, easily interpret, and able to summarize these concepts not directly measurable. To identify the optimal number of components for each subgroup of items, the proportion of variance explained was observed, considering the Principal Components (PCs) that, in cumulative terms, explained at least 70% of the total variance. Loadings were extracted from the analysis output to show the contribution of each item on the component itself. Loadings could be positive or negative, indicating the direction and strength of the relationship between items and the corresponding PC. Larger absolute values suggested a stronger influence of one (or more) item on the PC and a significant contribution to its definition. Conversely, items with low or near-zero coefficients on the loadings had little impact on the component.

## Results

A total of 1880 participants completed the questionnaire. Two expert psychologist reviewers independently checked for incongruent answers in the control questions, excluding subjects who answered unreliably and 26 respondents were excluded. The final sample consisted of 1854 subjects, with 70.1% being women and 57.5% possessing at least a university degree. Detailed socio-demographic characteristics are shown in Table 1.

**Table 1:** Socio-demographic characteristics of the participants included in the survey

	Overall (*N* = 1844)
**Gender**	
Female	1292 (70.1%)
Male	534 (29.0%)
Other/do not want to answer	18 (1.0%)
**Age**	
15–18	74 (4.0%)
19–22	223 (12.1%)
23–30	347 (18.8%)
31–40	336 (18.2%)
41–50	304 (16.5%)
51–60	296 (16.1%)
61–70	214 (11.6%)
Over 70	50 (2.7%)
**Degree**	
Elementary and secondary school degrees	122 (6.6%)
High school degree	662 (35.9%)
University degree	737 (40.0%)
Post-University degrees	323 (17.5%)
**Job**	
Student	418 (22.7%)
Housewife	36 (2.0%)
Retired	166 (9.0%)
Unemployed	33 (1.8%)
Freelancer professionals	265 (14.4%)
Permanent worker	654 (35.5%)
Fixed-term worker	186 (10.1%)
Other	86 (4.7%)
**Marital status**	
Unmarried	827 (44.8%)
Married	861 (46.7%)
Divorced	132 (7.2%)
Widowed	24 (1.3%)
**Family origins**	
Italian descendent	1769 (95.9%)
Non-Italian descendent	75 (4.1%)
**Italian area**	
N-Miss	15
North	1568 (85.7%)
Center	90 (4.9%)
South	171 (9.3%)

Descriptive results presenting the overall percentage of agreement among respondents are shown in Table 2. The total percentage of agreement was computed by summing the percentages of responses “completely agree,” “strongly agree,” and “somewhat agree.” Sentences with the highest agreement percentages in the total cohort, accounting for more than 25% each, were as follows: “Rugby is a sport for males,” “Women are more sensitive than men,” “Women are braver than men,” “Boxing is a sport for males,” “Working as a babysitter is for women,” and “The military profession is for men.”

**Table 2:** Descriptive results showing the total percentage of agreement (in descending order) among all survey questionnaire respondents. The original Italian questions were translated into English

Questions (latent construct)	Total % of agreement*
Rugby is a sport for males (sports)	35.80%
Women are more sensitive than men (personality traits)	33.50%
Women are braver than men (personality traits)	30.20%
Boxing is a sport for males (sports)	29.00%
Working as a babysitter is for women (profession)	28.00%
The military profession is for men (profession)	26.50%
Men are more aggressive than women (personality traits)	23.60%
Women are more determined than men (personality traits)	23.40%
Playing with dolls is for girls (games)	22.80%
Women are cunning than men (personality traits)	21.40%
Men drive better than women (home and family activities)	20.70%
For men, more than for women, it is very important to have success in their job (personality traits)	20.00%
Artistic gymnastics is for females (sports)	19.00%
Women are more stubborn than men (personality traits)	17.60%
Working in a butcher shop is for men (profession)	17.00%
Women are more competitive than men (personality traits)	16.30%
Women are kinder than men (personality traits)	15.70%
Football is a sport for males (sports)	13.80%
Ironing is an activity for women (home and family activities)	13.60%
Videogames are for males (games)	12.60%
Women are more jealous than men (personality traits)	12.10%
Women are more generous than men (personality traits)	9.80%
It is women, more than men, who must take care of the children (home and family activities)	9.60%
Cycling is a sport for males (sports)	8.60%
Being a pilot is a job for men (profession)	8.30%
It is men, more than women, who have to support the family (home and family activities)	7.50%
Nursing profession is for women (profession)	6.90%
Women are more aggressive than men (personality traits)	6.80%
Being a bus driver is a job for women (profession)	5.40%
At home, it is men, more than women, who command (home and family activities)	4.90%
Karate is a sport for males (sports)	4.90%
Women are nicer than men (personality traits)	4.70%
Women are more selfish than men (personality traits)	4.70%
Men are more inclined towards scientific subjects than women (profession)	4.70%
It is women, more than men, who have to cook at home (home and family activities)	4.70%
The surgical profession is a job for men (profession)	4.50%
It is men, more than women, who are responsible for investing the family's savings (home and family activities)	4.10%
The bricks are a game for boys (games)	4.00%
The profession of psychotherapist is for women (profession)	3.90%
The toy cars are a game for girls (games)	3.50%
The surgical profession is a job for women (profession)	3.50%
It is understandable that a man, more than a woman, can lose patience (moral judgments)	3.50%
It is men, more than women, who have to make the most important decisions concerning the family (home and family activities)	3.30%
Women are more disorganized than men (personality traits)	3.20%
Rugby is a sport for females (sports)	2.90%
Sewing is an activity for men (home and family activities)	2.50%
In a couple, female infidelity is more serious than male infidelity (moral judgments)	2.50%
It is men, more than women, who have to clean the house (home and family activities)	2.20%
It is men, more than women, who have to help the children with their homework (home and family activities)	1.90%
Basketball is a game for females (sports)	1.80%
The tricycle is a game for boys (games)	1.70%
Stuffed animals are a toy for boys (games)	1.70%
A woman who works will never be completely a good mother and partner (moral judgments)	1.60%
Tennis is a sport for males (sports)	1.50%
Skiing is a sport for males (sports)	1.00%
Puzzles are a game for males (games)	0.40%

^*^The total percentage of agreement was calculated by summing the percentages of responses “completely agree,” “strongly agree,” and “somewhat agree.” This value is reported only for descriptive purposes and was not considered for the statistical analysis

We conducted stratified analyses by age, gender, and degree since all categories for these variables were numerically well-represented. For most items, we found highly significant effects, confirming that these variables strongly influence gender stereotypes. We observed significant effects in 54 questions when stratifying by age, 49 questions when stratifying by gender, and 51 when stratifying by degree, out of 56 questions. Detailed results are available in Supplementary Table 1Sa to 6Sc.

PCA was conducted to evaluate latent variables from the corresponding fields (Supplementary Table 7S). This was possible due to a preliminary analysis demonstrating a strong positive correlation among all individual items (correlation *p*-values < 0.05) (data available upon request).

For “home and family activities,” four latent variables lead to an explained variance ≥ 70%. The first component accounted for 47% of the variance and was primarily influenced by three items: “Ironing is an activity for women,” “Men drive better than women,” and “It is women, more than men, who must take care of the children.” When examining the field games,” three latent variables achieved an explained variance greater than 70%. The first component explained 52% of the variance and was mainly influenced by the item “Playing with dolls is for girls.” For “moral judgments,” two latent variables were considered. The first component, responsible for 66% of the explained variance, was primarily influenced by two items: “It is understandable that a man, more than a woman, can lose patience” and “In a couple, female infidelity is more serious than male infidelity.” For these three fields, no significant differences were found when conducting separate analyses for women and men.

For “personality traits,” six latent variables lead to an explained variance ≥ 70%. The first component, explaining 40% of the variance, was influenced by three items: “Women are more sensitive than men,” “Women are more determined than men,” and “Women are more cunning than men.” However, among women, two items had a greater influence (“Women are braver than men” and “Women are more determined than men”), while among men, two different items were more influential (“Women are more cunning than men” and “Women are more sensitive than men”).

For “profession/jobs,” four latent variables were considered and the first component, accounting for 48% of the explained variance, was influenced by two items: “The military profession is for men” and “Working as a babysitter is for women.” Among men, an additional item (“Working in a butcher shop is for men”) also had an impact on the first component, explaining 52% of the variance.

Finally, when examining “sports,” three latent variables explained a variance ≥ 70%. The first component, explaining 59% of the variance, was influenced by two items: “Boxing is a sport for males” and “Rugby is a sport for males.” Among males, in addition to the aforementioned items, two other components contributed to the variance (57%): “Artistic gymnastics is for females” and “Football is a sport for males.”

**Fig. 1:**
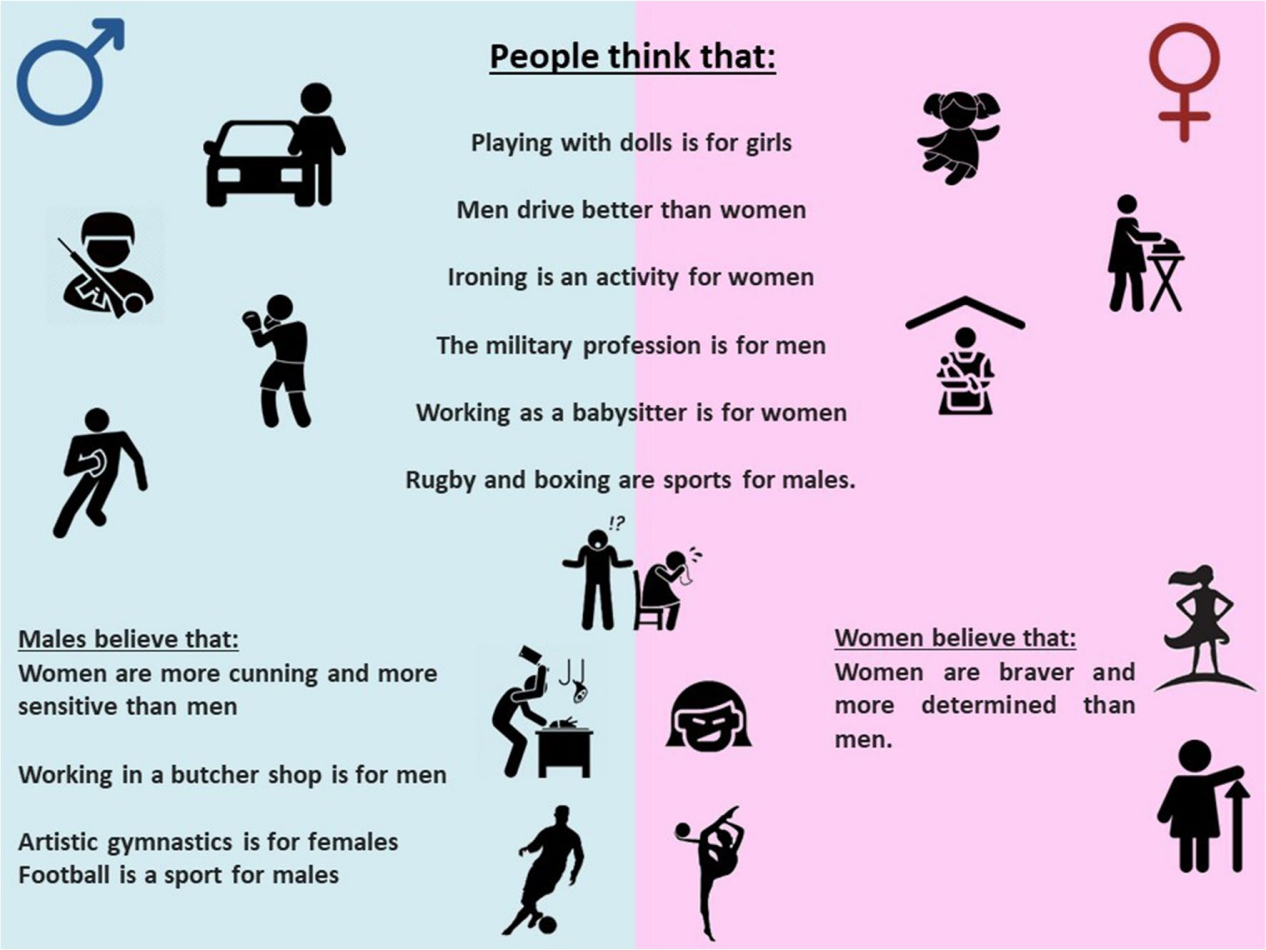
Main outcome results coming from the different analyses performed indicating the items more relevant in terms of stereotypes

The main results coming from the different analyses performed indicating the most relevant items in terms of stereotypes are summarized in Fig. 1.

## Discussion

We conducted an online survey to perform an exploratory research analysis in 1854 participants residing in Italy to depict the state of gender stereotypes. Gender stereotypes strongly influenced respondents’ beliefs, with statistically significant effects observed in most questions when stratifying by age, gender, and education, showing the influence of demographic factors. Indeed, highly significant effects were observed for the majority of items, specifically for 96% of the questions when stratifying by age, 91% of the questions when stratifying by degree, and 87% of the questions when stratifying by gender. These results provide compelling evidence regarding the influence of gender stereotypes on individual’s beliefs across different demographic groups and their ability to shape perceptions and beliefs in the studied population. PCA was performed to assess latent variables in different fields, revealing substantial main stereotypes in each category. For “home and family activities,” “games,” and “moral judgments,” the results did not reveal a statistically significant difference between men and women, demonstrating that stereotypes had an equal impact on both genders.

Studies from the literature have examined the presence of gender stereotypes and their impacts on career aspirations, vocational decision-making, internalization of gender roles, and the representation of gender subgroups in different cultures. Unfortunately, as previously described, few studies have been performed in Italy (Cerbara et al. 2022; Musso et al. 2022; Ramaci et al. 2017; Siyanova-Chanturia et al. 2015). When comparing the findings of these studies with ours, some common themes can be identified, despite the different methodologies. The studies acknowledge the existence of gender stereotypes on career choices, internalization of roles, and school and social experiences and demonstrate the impact of demographic factors on gender stereotypes. However, the studies conducted in Italy available in scientific literature analyzed small sample sizes with a limited age range, within particular settings, such as schools or university environments. Our study adds a valuable contribution to the literature in the theme, by analyzing a broader sample, with a larger age range distributed in different national settings. Besides, we have analyzed various aspects of gender stereotypes by including questions investigating diverse fields, including games, profession/jobs, personality traits, home and family activities, sports, and moral judgments.

Some limitations must be addressed. In our cohort, there is a higher prevalence of women. It is known that gender stereotypes affect women more directly as they are often targeted by these stereotypes, probably enhancing their motivation to engage in discussions on these matters. When investigating the existence of stereotypes that tend to disadvantage one category over another, it is mainly representatives of the disadvantaged category who respond. Women are increasingly empowered to challenge stereotypes and address gender-related issues, making them more interested in speaking about and offering help with these topics. These factors may contribute to women being more inclined to respond to gender stereotypes surveys (Ellemers 2018; Hentschel et al. 2019).

Approximately 57% of the respondents achieved at least a 3-year university degree. Low occupational class and educational level have been associated with a low participation rate, despite the fact that determinants of non-participation have varied among research and communities (Reinikainen et al. 2018). Additionally, issues related to stereotypes and social prejudices are perceived differently by people with low or high educational attainment. People with low education are less sensitive to these issues and tend to act as if stereotypes describe reality as it must be, without reasoning about it (O’Brien et al. 2020; Shu et al. 2022). Conversely, people with higher education tend to be more sensitive to these issues (Reinikainen et al. 2018). If we also consider that, in Italy, women tend to have a higher level of schooling than men and that women tend to prefer university paths related to social sciences, this might explain why mostly women with high schooling participated in this survey.

Despite using the same distribution strategies, about 85% of respondents live in the North of Italy, indicating possible difficulties in addressing this issue in some areas. Italy is conventionally divided into North, Center, and South. In addition to geopolitical and economic characteristics, this division reflects cultural differences, also based on a lesser or greater acceptance of gender stereotypes. Northern people tend to be less influenced by stereotypes, claiming greater freedom in this regard. People from the South tend to be more influenced by these stereotypes and not question them, which is why the further one moves toward Southern Italy, the less people tend to respond to a survey that has the flavor of subverting a recognized order. According to a report of the National Institute of Statistics in Italy in 2019, gender stereotypes are present in 58.8% of the Italian population, being more prevalent among older and less educated individuals and more frequent in the South.

Moreover, like several surveys, a neutral response bias in which the participant chooses the neutral answer every time (“somewhat agree” or “slightly disagree,” in our study), indicating low interest in the survey and looking to answer questions as quickly as possible, can have affected our survey. In the so-called sensitive surveys, the neutral answers simultaneously indicate a desire to contribute to the survey but also the fear of going too far and leaving a sort of norm that respondents have in mind about how things go in the general population. To reduce this bias, we gave the possibility to answer “no” (“no difference between men and women”), putting the responders in the state to make a clear choice. The percentage results indicated that no neutral response bias influenced our survey.

We cannot exclude the socially desirable responding bias, the tendency of respondents to give predominantly positive self-descriptions. Indeed, due to the topic afforded in our survey, it is higher the probability to answer in a socially desirable way. Another limitation is that we did not examine or control the collected data for potential influences on mental health and psychological well-being, such as symptoms of anxiety, stress, and depression. This aspect is relevant and should be addressed in future research.

Despite these limitations and potential pitfalls, our survey has several strengths. It is the only national survey conducted on a large-scale population with a great sample size, providing a valuable view of the prevalence of gender stereotypes for Italy, although with limited national geographical representation. By including diverse age ranges, our survey may capture the varying perspectives of individuals from different generations. As mentioned, previous research on gender stereotypes in Italy has been conducted on small samples, in specific cities and settings, focusing on particular stereotypical dimensions. By examining a broader population, our approach increases the generalizability of the findings to a wider Italian population.

Our results show the persistence of gender stereotypes in any fields investigated, although our cohort is predominantly composed of high educational level women living in the North of Italy. This demonstrates that the long-standing gender stereotypes are prevalent, pernicious, and, unfortunately, internalized at times even by successful women pushbacking and sabotaging them unconsciously. Gender stereotypes can restrict a woman’s or man’s ability to grow personally, pursue a career, and/or make decisions about their lives, becoming damaging. Harmful stereotypes support injustices, whether they are blatantly hostile (such as “Ironing is an activity for women”) or apparently innocuous (such as “Women are more cunning” or “Women are more sensitive than men”). Women are frequently targeted by prejudice due to gender stereotypes. Many rights, including the right to health, an adequate standard of living, education, marriage and family relationships, jobs’ career, salary equality, freedom of expression, political participation and representation, and freedom from gender-based violence, are all violated as a result. The expectations of how men and women should act are shaped by the long-standing gender roles that have been instilled throughout the world. These societal standards and expectations significantly impact mental health (Arcand et al. 2020; Rice et al. 2021), increasing the risk to develop anxiety and depressive disorders, which affect more women than men (Carvalho Silva et al. 2023; Hantsoo & Epperson 2017). Indeed, mental health disorders such as anxiety and major depressive disorder are more commonly diagnosed in women than in men, with these psychopathologies affecting females at almost twice the rate of males (Salk et al. 2017). The female lifespan is characterized by several distinct stages, each with a unique hormonal environment and psychosocial context that significantly influences the susceptibility to psychiatric disorders (Hantsoo & Epperson 2017). Prevalence, clinical presentation, and treatment approaches differ significantly between men and women due to sex-related differences that are influenced by developmental stage, reproductive events, and hormonal status (Hantsoo & Epperson 2017). Although the role of biological factors has been explored, including physiological reactivity and hormonal factors, gender roles and stereotypes should also be considered (Arcand et al. 2020). For individuals who do not conform to traditional gender roles, the experience of depression and anxiety can be compounded by identity conflict and social stigma. It can be difficult for non-conforming people to balance their self-identity with society standards (Mousavi et al. 2019). They may feel misunderstood or rejected by their groups, which can lead to a strong sense of loneliness as a result of this conflict. Depression and anxiety problems can be made worse by social stigmatization of people who question conventional gender roles. Chronic stress and mental suffering might result from the dread of criticism, prejudice, or violence. Recognizing and challenging these stereotypes are essential for promoting gender equality and improving women’s mental well-being.

Despite the fact that our cohort is primarily made up of highly educated women who live in the northern part of Italy, we were able to achieve substantial results regarding gender stereotypes. On one hand, our research clearly indicates that gender stereotypes are still strong, affecting any part of society. On the other hand, we probably catch only the tip of the iceberg. Indeed, certain groups of women, such as those from minority groups, those with disabilities, those from lower caste groups or with lower economic status, migrants, etc., may be disproportionately negatively affected by gender stereotypes when they are combined and intersected with other stereotypes, increasing the risk of loneliness and isolation and reducing their self-identity expression. Consequently, in order to reduce the risk of various women’s mental disorders and promote mental well-being, it is crucial to develop a society that values variety and offers people a safe environment to explore and express their true selves.

### Supplementary Information

Below is the link to the electronic supplementary material.

Supplementary file1 (DOCX 297 KB)

## Data Availability

Data are available upon request.
