# Supplementary material for: Everything changes but nothing changes: gender stereotypes in the Italian population
Source: Arch Womens Ment Health. 2024 Feb 6;27(4):537–45. doi: 10.1007/s00737-024-01437-1 (PMC11230945; doi:10.1007/s00737-024-01437-1)
Supplement: Supplementary file 1 — Supplementary file1 (DOCX 297 KB) [file 737_2024_1437_MOESM1_ESM.docx]

**SUPPLEMENTARY TABLES**

**Table 1Sa:** Stratification analyses conducted between each question composing the latent construct “Home and family activities” (total of 11 questions) and age categories. Results containing p-values < 0.05 are highlighted in bold.

|  | **15-18 (N=74)** | **19-22 (N=223)** | **23-30 (N=347)** | **31-40 (N=336)** | **41-50 (N=304)** | **51-60 (N=296)** | **61-70 (N=214)** | **Over 70 (N=50)** | **p-value** |
| --- | --- | --- | --- | --- | --- | --- | --- | --- | --- |
| **Sewing is an activity for men** | | | | | | | | | |
| Completely agree | 0 (0.0%) | 0 (0.0%) | 0 (0.0%) | 0 (0.0%) | 3 (1.0%) | 0 (0.0%) | 0 (0.0%) | 0 (0.0%) | 0.539 |
| Strongly agree | 1 (1.4%) | 1 (0.4%) | 2 (0.6%) | 4 (1.2%) | 0 (0.0%) | 2 (0.7%) | 1 (0.5%) | 1 (2.0%) |  |
| Somewhat agree | 1 (1.4%) | 4 (1.8%) | 5 (1.4%) | 6 (1.8%) | 6 (2.0%) | 4 (1.4%) | 3 (1.4%) | 0 (0.0%) |  |
| Slightly disagree | 13 (17.6%) | 48 (21.5%) | 83 (23.9%) | 78 (23.2%) | 61 (20.1%) | 50 (16.9%) | 46 (21.5%) | 11 (22.0%) |  |
| Strongly disagree | 27 (36.5%) | 35 (15.7%) | 41 (11.8%) | 44 (13.1%) | 73 (24.0%) | 64 (21.6%) | 49 (22.9%) | 15 (30.0%) |  |
| No difference between men and women | 32 (43.2%) | 135 (60.5%) | 216 (62.2%) | 204 (60.7%) | 161 (53.0%) | 176 (59.5%) | 115 (53.7%) | 23 (46.0%) |  |
| *% Total of agreement** | *2.8%* | *2.2%* | *2.0%* | *3.0%* | *3.0%* | *2.1%* | *1.9%* | *2.0%* |  |
| **Ironing is an activity for women** | | | | | | | | | |
| Completely agree | 13 (17.6%) | 4 (1.8%) | 3 (0.9%) | 1 (0.3%) | 5 (1.6%) | 2 (0.7%) | 2 (0.9%) | 0 (0.0%) | **< 0.001** |
| Strongly agree | 7 (9.5%) | 4 (1.8%) | 6 (1.7%) | 10 (3.0%) | 5 (1.6%) | 2 (0.7%) | 2 (0.9%) | 4 (8.0%) |  |
| Somewhat agree | 14 (18.9%) | 18 (8.1%) | 24 (6.9%) | 28 (8.3%) | 42 (13.8%) | 24 (8.1%) | 24 (11.2%) | 7 (14.0%) |  |
| Slightly disagree | 2 (2.7%) | 25 (11.2%) | 27 (7.8%) | 46 (13.7%) | 32 (10.5%) | 44 (14.9%) | 37 (17.3%) | 7 (14.0%) |  |
| Strongly disagree | 12 (16.2%) | 51 (22.9%) | 82 (23.6%) | 73 (21.7%) | 86 (28.3%) | 92 (31.1%) | 71 (33.2%) | 13 (26.0%) |  |
| No difference between men and women | 26 (35.1%) | 121 (54.3%) | 205 (59.1%) | 178 (53.0%) | 134 (44.1%) | 132 (44.6%) | 78 (36.4%) | 19 (38.0%) |  |
| *% Total of agreement** | *46.0%* | *11.7%* | *9.5%* | *11.6%* | *17.0%* | *9.5%* | *13.0%* | *22.0%* |  |
| **It is men, more than women, who have to support the family** | | | | | | | | | |
| Completely agree | 9 (12.2%) | 1 (0.4%) | 2 (0.6%) | 0 (0.0%) | 3 (1.0%) | 4 (1.4%) | 0 (0.0%) | 1 (2.0%) | **< 0.001** |
| Strongly agree | 6 (8.1%) | 3 (1.3%) | 4 (1.2%) | 2 (0.6%) | 3 (1.0%) | 3 (1.0%) | 1 (0.5%) | 3 (6.0%) |  |
| Somewhat agree | 7 (9.5%) | 15 (6.7%) | 13 (3.7%) | 9 (2.7%) | 22 (7.2%) | 11 (3.7%) | 12 (5.6%) | 3 (6.0%) |  |
| Slightly disagree | 10 (13.5%) | 16 (7.2%) | 18 (5.2%) | 35 (10.4%) | 25 (8.2%) | 29 (9.8%) | 34 (15.9%) | 5 (10.0%) |  |
| Strongly disagree | 14 (18.9%) | 52 (23.3%) | 107 (30.8%) | 95 (28.3%) | 91 (29.9%) | 87 (29.4%) | 62 (29.0%) | 17 (34.0%) |  |
| No difference between men and women | 28 (37.8%) | 136 (61.0%) | 203 (58.5%) | 195 (58.0%) | 160 (52.6%) | 162 (54.7%) | 105 (49.1%) | 21 (42.0%) |  |
| *% Total of agreement** | *29.8%* | *8.4%* | *5.5%* | *3.3%* | *9.2%* | *6.1%* | *6.1%* | *14.0%* |  |
| **Men drive better than women** | | | | | | | | | |
| Completely agree | 20 (27.0%) | 15 (6.7%) | 5 (1.4%) | 11 (3.3%) | 14 (4.6%) | 6 (2.0%) | 4 (1.9%) | 0 (0.0%) | **< 0.001** |
| Strongly agree | 9 (12.2%) | 16 (7.2%) | 12 (3.5%) | 10 (3.0%) | 9 (3.0%) | 5 (1.7%) | 3 (1.4%) | 2 (4.0%) |  |
| Somewhat agree | 6 (8.1%) | 29 (13.0%) | 52 (15.0%) | 57 (17.0%) | 44 (14.5%) | 31 (10.5%) | 17 (7.9%) | 3 (6.0%) |  |
| Slightly disagree | 5 (6.8%) | 19 (8.5%) | 46 (13.3%) | 44 (13.1%) | 30 (9.9%) | 43 (14.5%) | 26 (12.1%) | 4 (8.0%) |  |
| Strongly disagree | 13 (17.6%) | 38 (17.0%) | 68 (19.6%) | 65 (19.3%) | 89 (29.3%) | 88 (29.7%) | 75 (35.0%) | 22 (44.0%) |  |
| No difference between men and women | 21 (28.4%) | 106 (47.5%) | 164 (47.3%) | 149 (44.3%) | 118 (38.8%) | 123 (41.6%) | 89 (41.6%) | 19 (38.0%) |  |
| *% Total of agreement** | *47.3%* | *26.9%* | *19.9%* | *23.3%* | *22.1%* | *14.2%* | *11.2%* | *10.0%* |  |
| **It is men, more than women, who have to make the most important decisions concerning the family** | | | | | | | | | |
| Completely agree | 6 (8.1%) | 0 (0.0%) | 1 (0.3%) | 0 (0.0%) | 2 (0.7%) | 1 (0.3%) | 1 (0.5%) | 0 (0.0%) | **< 0.001** |
| Strongly agree | 4 (5.4%) | 2 (0.9%) | 1 (0.3%) | 1 (0.3%) | 1 (0.3%) | 1 (0.3%) | 0 (0.0%) | 2 (4.0%) |  |
| Somewhat agree | 10 (13.5%) | 3 (1.3%) | 1 (0.3%) | 4 (1.2%) | 5 (1.6%) | 3 (1.0%) | 8 (3.7%) | 2 (4.0%) |  |
| Slightly disagree | 6 (8.1%) | 8 (3.6%) | 7 (2.0%) | 17 (5.1%) | 20 (6.6%) | 21 (7.1%) | 15 (7.0%) | 1 (2.0%) |  |
| Strongly disagree | 18 (24.3%) | 66 (29.6%) | 112 (32.3%) | 106 (31.5%) | 118 (38.8%) | 123 (41.6%) | 96 (44.9%) | 27 (54.0%) |  |
| No difference between men and women | 30 (40.5%) | 144 (64.6%) | 225 (64.8%) | 208 (61.9%) | 158 (52.0%) | 147 (49.7%) | 94 (43.9%) | 18 (36.0%) |  |
| *% Total of agreement** | *27.0%* | *2.2%* | *0.9%* | *1.5%* | *2.6%* | *1.6%* | *4.2%* | *8.0%* |  |
| **It is women, more than men, who have to cook at home** | | | | | | | | | |
| Completely agree | 15 (20.3%) | 2 (0.9%) | 1 (0.3%) | 1 (0.3%) | 0 (0.0%) | 0 (0.0%) | 0 (0.0%) | 0 (0.0%) | **< 0.001** |
| Strongly agree | 5 (6.8%) | 2 (0.9%) | 0 (0.0%) | 0 (0.0%) | 2 (0.7%) | 3 (1.0%) | 2 (0.9%) | 1 (2.0%) |  |
| Somewhat agree | 6 (8.1%) | 3 (1.3%) | 4 (1.2%) | 5 (1.5%) | 14 (4.6%) | 8 (2.7%) | 8 (3.7%) | 5 (10.0%) |  |
| Slightly disagree | 8 (10.8%) | 14 (6.3%) | 9 (2.6%) | 20 (6.0%) | 16 (5.3%) | 19 (6.4%) | 22 (10.3%) | 4 (8.0%) |  |
| Strongly disagree | 10 (13.5%) | 56 (25.1%) | 104 (30.0%) | 87 (25.9%) | 100 (32.9%) | 108 (36.5%) | 73 (34.1%) | 23 (46.0%) |  |
| No difference between men and women | 30 (40.5%) | 146 (65.5%) | 229 (66.0%) | 223 (66.4%) | 172 (56.6%) | 158 (53.4%) | 109 (50.9%) | 17 (34.0%) |  |
| *% Total of agreement** | *35.2%* | *3.1%* | *1.5%* | *1.8%* | *5.3%* | *3.7%* | *4.6%* | *12.0%* |  |
| **At home, it is men, more than women, who command** | | | | | | | | | |
| Completely agree | 8 (10.8%) | 2 (0.9%) | 1 (0.3%) | 0 (0.0%) | 0 (0.0%) | 0 (0.0%) | 0 (0.0%) | 0 (0.0%) | **< 0.001** |
| Strongly agree | 4 (5.4%) | 1 (0.4%) | 1 (0.3%) | 2 (0.6%) | 0 (0.0%) | 1 (0.3%) | 0 (0.0%) | 1 (2.0%) |  |
| Somewhat agree | 10 (13.5%) | 9 (4.0%) | 8 (2.3%) | 5 (1.5%) | 7 (2.3%) | 15 (5.1%) | 12 (5.6%) | 4 (8.0%) |  |
| Slightly disagree | 9 (12.2%) | 6 (2.7%) | 7 (2.0%) | 13 (3.9%) | 22 (7.2%) | 22 (7.4%) | 29 (13.6%) | 9 (18.0%) |  |
| Strongly disagree | 19 (25.7%) | 65 (29.1%) | 106 (30.5%) | 111 (33.0%) | 120 (39.5%) | 124 (41.9%) | 96 (44.9%) | 20 (40.0%) |  |
| No difference between men and women | 24 (32.4%) | 140 (62.8%) | 224 (64.6%) | 205 (61.0%) | 155 (51.0%) | 134 (45.3%) | 77 (36.0%) | 16 (32.0%) |  |
| *% Total of agreement** | *29.7%* | *5.3%* | *2.9%* | *2.1%* | *2.3%* | *5.4%* | *5.6%* | *10.0%* |  |
| **It is men, more than women, who are responsible for investing the family's savings** | | | | | | | | | |
| Completely agree | 7 (9.5%) | 1 (0.4%) | 1 (0.3%) | 0 (0.0%) | 0 (0.0%) | 2 (0.7%) | 0 (0.0%) | 0 (0.0%) | **< 0.001** |
| Strongly agree | 6 (8.1%) | 2 (0.9%) | 0 (0.0%) | 0 (0.0%) | 1 (0.3%) | 1 (0.3%) | 1 (0.5%) | 0 (0.0%) |  |
| Somewhat agree | 10 (13.5%) | 7 (3.1%) | 8 (2.3%) | 6 (1.8%) | 8 (2.6%) | 3 (1.0%) | 10 (4.7%) | 2 (4.0%) |  |
| Slightly disagree | 4 (5.4%) | 10 (4.5%) | 12 (3.5%) | 16 (4.8%) | 23 (7.6%) | 20 (6.8%) | 19 (8.9%) | 6 (12.0%) |  |
| Strongly disagree | 14 (18.9%) | 53 (23.8%) | 81 (23.3%) | 78 (23.2%) | 73 (24.0%) | 87 (29.4%) | 69 (32.2%) | 15 (30.0%) |  |
| No difference between men and women | 33 (44.6%) | 150 (67.3%) | 245 (70.6%) | 236 (70.2%) | 199 (65.5%) | 183 (61.8%) | 115 (53.7%) | 27 (54.0%) |  |
| *% Total of agreement** | *31.1%* | *4.4%* | *2.6%* | *1.8%* | *2.9%* | *2.0%* | *5.2%* | *4.0%* |  |
| **It is women, more than men, who must take care of the children** | | | | | | | | | |
| Completely agree | 8 (10.8%) | 3 (1.3%) | 2 (0.6%) | 3 (0.9%) | 1 (0.3%) | 1 (0.3%) | 1 (0.5%) | 0 (0.0%) | **< 0.001** |
| Strongly agree | 8 (10.8%) | 2 (0.9%) | 2 (0.6%) | 2 (0.6%) | 4 (1.3%) | 5 (1.7%) | 5 (2.3%) | 3 (6.0%) |  |
| Somewhat agree | 11 (14.9%) | 18 (8.1%) | 15 (4.3%) | 13 (3.9%) | 30 (9.9%) | 15 (5.1%) | 18 (8.4%) | 7 (14.0%) |  |
| Slightly disagree | 6 (8.1%) | 24 (10.8%) | 22 (6.3%) | 34 (10.1%) | 35 (11.5%) | 33 (11.1%) | 29 (13.6%) | 8 (16.0%) |  |
| Strongly disagree | 9 (12.2%) | 52 (23.3%) | 83 (23.9%) | 82 (24.4%) | 83 (27.3%) | 94 (31.8%) | 71 (33.2%) | 11 (22.0%) |  |
| No difference between men and women | 32 (43.2%) | 124 (55.6%) | 223 (64.3%) | 202 (60.1%) | 151 (49.7%) | 148 (50.0%) | 90 (42.1%) | 21 (42.0%) |  |
| *% Total of agreement** | *36.5%* | *10.3%* | *5.5%* | *5.4%* | *11.5%* | *7.1%* | *11.2%* | *20.0%* |  |
| **It is men, more than women, who have to clean the house** | | | | | | | | | |
| Completely agree | 2 (2.7%) | 0 (0.0%) | 1 (0.3%) | 1 (0.3%) | 1 (0.3%) | 3 (1.0%) | 0 (0.0%) | 0 (0.0%) | **< 0.001** |
| Strongly agree | 2 (2.7%) | 0 (0.0%) | 2 (0.6%) | 1 (0.3%) | 0 (0.0%) | 1 (0.3%) | 1 (0.5%) | 0 (0.0%) |  |
| Somewhat agree | 3 (4.1%) | 4 (1.8%) | 2 (0.6%) | 1 (0.3%) | 7 (2.3%) | 4 (1.4%) | 4 (1.9%) | 1 (2.0%) |  |
| Slightly disagree | 13 (17.6%) | 15 (6.7%) | 14 (4.0%) | 15 (4.5%) | 26 (8.6%) | 17 (5.7%) | 21 (9.8%) | 5 (10.0%) |  |
| Strongly disagree | 18 (24.3%) | 35 (15.7%) | 52 (15.0%) | 31 (9.2%) | 38 (12.5%) | 43 (14.5%) | 31 (14.5%) | 10 (20.0%) |  |
| No difference between men and women | 36 (48.6%) | 169 (75.8%) | 276 (79.5%) | 287 (85.4%) | 232 (76.3%) | 228 (77.0%) | 157 (73.4%) | 34 (68.0%) |  |
| *% Total of agreement** | *9.5%* | *1.8%* | *1.5%* | *0.9%* | *2.6%* | *2.7%* | *2.4%* | *2.0%* |  |
| **It is men, more than women, who have to help the children with their homework** | | | | | | | | | |
| Completely agree | 2 (2.7%) | 2 (0.9%) | 0 (0.0%) | 0 (0.0%) | 1 (0.3%) | 1 (0.3%) | 0 (0.0%) | 0 (0.0%) | **< 0.001** |
| Strongly agree | 2 (2.7%) | 1 (0.4%) | 2 (0.6%) | 0 (0.0%) | 1 (0.3%) | 0 (0.0%) | 0 (0.0%) | 0 (0.0%) |  |
| Somewhat agree | 9 (12.2%) | 1 (0.4%) | 0 (0.0%) | 3 (0.9%) | 5 (1.6%) | 1 (0.3%) | 4 (1.9%) | 0 (0.0%) |  |
| Slightly disagree | 7 (9.5%) | 12 (5.4%) | 11 (3.2%) | 13 (3.9%) | 16 (5.3%) | 13 (4.4%) | 14 (6.5%) | 6 (12.0%) |  |
| Strongly disagree | 12 (16.2%) | 35 (15.7%) | 47 (13.5%) | 30 (8.9%) | 38 (12.5%) | 47 (15.9%) | 32 (15.0%) | 11 (22.0%) |  |
| No difference between men and women | 42 (56.8%) | 172 (77.1%) | 287 (82.7%) | 290 (86.3%) | 243 (79.9%) | 234 (79.1%) | 164 (76.6%) | 33 (66.0%) |  |
| *% Total of agreement** | *17.6%* | *1.7%* | *0.6%* | *0.9%* | *2.2%* | *0.6%* | *1.9%* | *0.0%* |  |

*Note: The total percentage of agreement was calculated by summing the percentages of responses “completely agree”, “strongly agree”, and “somewhat agree”. This value is reported only for descriptive purposes and was not considered for the statistical analysis.

**Table 1Sb:** Stratification analyses conducted between each question composing the latent construct “Home and family activities” (total of 11 questions) and gender categories. Results containing p-values < 0.05 are highlighted in bold.

|  | **Female (N=1292)** | **Male (N=534)** | **Other/Do not want to answer (N=18)** | **p-value** |
| --- | --- | --- | --- | --- |
| **Sewing is an activity for men** | | | | |
| Completely agree | 2 (0.2%) | 1 (0.2%) | 0 (0.0%) | **0.002** |
| Strongly agree | 5 (0.4%) | 7 (1.3%) | 0 (0.0%) |  |
| Somewhat agree | 17 (1.3%) | 12 (2.2%) | 0 (0.0%) |  |
| Slightly disagree | 259 (20.0%) | 129 (24.2%) | 2 (11.1%) |  |
| Strongly disagree | 243 (18.8%) | 102 (19.1%) | 3 (16.7%) |  |
| No difference between men and women | 766 (59.3%) | 283 (53.0%) | 13 (72.2%) |  |
| *% Total of agreement** | *1.9%* | *3.7%* | *0.0 %* |  |
| **Ironing is an activity for women** | | | | |
| Completely agree | 5 (0.4%) | 25 (4.7%) | 0 (0.0%) | **< 0.001** |
| Strongly agree | 17 (1.3%) | 22 (4.1%) | 1 (5.6%) |  |
| Somewhat agree | 106 (8.2%) | 75 (14.0%) | 0 (0.0%) |  |
| Slightly disagree | 152 (11.8%) | 68 (12.7%) | 0 (0.0%) |  |
| Strongly disagree | 398 (30.8%) | 75 (14.0%) | 7 (38.9%) |  |
| No difference between men and women | 614 (47.5%) | 269 (50.4%) | 10 (55.6%) |  |
| *% Total of agreement** | *9.9%* | *22.8%* | *5.6%* |  |
| **It is men, more than women, who have to support the family** | | | | |
| Completely agree | 5 (0.4%) | 15 (2.8%) | 0 (0.0%) | **< 0.001** |
| Strongly agree | 7 (0.5%) | 17 (3.2%) | 1 (5.6%) |  |
| Somewhat agree | 48 (3.7%) | 44 (8.2%) | 0 (0.0%) |  |
| Slightly disagree | 114 (8.8%) | 58 (10.9%) | 0 (0.0%) |  |
| Strongly disagree | 424 (32.8%) | 94 (17.6%) | 7 (38.9%) |  |
| No difference between men and women | 694 (53.7%) | 306 (57.3%) | 10 (55.6%) |  |
| *% Total of agreement** | *4.6%* | *14.2%* | *5.6%* |  |
| **Men drive better than women** | | | | |
| Completely agree | 5 (0.4%) | 69 (12.9%) | 1 (5.6%) | **< 0.001** |
| Strongly agree | 19 (1.5%) | 47 (8.8%) | 0 (0.0%) |  |
| Somewhat agree | 121 (9.4%) | 117 (21.9%) | 1 (5.6%) |  |
| Slightly disagree | 161 (12.5%) | 56 (10.5%) | 0 (0.0%) |  |
| Strongly disagree | 407 (31.5%) | 46 (8.6%) | 5 (27.8%) |  |
| No difference between men and women | 579 (44.8%) | 199 (37.3%) | 11 (61.1%) |  |
| *% Total of agreement** | *11.3%* | *43.6%* | *11.2%* |  |
| **It is men, more than women, who have to make the most important decisions concerning the family** | | | | |
| Completely agree | 1 (0.1%) | 10 (1.9%) | 0 (0.0%) | 0.144 |
| Strongly agree | 2 (0.2%) | 9 (1.7%) | 1 (5.6%) |  |
| Somewhat agree | 16 (1.2%) | 20 (3.7%) | 0 (0.0%) |  |
| Slightly disagree | 55 (4.3%) | 40 (7.5%) | 0 (0.0%) |  |
| Strongly disagree | 546 (42.3%) | 111 (20.8%) | 9 (50.0%) |  |
| No difference between men and women | 672 (52.0%) | 344 (64.4%) | 8 (44.4%) |  |
| *% Total of agreement** | *1.5%* | *7.3%* | *5.6%* |  |
| **It is women, more than men, who have to cook at home** | | | | |
| Completely agree | 1 (0.1%) | 17 (3.2%) | 1 (5.6%) | **< 0.001** |
| Strongly agree | 5 (0.4%) | 10 (1.9%) | 0 (0.0%) |  |
| Somewhat agree | 20 (1.5%) | 33 (6.2%) | 0 (0.0%) |  |
| Slightly disagree | 66 (5.1%) | 46 (8.6%) | 0 (0.0%) |  |
| Strongly disagree | 442 (34.2%) | 112 (21.0%) | 7 (38.9%) |  |
| No difference between men and women | 758 (58.7%) | 316 (59.2%) | 10 (55.6%) |  |
| *% Total of agreement** | *2.0%* | *11.3%* | *5.6%* |  |
| **At home, it is men, more than women, who command** | | | | |
| Completely agree | 0 (0.0%) | 11 (2.1%) | 0 (0.0%) | **0.003** |
| Strongly agree | 4 (0.3%) | 5 (0.9%) | 1 (5.6%) |  |
| Somewhat agree | 41 (3.2%) | 28 (5.2%) | 1 (5.6%) |  |
| Slightly disagree | 68 (5.3%) | 47 (8.8%) | 2 (11.1%) |  |
| Strongly disagree | 509 (39.4%) | 147 (27.5%) | 5 (27.8%) |  |
| No difference between men and women | 670 (51.9%) | 296 (55.4%) | 9 (50.0%) |  |
| *% Total of agreement** | *3.5%* | *8.2%* | *11.2%* |  |
| **It is men, more than women, who are responsible for investing the family’s savings** | | | | |
| Completely agree | 2 (0.2%) | 9 (1.7%) | 0 (0.0%) | **0.001** |
| Strongly agree | 2 (0.2%) | 9 (1.7%) | 0 (0.0%) |  |
| Somewhat agree | 19 (1.5%) | 34 (6.4%) | 1 (5.6%) |  |
| Slightly disagree | 74 (5.7%) | 36 (6.7%) | 0 (0.0%) |  |
| Strongly disagree | 383 (29.6%) | 82 (15.4%) | 5 (27.8%) |  |
| No difference between men and women | 812 (62.8%) | 364 (68.2%) | 12 (66.7%) |  |
| *% Total of agreement** | *1.9%* | *9.8%* | *5.6%* |  |
| **It is women, more than men, who must take care of the children** | | | | |
| Completely agree | 6 (0.5%) | 13 (2.4%) | 0 (0.0%) | **< 0.001** |
| Strongly agree | 9 (0.7%) | 21 (3.9%) | 1 (5.6%) |  |
| Somewhat agree | 61 (4.7%) | 66 (12.4%) | 0 (0.0%) |  |
| Slightly disagree | 131 (10.1%) | 59 (11.0%) | 1 (5.6%) |  |
| Strongly disagree | 383 (29.6%) | 95 (17.8%) | 7 (38.9%) |  |
| No difference between men and women | 702 (54.3%) | 280 (52.4%) | 9 (50.0%) |  |
| *% Total of agreement** | *5.9%* | *18.7%* | *5.6%* |  |
| **It is men, more than women, who have to clean the house** | | | | |
| Completely agree | 5 (0.4%) | 2 (0.4%) | 1 (5.6%) | **< 0.001** |
| Strongly agree | 4 (0.3%) | 3 (0.6%) | 0 (0.0%) |  |
| Somewhat agree | 9 (0.7%) | 17 (3.2%) | 0 (0.0%) |  |
| Slightly disagree | 72 (5.6%) | 54 (10.1%) | 0 (0.0%) |  |
| Strongly disagree | 189 (14.6%) | 67 (12.5%) | 2 (11.1%) |  |
| No difference between men and women | 1013 (78.4%) | 391 (73.2%) | 15 (83.3%) |  |
| *% Total of agreement** | *1.4%* | *4.2%* | *5.6%* |  |
| **It is men, more than women, who must help the children with their homework** | | | | |
| Completely agree | 2 (0.2%) | 4 (0.7%) | 0 (0.0%) | **< 0.001** |
| Strongly agree | 2 (0.2%) | 4 (0.7%) | 0 (0.0%) |  |
| Somewhat agree | 9 (0.7%) | 14 (2.6%) | 0 (0.0%) |  |
| Slightly disagree | 51 (3.9%) | 40 (7.5%) | 1 (5.6%) |  |
| Strongly disagree | 180 (13.9%) | 69 (12.9%) | 3 (16.7%) |  |
| No difference between men and women | 1048 (81.1%) | 403 (75.5%) | 14 (77.8%) |  |
| *% Total of agreement** | *1.1%* | *4.0%* | *0.0%* |  |

*Note: The total percentage of agreement was calculated by summing the percentages of responses “completely agree”, “strongly agree”, and “somewhat agree”. This value is reported only for descriptive purposes and was not considered for the statistical analysis.

**Table 1Sc:** Stratification analyses conducted between each question composing the latent construct “Home and family activities” (total of 11 questions) and degree categories. Results containing p-values < 0.05 are highlighted in bold.

|  | **Elementary/Secondary school degrees (N=122)** | **High school degree (N=662)** | **University degree (N=737)** | **Post-University degree (N=323)** | **p-value** |
| --- | --- | --- | --- | --- | --- |
| **Sewing is an activity for men** | | | | | |
| Completely agree | 1 (0.8%) | 0 (0.0%) | 0 (0.0%) | 2 (0.6%) | 0.674 |
| Strongly agree | 1 (0.8%) | 6 (0.9%) | 4 (0.5%) | 1 (0.3%) |  |
| Somewhat agree | 2 (1.6%) | 9 (1.4%) | 15 (2.0%) | 3 (0.9%) |  |
| Slightly disagree | 20 (16.4%) | 125 (18.9%) | 167 (22.7%) | 78 (24.1%) |  |
| Strongly disagree | 35 (28.7%) | 136 (20.5%) | 123 (16.7%) | 54 (16.7%) |  |
| No difference between men and women | 63 (51.6%) | 386 (58.3%) | 428 (58.1%) | 185 (57.3%) |  |
| *% Total of agreement** | *3.2%* | *2.3%* | *2.5%* | *1.8%* |  |
| **Ironing is an activity for women** | | | | | |
| Completely agree | 13 (10.7%) | 11 (1.7%) | 2 (0.3%) | 4 (1.2%) | **<0.001** |
| Strongly agree | 13 (10.7%) | 11 (1.7%) | 11 (1.5%) | 5 (1.5%) |  |
| Somewhat agree | 15 (12.3%) | 72 (10.9%) | 65 (8.8%) | 29 (9.0%) |  |
| Slightly disagree | 10 (8.2%) | 73 (11.0%) | 93 (12.6%) | 44 (13.6%) |  |
| Strongly disagree | 24 (19.7%) | 182 (27.5%) | 196 (26.6%) | 78 (24.1%) |  |
| No difference between men and women | 47 (38.5%) | 313 (47.3%) | 370 (50.2%) | 163 (50.5%) |  |
| *% Total of agreement** | *33.7%* | *14.3%* | *10.6%* | *11.7%* |  |
| **It is men, more than women, who have to support the family** | | | | | |
| Completely agree | 11 (9.0%) | 6 (0.9%) | 1 (0.1%) | 2 (0.6%) | **<0.001** |
| Strongly agree | 8 (6.6%) | 10 (1.5%) | 4 (0.5%) | 3 (0.9%) |  |
| Somewhat agree | 12 (9.8%) | 39 (5.9%) | 28 (3.8%) | 13 (4.0%) |  |
| Slightly disagree | 12 (9.8%) | 56 (8.5%) | 82 (11.1%) | 22 (6.8%) |  |
| Strongly disagree | 18 (14.8%) | 178 (26.9%) | 224 (30.4%) | 105 (32.5%) |  |
| No difference between men and women | 61 (50.0%) | 373 (56.3%) | 398 (54.0%) | 178 (55.1%) |  |
| *% Total of agreement** | *25.4%* | *8.3%* | *4.4%* | *5.5%* |  |
| **Men drive better than women** | | | | | |
| Completely agree | 22 (18.0%) | 28 (4.2%) | 14 (1.9%) | 11 (3.4%) | **< 0.001** |
| Strongly agree | 9 (7.4%) | 29 (4.4%) | 19 (2.6%) | 9 (2.8%) |  |
| Somewhat agree | 15 (12.3%) | 88 (13.3%) | 93 (12.6%) | 43 (13.3%) |  |
| Slightly disagree | 6 (4.9%) | 63 (9.5%) | 100 (13.6%) | 48 (14.9%) |  |
| Strongly disagree | 24 (19.7%) | 170 (25.7%) | 197 (26.7%) | 67 (20.7%) |  |
| No difference between men and women | 46 (37.7%) | 284 (42.9%) | 314 (42.6%) | 145 (44.9%) |  |
| *% Total of agreement** | *37.7%* | *21.9%* | *17.1%* | *19.5%* |  |
| **It is men, more than women, who have to make the most important decisions concerning the family** | | | | | |
| Completely agree | 6 (4.9%) | 2 (0.3%) | 0 (0.0%) | 3 (0.9%) | **< 0.001** |
| Strongly agree | 7 (5.7%) | 2 (0.3%) | 1 (0.1%) | 2 (0.6%) |  |
| Somewhat agree | 9 (7.4%) | 20 (3.0%) | 6 (0.8%) | 1 (0.3%) |  |
| Slightly disagree | 9 (7.4%) | 39 (5.9%) | 33 (4.5%) | 14 (4.3%) |  |
| Strongly disagree | 30 (24.6%) | 221 (33.4%) | 284 (38.5%) | 131 (40.6%) |  |
| No difference between men and women | 61 (50.0%) | 378 (57.1%) | 413 (56.0%) | 172 (53.3%) |  |
| *% Total of agreement** | *18.0%* | *3.6%* | *0.9%* | *1.8%* |  |
| **It is women, more than men, who have to cook at home** | | | | | |
| Completely agree | 14 (11.5%) | 2 (0.3%) | 1 (0.1%) | 2 (0.6%) | **< 0.001** |
| Strongly agree | 5 (4.1%) | 7 (1.1%) | 2 (0.3%) | 1 (0.3%) |  |
| Somewhat agree | 14 (11.5%) | 20 (3.0%) | 12 (1.6%) | 7 (2.2%) |  |
| Slightly disagree | 7 (5.7%) | 48 (7.3%) | 43 (5.8%) | 14 (4.3%) |  |
| Strongly disagree | 22 (18.0%) | 194 (29.3%) | 236 (32.0%) | 109 (33.7%) |  |
| No difference between men and women | 60 (49.2%) | 391 (59.1%) | 443 (60.1%) | 190 (58.8%) |  |
| *% Total of agreement** | *27.1%* | *4.4%* | *2.0%* | *3.1%* |  |
| **At home, it is men, more than women, who command** | | | | | |
| Completely agree | 7 (5.7%) | 2 (0.3%) | 0 (0.0%) | 2 (0.6%) | **< 0.001** |
| Strongly agree | 4 (3.3%) | 2 (0.3%) | 3 (0.4%) | 1 (0.3%) |  |
| Somewhat agree | 9 (7.4%) | 31 (4.7%) | 23 (3.1%) | 7 (2.2%) |  |
| Slightly disagree | 14 (11.5%) | 48 (7.3%) | 36 (4.9%) | 19 (5.9%) |  |
| Strongly disagree | 36 (29.5%) | 228 (34.4%) | 278 (37.7%) | 119 (36.8%) |  |
| No difference between men and women | 52 (42.6%) | 351 (53.0%) | 397 (53.9%) | 175 (54.2%) |  |
| *% Total of agreement** | *16.4%* | *5.3%* | *3.5%* | *3.1%* |  |
| **It is men, more than women, who are responsible for investing the family's savings** | | | | | |
| Completely agree | 7 (5.7%) | 2 (0.3%) | 1 (0.1%) | 1 (0.3%) | **< 0.001** |
| Strongly agree | 6 (4.9%) | 5 (0.8%) | 0 (0.0%) | 0 (0.0%) |  |
| Somewhat agree | 13 (10.7%) | 20 (3.0%) | 16 (2.2%) | 5 (1.5%) |  |
| Slightly disagree | 5 (4.1%) | 47 (7.1%) | 40 (5.4%) | 18 (5.6%) |  |
| Strongly disagree | 22 (18.0%) | 167 (25.2%) | 188 (25.5%) | 93 (28.8%) |  |
| No difference between men and women | 69 (56.6%) | 421 (63.6%) | 492 (66.8%) | 206 (63.8%) |  |
| *% Total of agreement** | *21.3%* | *4.1%* | *2.3%* | *1.8%* |  |
| **It is women, more than men, who must take care of the children** | | | | | |
| Completely agree | 8 (6.6%) | 9 (1.4%) | 1 (0.1%) | 1 (0.3%) | **< 0.001** |
| Strongly agree | 9 (7.4%) | 12 (1.8%) | 8 (1.1%) | 2 (0.6%) |  |
| Somewhat agree | 15 (12.3%) | 52 (7.9%) | 41 (5.6%) | 19 (5.9%) |  |
| Slightly disagree | 10 (8.2%) | 73 (11.0%) | 64 (8.7%) | 44 (13.6%) |  |
| Strongly disagree | 16 (13.1%) | 165 (24.9%) | 221 (30.0%) | 83 (25.7%) |  |
| No difference between men and women | 64 (52.5%) | 351 (53.0%) | 402 (54.5%) | 174 (53.9%) |  |
| *% Total of agreement** | *26.3%* | *11.1%* | *6.8%* | *6.8%* |  |
| **It is men, more than women, who have to clean the house** | | | | | |
| Completely agree | 2 (1.6%) | 3 (0.5%) | 1 (0.1%) | 2 (0.6%) | **< 0.001** |
| Strongly agree | 2 (1.6%) | 1 (0.2%) | 3 (0.4%) | 1 (0.3%) |  |
| Somewhat agree | 5 (4.1%) | 12 (1.8%) | 8 (1.1%) | 1 (0.3%) |  |
| Slightly disagree | 17 (13.9%) | 57 (8.6%) | 34 (4.6%) | 18 (5.6%) |  |
| Strongly disagree | 21 (17.2%) | 100 (15.1%) | 95 (12.9%) | 42 (13.0%) |  |
| No difference between men and women | 75 (61.5%) | 489 (73.9%) | 596 (80.9%) | 259 (80.2%) |  |
| *% Total of agreement** | *7.3%* | *2.5%* | *1.6%* | *1.2%* |  |
| **It is men, more than women, who have to help the children with their homework** | | | | | |
| Completely agree | 2 (1.6%) | 2 (0.3%) | 1 (0.1%) | 1 (0.3%) | **< 0.001** |
| Strongly agree | 3 (2.5%) | 1 (0.2%) | 1 (0.1%) | 1 (0.3%) |  |
| Somewhat agree | 10 (8.2%) | 6 (0.9%) | 6 (0.8%) | 1 (0.3%) |  |
| Slightly disagree | 13 (10.7%) | 36 (5.4%) | 31 (4.2%) | 12 (3.7%) |  |
| Strongly disagree | 14 (11.5%) | 102 (15.4%) | 93 (12.6%) | 43 (13.3%) |  |
| No difference between men and women | 80 (65.6%) | 515 (77.8%) | 605 (82.1%) | 265 (82.0%) |  |
| *% Total of agreement** | *12.3%* | *1.4%* | *1.0%* | *0.9%* |  |

*Note: The total percentage of agreement was calculated by summing the percentages of responses “completely agree”, “strongly agree”, and “somewhat agree”. This value is reported only for descriptive purposes and was not considered for the statistical analysis.

**Table 2Sa:** Stratification analyses conducted between each question composing the latent construct “Games” (total of 7 questions) and age categories. Results containing p-values < 0.05 are highlighted in bold.

|  | **15-18 (N=74)** | **19-22 (N=223)** | **23-30 (N=347)** | **31-40 (N=336)** | **41-50 (N=304)** | **51-60 (N=296)** | **61-70 (N=214)** | **Over 70 (N=50)** | **p-value** |
| --- | --- | --- | --- | --- | --- | --- | --- | --- | --- |
| **Videogames are for males** | | | | | | | | | |
| Completely agree | 4 (5.4%) | 1 (0.4%) | 0 (0.0%) | 0 (0.0%) | 2 (0.7%) | 0 (0.0%) | 2 (0.9%) | 0 (0.0%) | **< 0.001** |
| Strongly agree | 4 (5.4%) | 3 (1.3%) | 2 (0.6%) | 8 (2.4%) | 11 (3.6%) | 7 (2.4%) | 4 (1.9%) | 1 (2.0%) |  |
| Somewhat agree | 14 (18.9%) | 20 (9.0%) | 22 (6.3%) | 30 (8.9%) | 32 (10.5%) | 37 (12.5%) | 25 (11.7%) | 3 (6.0%) |  |
| Slightly disagree | 10 (13.5%) | 41 (18.4%) | 68 (19.6%) | 49 (14.6%) | 49 (16.1%) | 42 (14.2%) | 36 (16.8%) | 6 (12.0%) |  |
| Strongly disagree | 12 (16.2%) | 65 (29.1%) | 82 (23.6%) | 86 (25.6%) | 77 (25.3%) | 88 (29.7%) | 52 (24.3%) | 14 (28.0%) |  |
| No difference between men and women | 30 (40.5%) | 93 (41.7%) | 173 (49.9%) | 163 (48.5%) | 133 (43.8%) | 122 (41.2%) | 95 (44.4%) | 26 (52.0%) |  |
| *% Total of agreement** | *29.7%* | *10.7%* | *6.9%* | *11.3%* | *14.8%* | *14.9%* | *14.5%* | *8.0%* |  |
| **The toy cars are a game for girls** | | | | | | | | | |
| Completely agree | 2 (2.7%) | 1 (0.4%) | 0 (0.0%) | 1 (0.3%) | 0 (0.0%) | 0 (0.0%) | 0 (0.0%) | 0 (0.0%) | **< 0.001** |
| Strongly agree | 0 (0.0%) | 2 (0.9%) | 1 (0.3%) | 4 (1.2%) | 1 (0.3%) | 1 (0.3%) | 0 (0.0%) | 1 (2.0%) |  |
| Somewhat agree | 2 (2.7%) | 8 (3.6%) | 9 (2.6%) | 7 (2.1%) | 8 (2.6%) | 7 (2.4%) | 10 (4.7%) | 1 (2.0%) |  |
| Slightly disagree | 15 (20.3%) | 39 (17.5%) | 51 (14.7%) | 60 (17.9%) | 72 (23.7%) | 69 (23.3%) | 58 (27.1%) | 14 (28.0%) |  |
| Strongly disagree | 22 (29.7%) | 26 (11.7%) | 48 (13.8%) | 36 (10.7%) | 57 (18.8%) | 56 (18.9%) | 35 (16.4%) | 11 (22.0%) |  |
| No difference between men and women | 33 (44.6%) | 147 (65.9%) | 238 (68.6%) | 228 (67.9%) | 166 (54.6%) | 163 (55.1%) | 111 (51.9%) | 23 (46.0%) |  |
| *% Total of agreement** | *5.4%* | *4.9%* | *2.9%* | *3.6%* | *2.9%* | *2.7%* | *4.7%* | *4.0%* |  |
| **Puzzles are a game for males** | | | | | | | | | |
| Completely agree | 0 (0.0%) | 0 (0.0%) | 0 (0.0%) | 0 (0.0%) | 0 (0.0%) | 0 (0.0%) | 0 (0.0%) | 0 (0.0%) | **< 0.001** |
| Strongly agree | 1 (1.4%) | 1 (0.4%) | 0 (0.0%) | 0 (0.0%) | 0 (0.0%) | 0 (0.0%) | 1 (0.5%) | 0 (0.0%) |  |
| Somewhat agree | 1 (1.4%) | 1 (0.4%) | 0 (0.0%) | 1 (0.3%) | 0 (0.0%) | 0 (0.0%) | 1 (0.5%) | 0 (0.0%) |  |
| Slightly disagree | 9 (12.2%) | 5 (2.2%) | 9 (2.6%) | 13 (3.9%) | 14 (4.6%) | 18 (6.1%) | 14 (6.5%) | 4 (8.0%) |  |
| Strongly disagree | 16 (21.6%) | 49 (22.0%) | 67 (19.3%) | 58 (17.3%) | 83 (27.3%) | 88 (29.7%) | 72 (33.6%) | 17 (34.0%) |  |
| No difference between men and women | 47 (63.5%) | 167 (74.9%) | 271 (78.1%) | 264 (78.6%) | 207 (68.1%) | 190 (64.2%) | 126 (58.9%) | 29 (58.0%) |  |
| *% Total of agreement** | *2.8%* | *0.8%* | *0.0%* | *0.3%* | *0.0%* | *0.0%* | *1.0%* | *0.0%* |  |
| **The tricycle is a game for boys** | | | | | | | | | |
| Completely agree | 3 (4.1%) | 0 (0.0%) | 0 (0.0%) | 0 (0.0%) | 0 (0.0%) | 0 (0.0%) | 0 (0.0%) | 0 (0.0%) | **< 0.001** |
| Strongly agree | 2 (2.7%) | 1 (0.4%) | 0 (0.0%) | 0 (0.0%) | 1 (0.3%) | 0 (0.0%) | 0 (0.0%) | 0 (0.0%) |  |
| Somewhat agree | 10 (13.5%) | 4 (1.8%) | 1 (0.3%) | 3 (0.9%) | 0 (0.0%) | 1 (0.3%) | 2 (0.9%) | 3 (6.0%) |  |
| Slightly disagree | 6 (8.1%) | 8 (3.6%) | 11 (3.2%) | 10 (3.0%) | 9 (3.0%) | 14 (4.7%) | 12 (5.6%) | 2 (4.0%) |  |
| Strongly disagree | 11 (14.9%) | 51 (22.9%) | 67 (19.3%) | 66 (19.6%) | 83 (27.3%) | 98 (33.1%) | 76 (35.5%) | 18 (36.0%) |  |
| No difference between men and women | 42 (56.8%) | 159 (71.3%) | 268 (77.2%) | 257 (76.5%) | 211 (69.4%) | 183 (61.8%) | 124 (57.9%) | 27 (54.0%) |  |
| *% Total of agreement** | *20.3%* | *2.2%* | *0.3%* | *0.9%* | *0.3%* | *0.3%* | *0.9%* | *6.0%* |  |
| **The bricks are a game for boys** | | | | | | | | | |
| Completely agree | 3 (4.1%) | 0 (0.0%) | 0 (0.0%) | 0 (0.0%) | 0 (0.0%) | 1 (0.3%) | 0 (0.0%) | 0 (0.0%) | **< 0.001** |
| Strongly agree | 9 (12.2%) | 0 (0.0%) | 0 (0.0%) | 3 (0.9%) | 3 (1.0%) | 0 (0.0%) | 0 (0.0%) | 1 (2.0%) |  |
| Somewhat agree | 7 (9.5%) | 8 (3.6%) | 9 (2.6%) | 7 (2.1%) | 8 (2.6%) | 5 (1.7%) | 7 (3.3%) | 3 (6.0%) |  |
| Slightly disagree | 9 (12.2%) | 15 (6.7%) | 17 (4.9%) | 20 (6.0%) | 14 (4.6%) | 25 (8.4%) | 13 (6.1%) | 4 (8.0%) |  |
| Strongly disagree | 13 (17.6%) | 44 (19.7%) | 58 (16.7%) | 55 (16.4%) | 65 (21.4%) | 82 (27.7%) | 60 (28.0%) | 13 (26.0%) |  |
| No difference between men and women | 33 (44.6%) | 156 (70.0%) | 263 (75.8%) | 251 (74.7%) | 214 (70.4%) | 183 (61.8%) | 134 (62.6%) | 29 (58.0%) |  |
| *% Total of agreement** | *25.8%* | *3.6%* | *2.6%* | *3.0%* | *3.6%* | *2.0%* | *3.3%* | *8.0%* |  |
| **Stuffed animals are a toy for boys** | | | | | | | | | |
| Completely agree | 2 (2.7%) | 1 (0.4%) | 1 (0.3%) | 0 (0.0%) | 1 (0.3%) | 0 (0.0%) | 2 (0.9%) | 0 (0.0%) | **< 0.001** |
| Strongly agree | 0 (0.0%) | 2 (0.9%) | 0 (0.0%) | 2 (0.6%) | 1 (0.3%) | 0 (0.0%) | 0 (0.0%) | 0 (0.0%) |  |
| Somewhat agree | 5 (6.8%) | 5 (2.2%) | 3 (0.9%) | 1 (0.3%) | 4 (1.3%) | 0 (0.0%) | 1 (0.5%) | 0 (0.0%) |  |
| Slightly disagree | 11 (14.9%) | 16 (7.2%) | 9 (2.6%) | 15 (4.5%) | 23 (7.6%) | 24 (8.1%) | 22 (10.3%) | 9 (18.0%) |  |
| Strongly disagree | 13 (17.6%) | 31 (13.9%) | 51 (14.7%) | 39 (11.6%) | 53 (17.4%) | 67 (22.6%) | 50 (23.4%) | 12 (24.0%) |  |
| No difference between men and women | 43 (58.1%) | 168 (75.3%) | 283 (81.6%) | 279 (83.0%) | 222 (73.0%) | 205 (69.3%) | 139 (65.0%) | 29 (58.0%) |  |
| *% Total of agreement** | *9.5%* | *3.5%* | *1.2%* | *0.9%* | *1.9%* | *0.0%* | *1.4%* | *0.0%* |  |
| **Playing with dolls is for girls** | | | | | | | | | |
| Completely agree | 21 (28.4%) | 6 (2.7%) | 6 (1.7%) | 11 (3.3%) | 10 (3.3%) | 7 (2.4%) | 6 (2.8%) | 5 (10.0%) | **< 0.001** |
| Strongly agree | 8 (10.8%) | 14 (6.3%) | 12 (3.5%) | 12 (3.6%) | 15 (4.9%) | 7 (2.4%) | 7 (3.3%) | 2 (4.0%) |  |
| Somewhat agree | 7 (9.5%) | 24 (10.8%) | 39 (11.2%) | 44 (13.1%) | 51 (16.8%) | 49 (16.6%) | 44 (20.6%) | 13 (26.0%) |  |
| Slightly disagree | 6 (8.1%) | 22 (9.9%) | 38 (11.0%) | 35 (10.4%) | 42 (13.8%) | 52 (17.6%) | 40 (18.7%) | 9 (18.0%) |  |
| Strongly disagree | 10 (13.5%) | 35 (15.7%) | 47 (13.5%) | 54 (16.1%) | 60 (19.7%) | 66 (22.3%) | 46 (21.5%) | 7 (14.0%) |  |
| No difference between men and women | 22 (29.7%) | 122 (54.7%) | 205 (59.1%) | 180 (53.6%) | 126 (41.4%) | 115 (38.9%) | 71 (33.2%) | 14 (28.0%) |  |
| *% Total of agreement** | *48.7%* | *19.8%* | *16.4%* | *20.0%* | *25.0%* | *21.4%* | *26.7%* | *40.0%* |  |

*Note: The total percentage of agreement was calculated by summing the percentages of responses “completely agree”, “strongly agree”, and “somewhat agree”. This value is reported only for descriptive purposes and was not considered for the statistical analysis.

**Table 2Sb:** Stratification analyses conducted between each question composing the latent construct “Games” (total of 7 questions) and gender categories. Results containing p-values < 0.05 are highlighted in bold.

|  | **Female (N=1292)** | **Male (N=534)** | **Other/Do not want to answer (N=18)** | **p-value** |
| --- | --- | --- | --- | --- |
| **Videogames are for males** | | | | |
| Completely agree | 4 (0.3%) | 5 (0.9%) | 0 (0.0%) | **< 0.001** |
| Strongly agree | 20 (1.5%) | 20 (3.7%) | 0 (0.0%) |  |
| Somewhat agree | 110 (8.5%) | 72 (13.5%) | 1 (5.6%) |  |
| Slightly disagree | 221 (17.1%) | 80 (15.0%) | 0 (0.0%) |  |
| Strongly disagree | 357 (27.6%) | 114 (21.3%) | 5 (27.8%) |  |
| No difference between men and women | 580 (44.9%) | 243 (45.5%) | 12 (66.7%) |  |
| *% Total of agreement** | *10.3%* | *18.1%* | *5.6%* |  |
| **The toy cars are a game for girls** | | | | |
| Completely agree | 2 (0.2%) | 1 (0.2%) | 1 (5.6%) | **< 0.001** |
| Strongly agree | 7 (0.5%) | 3 (0.6%) | 0 (0.0%) |  |
| Somewhat agree | 33 (2.6%) | 18 (3.4%) | 1 (5.6%) |  |
| Slightly disagree | 238 (18.4%) | 137 (25.7%) | 3 (16.7%) |  |
| Strongly disagree | 182 (14.1%) | 107 (20.0%) | 2 (11.1%) |  |
| No difference between men and women | 830 (64.2%) | 268 (50.2%) | 11 (61.1%) |  |
| *% Total of agreement** | *3.3%* | *4.2%* | *11.2%* |  |
| **Puzzles are a game for males** | | | | |
| Completely agree | 0 (0.0%) | 0 (0.0%) | 0 (0.0%) | **0.002** |
| Strongly agree | 2 (0.2%) | 1 (0.2%) | 0 (0.0%) |  |
| Somewhat agree | 1 (0.1%) | 3 (0.6%) | 0 (0.0%) |  |
| Slightly disagree | 45 (3.5%) | 40 (7.5%) | 1 (5.6%) |  |
| Strongly disagree | 340 (26.3%) | 106 (19.9%) | 4 (22.2%) |  |
| No difference between men and women | 904 (70.0%) | 384 (71.9%) | 13 (72.2%) |  |
| *% Total of agreement** | *0.3%* | *0.8%* | *0.0%* |  |
| **The tricycle is a game for boys** | | | | |
| Completely agree | 0 (0.0%) | 3 (0.6%) | 0 (0.0%) | **< 0.001** |
| Strongly agree | 2 (0.2%) | 2 (0.4%) | 0 (0.0%) |  |
| Somewhat agree | 4 (0.3%) | 19 (3.6%) | 1 (5.6%) |  |
| Slightly disagree | 33 (2.6%) | 39 (7.3%) | 0 (0.0%) |  |
| Strongly disagree | 374 (28.9%) | 92 (17.2%) | 4 (22.2%) |  |
| No difference between men and women | 879 (68.0%) | 379 (71.0%) | 13 (72.2%) |  |
| *% Total of agreement** | *0.5%* | *4.6%* | *5.6%* |  |
| **The bricks are a game for boys** | | | | |
| Completely agree | 1 (0.1%) | 3 (0.6%) | 0 (0.0%) | **< 0.001** |
| Strongly agree | 1 (0.1%) | 14 (2.6%) | 1 (5.6%) |  |
| Somewhat agree | 20 (1.5%) | 34 (6.4%) | 0 (0.0%) |  |
| Slightly disagree | 67 (5.2%) | 49 (9.2%) | 1 (5.6%) |  |
| Strongly disagree | 304 (23.5%) | 82 (15.4%) | 4 (22.2%) |  |
| No difference between men and women | 899 (69.6%) | 352 (65.9%) | 12 (66.7%) |  |
| *% Total of agreement** | *1.7%* | *9.6%* | *5.6%* |  |
| **Stuffed animals are a toy for boys** | | | | |
| Completely agree | 3 (0.2%) | 4 (0.7%) | 0 (0.0%) | **< 0.001** |
| Strongly agree | 4 (0.3%) | 1 (0.2%) | 0 (0.0%) |  |
| Somewhat agree | 10 (0.8%) | 9 (1.7%) | 0 (0.0%) |  |
| Slightly disagree | 58 (4.5%) | 71 (13.3%) | 0 (0.0%) |  |
| Strongly disagree | 230 (17.8%) | 82 (15.4%) | 4 (22.2%) |  |
| No difference between men and women | 987 (76.4%) | 367 (68.7%) | 14 (77.8%) |  |
| *% Total of agreement** | *1.3%* | *2.6%* | *0.0%* |  |
| **Playing with dolls is for girls** | | | | |
| Completely agree | 14 (1.1%) | 57 (10.7%) | 1 (5.6%) | **< 0.001** |
| Strongly agree | 30 (2.3%) | 47 (8.8%) | 0 (0.0%) |  |
| Somewhat agree | 150 (11.6%) | 119 (22.3%) | 2 (11.1%) |  |
| Slightly disagree | 182 (14.1%) | 60 (11.2%) | 2 (11.1%) |  |
| Strongly disagree | 275 (21.3%) | 46 (8.6%) | 4 (22.2%) |  |
| No difference between men and women | 641 (49.6%) | 205 (38.4%) | 9 (50.0%) |  |
| *% Total of agreement** | *15.0%* | *41.8%* | *16.7%* |  |

*Note: The total percentage of agreement was calculated by summing the percentages of responses “completely agree”, “strongly agree”, and “somewhat agree”. This value is reported only for descriptive purposes and was not considered for the statistical analysis.

**Table 2Sc:** Stratification analyses conducted between each question composing the latent construct “Games” (total of 7 questions) and degree categories. Results containing p-values < 0.05 are highlighted in bold.

|  | **Elementary/Secondary school degrees (N=122)** | **High school degree**  **(N=662)** | **University degree**  **(N=737)** | **Post-University degree (N=323)** | **p-value** |
| --- | --- | --- | --- | --- | --- |
| **Videogames are for males** | | | | | |
| Completely agree | 4 (3.3%) | 3 (0.5%) | 1 (0.1%) | 1 (0.3%) | **< 0.001** |
| Strongly agree | 7 (5.7%) | 11 (1.7%) | 14 (1.9%) | 8 (2.5%) |  |
| Somewhat agree | 15 (12.3%) | 67 (10.1%) | 64 (8.7%) | 37 (11.5%) |  |
| Slightly disagree | 12 (9.8%) | 95 (14.4%) | 147 (19.9%) | 47 (14.6%) |  |
| Strongly disagree | 25 (20.5%) | 176 (26.6%) | 198 (26.9%) | 77 (23.8%) |  |
| No difference between men and women | 59 (48.4%) | 310 (46.8%) | 313 (42.5%) | 153 (47.4%) |  |
| *% Total of agreement** | *21.3%* | *12.3%* | *10.7%* | *14.3%* |  |
| **The toy cars are a game for girls** | | | | | |
| Completely agree | 2 (1.6%) | 1 (0.2%) | 0 (0.0%) | 1 (0.3%) | **< 0.001** |
| Strongly agree | 0 (0.0%) | 1 (0.2%) | 6 (0.8%) | 3 (0.9%) |  |
| Somewhat agree | 7 (5.7%) | 18 (2.7%) | 23 (3.1%) | 4 (1.2%) |  |
| Slightly disagree | 17 (13.9%) | 139 (21.0%) | 147 (19.9%) | 75 (23.2%) |  |
| Strongly disagree | 32 (26.2%) | 110 (16.6%) | 103 (14.0%) | 46 (14.2%) |  |
| No difference between men and women | 64 (52.5%) | 393 (59.4%) | 458 (62.1%) | 194 (60.1%) |  |
| *% Total of agreement** | *7.3%* | *3.1%* | *3.6%* | *2.4%* |  |
| **Puzzles are a game for males** | | | | | |
| Completely agree | 0 (0.0%) | 0 (0.0%) | 0 (0.0%) | 0 (0.0%) | **0.036** |
| Strongly agree | 1 (0.8%) | 1 (0.2%) | 1 (0.1%) | 0 (0.0%) |  |
| Somewhat agree | 0 (0.0%) | 2 (0.3%) | 1 (0.1%) | 1 (0.3%) |  |
| Slightly disagree | 10 (8.2%) | 23 (3.5%) | 37 (5.0%) | 16 (5.0%) |  |
| Strongly disagree | 37 (30.3%) | 182 (27.5%) | 169 (22.9%) | 62 (19.2%) |  |
| No difference between men and women | 74 (60.7%) | 454 (68.6%) | 529 (71.8%) | 244 (75.5%) |  |
| *% Total of agreement** | *0.8%* | *0.5%* | *0.2%* | *0.3%* |  |
| **The tricycle is a game for boys** | | | | | |
| Completely agree | 2 (1.6%) | 1 (0.2%) | 0 (0.0%) | 0 (0.0%) | **< 0.001** |
| Strongly agree | 3 (2.5%) | 1 (0.2%) | 0 (0.0%) | 0 (0.0%) |  |
| Somewhat agree | 8 (6.6%) | 9 (1.4%) | 5 (0.7%) | 2 (0.6%) |  |
| Slightly disagree | 10 (8.2%) | 22 (3.3%) | 27 (3.7%) | 13 (4.0%) |  |
| Strongly disagree | 29 (23.8%) | 182 (27.5%) | 186 (25.2%) | 73 (22.6%) |  |
| No difference between men and women | 70 (57.4%) | 447 (67.5%) | 519 (70.4%) | 235 (72.8%) |  |
| *% Total of agreement** | *10.7%* | *1.8%* | *0.7%* | *0.6%* |  |
| **The bricks are a game for boys** | | | | | |
| Completely agree | 3 (2.5%) | 0 (0.0%) | 1 (0.1%) | 0 (0.0%) | **< 0.001** |
| Strongly agree | 8 (6.6%) | 4 (0.6%) | 3 (0.4%) | 1 (0.3%) |  |
| Somewhat agree | 8 (6.6%) | 21 (3.2%) | 19 (2.6%) | 6 (1.9%) |  |
| Slightly disagree | 14 (11.5%) | 36 (5.4%) | 45 (6.1%) | 22 (6.8%) |  |
| Strongly disagree | 24 (19.7%) | 157 (23.7%) | 152 (20.6%) | 57 (17.6%) |  |
| No difference between men and women | 65 (53.3%) | 444 (67.1%) | 517 (70.1%) | 237 (73.4%) |  |
| *% Total of agreement** | *15.7%* | *3.8%* | *3.1%* | *2.2%* |  |
| **Stuffed animals are a toy for boys** | | | | | |
| Completely agree | 2 (1.6%) | 4 (0.6%) | 0 (0.0%) | 1 (0.3%) | **0.002** |
| Strongly agree | 0 (0.0%) | 1 (0.2%) | 3 (0.4%) | 1 (0.3%) |  |
| Somewhat agree | 5 (4.1%) | 10 (1.5%) | 2 (0.3%) | 2 (0.6%) |  |
| Slightly disagree | 14 (11.5%) | 50 (7.6%) | 49 (6.6%) | 16 (5.0%) |  |
| Strongly disagree | 23 (18.9%) | 115 (17.4%) | 121 (16.4%) | 57 (17.6%) |  |
| No difference between men and women | 78 (63.9%) | 482 (72.8%) | 562 (76.3%) | 246 (76.2%) |  |
| *% Total of agreement** | *5.7%* | *2.3%* | *0.7%* | *1.2%* |  |
| **Playing with dolls is for girls** | | | | | |
| Completely agree | 21 (17.2%) | 28 (4.2%) | 13 (1.8%) | 10 (3.1%) | **< 0.001** |
| Strongly agree | 11 (9.0%) | 35 (5.3%) | 24 (3.3%) | 7 (2.2%) |  |
| Somewhat agree | 15 (12.3%) | 90 (13.6%) | 117 (15.9%) | 49 (15.2%) |  |
| Slightly disagree | 11 (9.0%) | 90 (13.6%) | 97 (13.2%) | 46 (14.2%) |  |
| Strongly disagree | 13 (10.7%) | 112 (16.9%) | 134 (18.2%) | 66 (20.4%) |  |
| No difference between men and women | 51 (41.8%) | 307 (46.4%) | 352 (47.8%) | 145 (44.9%) |  |
| *% Total of agreement** | *38.5%* | *23.1%* | *21.0%* | *20.5%* |  |

*Note: The total percentage of agreement was calculated by summing the percentages of responses “completely agree”, “strongly agree”, and “somewhat agree”. This value is reported only for descriptive purposes and was not considered for the statistical analysis.

**Table 3Sa:** Stratification analyses conducted between each question composing the latent construct “Moral judgements” (total of 3 questions) and age categories. Results containing p-values < 0.05 are highlighted in bold.

|  | **15-18 (N=74)** | **19-22 (N=223)** | **23-30 (N=347)** | **31-40 (N=336)** | **41-50 (N=304)** | **51-60 (N=296)** | **61-70 (N=214)** | **Over 70 (N=50)** | **p-value** |
| --- | --- | --- | --- | --- | --- | --- | --- | --- | --- |
| **In a couple, female infidelity is more serious than male infidelity** | | | | | | | | | |
| Completely agree | 8 (10.8%) | 1 (0.4%) | 1 (0.3%) | 0 (0.0%) | 2 (0.7%) | 1 (0.3%) | 3 (1.4%) | 1 (2.0%) | **< 0.001** |
| Strongly agree | 5 (6.8%) | 0 (0.0%) | 1 (0.3%) | 0 (0.0%) | 1 (0.3%) | 0 (0.0%) | 0 (0.0%) | 0 (0.0%) |  |
| Somewhat agree | 7 (9.5%) | 1 (0.4%) | 2 (0.6%) | 3 (0.9%) | 4 (1.3%) | 2 (0.7%) | 4 (1.9%) | 0 (0.0%) |  |
| Slightly disagree | 3 (4.1%) | 4 (1.8%) | 1 (0.3%) | 4 (1.2%) | 5 (1.6%) | 7 (2.4%) | 4 (1.9%) | 2 (4.0%) |  |
| Strongly disagree | 17 (23.0%) | 70 (31.4%) | 97 (28.0%) | 91 (27.1%) | 98 (32.2%) | 106 (35.8%) | 93 (43.5%) | 24 (48.0%) |  |
| No difference between men and women | 34 (45.9%) | 147 (65.9%) | 245 (70.6%) | 238 (70.8%) | 194 (63.8%) | 180 (60.8%) | 110 (51.4%) | 23 (46.0%) |  |
| *% Total of agreement** | *27.1%* | *0.8%* | *1.2%* | *0.9%* | *2.3%* | *1.0%* | *3.3%* | *2.0%* |  |
| **A woman who works will never be completely a good mother and partner** | | | | | | | | | |
| Completely agree | 1 (1.4%) | 0 (0.0%) | 0 (0.0%) | 2 (0.6%) | 0 (0.0%) | 1 (0.3%) | 0 (0.0%) | 0 (0.0%) | **< 0.001** |
| Strongly agree | 3 (4.1%) | 1 (0.4%) | 1 (0.3%) | 0 (0.0%) | 0 (0.0%) | 0 (0.0%) | 0 (0.0%) | 0 (0.0%) |  |
| Somewhat agree | 3 (4.1%) | 0 (0.0%) | 2 (0.6%) | 2 (0.6%) | 6 (2.0%) | 5 (1.7%) | 1 (0.5%) | 1 (2.0%) |  |
| Slightly disagree | 16 (21.6%) | 15 (6.7%) | 25 (7.2%) | 19 (5.7%) | 26 (8.6%) | 26 (8.8%) | 12 (5.6%) | 6 (12.0%) |  |
| Strongly disagree | 51 (68.9%) | 207 (92.8%) | 319 (91.9%) | 313 (93.2%) | 272 (89.5%) | 264 (89.2%) | 201 (93.9%) | 43 (86.0%) |  |
| No difference between men and women | 0 (0.0%) | 0 (0.0%) | 0 (0.0%) | 0 (0.0%) | 0 (0.0%) | 0 (0.0%) | 0 (0.0%) | 0 (0.0%) |  |
| *% Total of agreement** | *9.6%* | *0.4%* | *0.9%* | *1.2%* | *2.0%* | *2.0%* | *0.5%* | *2.0%* |  |
| **It is understandable that a man, more than a woman, can lose patience** | | | | | | | | | |
| Completely agree | 5 (6.8%) | 0 (0.0%) | 0 (0.0%) | 0 (0.0%) | 0 (0.0%) | 0 (0.0%) | 0 (0.0%) | 0 (0.0%) | **< 0.001** |
| Strongly agree | 6 (8.1%) | 2 (0.9%) | 3 (0.9%) | 1 (0.3%) | 0 (0.0%) | 1 (0.3%) | 3 (1.4%) | 1 (2.0%) |  |
| Somewhat agree | 10 (13.5%) | 6 (2.7%) | 5 (1.4%) | 4 (1.2%) | 6 (2.0%) | 3 (1.0%) | 8 (3.7%) | 1 (2.0%) |  |
| Slightly disagree | 6 (8.1%) | 15 (6.7%) | 14 (4.0%) | 7 (2.1%) | 7 (2.3%) | 12 (4.1%) | 15 (7.0%) | 7 (14.0%) |  |
| Strongly disagree | 16 (21.6%) | 70 (31.4%) | 113 (32.6%) | 109 (32.4%) | 110 (36.2%) | 117 (39.5%) | 87 (40.7%) | 25 (50.0%) |  |
| No difference between men and women | 31 (41.9%) | 130 (58.3%) | 212 (61.1%) | 215 (64.0%) | 181 (59.5%) | 163 (55.1%) | 101 (47.2%) | 16 (32.0%) |  |
| *% Total of agreement** | *28.4%* | *3.6%* | *2.3%* | *1.6%* | *2.0%* | *1.3%* | *5.1%* | *4.0%* |  |

*Note: The total percentage of agreement was calculated by summing the percentages of responses “completely agree”, “strongly agree”, and “somewhat agree”. This value is reported only for descriptive purposes and was not considered for the statistical analysis.

**Table 3Sb:** Stratification analyses conducted between each question composing the latent construct “Moral judgements” (total of 3 questions) and gender categories. Results containing p-values < 0.05 are highlighted in bold.

|  | **Female (N=1292)** | **Male (N=534)** | **Other/Do not want to answer (N=18)** | **p-value** |
| --- | --- | --- | --- | --- |
| **In a couple, female infidelity is more serious than male infidelity** | | | | |
| Completely agree | 5 (0.4%) | 11 (2.1%) | 1 (5.6%) | **0.003** |
| Strongly agree | 1 (0.1%) | 6 (1.1%) | 0 (0.0%) |  |
| Somewhat agree | 7 (0.5%) | 16 (3.0%) | 0 (0.0%) |  |
| Slightly disagree | 10 (0.8%) | 20 (3.7%) | 0 (0.0%) |  |
| Strongly disagree | 467 (36.1%) | 122 (22.8%) | 7 (38.9%) |  |
| No difference between men and women | 802 (62.1%) | 359 (67.2%) | 10 (55.6%) |  |
| *% Total of agreement** | *1.0%* | *6.2%* | *5.6%* |  |
| **A woman who works will never be completely a good mother and partner** | | | | |
| Completely agree | 3 (0.2%) | 1 (0.2%) | 0 (0.0%) | **< 0.001** |
| Strongly agree | 1 (0.1%) | 4 (0.7%) | 0 (0.0%) |  |
| Somewhat agree | 10 (0.8%) | 10 (1.9%) | 0 (0.0%) |  |
| Slightly disagree | 78 (6.0%) | 66 (12.4%) | 1 (5.6%) |  |
| Strongly disagree | 1200 (92.9%) | 453 (84.8%) | 17 (94.4%) |  |
| No difference between men and women | 0 (0.0%) | 0 (0.0%) | 0 (0.0%) |  |
| *% Total of agreement** | *1.1%* | *2.8%* | *0.0%* |  |
| **It is understandable that a man, more than a woman, can lose patience** | | | | |
| Completely agree | 0 (0.0%) | 5 (0.9%) | 0 (0.0%) | **< 0.001** |
| Strongly agree | 5 (0.4%) | 12 (2.2%) | 0 (0.0%) |  |
| Somewhat agree | 14 (1.1%) | 29 (5.4%) | 0 (0.0%) |  |
| Slightly disagree | 39 (3.0%) | 44 (8.2%) | 0 (0.0%) |  |
| Strongly disagree | 517 (40.0%) | 123 (23.0%) | 7 (38.9%) |  |
| No difference between men and women | 717 (55.5%) | 321 (60.1%) | 11 (61.1%) |  |
| *% Total of agreement** | *1.5%* | *8.5%* | *0.0%* |  |

*Note: The total percentage of agreement was calculated by summing the percentages of responses “completely agree”, “strongly agree”, and “somewhat agree”. This value is reported only for descriptive purposes and was not considered for the statistical analysis.

**Table 3Sc:** Stratification analyses conducted between each question composing the latent construct “Moral judgements” (total of 3 questions) and degree categories. Results containing p-values < 0.05 are highlighted in bold.

|  | **Elementary/Secondary school degrees (N=122)** | **High school degree (N=662)** | **University degree (N=737)** | **Post-University degree (N=323)** | **p-value** |
| --- | --- | --- | --- | --- | --- |
| **In a couple, female infidelity is more serious than male infidelity** | | | | | |
| Completely agree | 10 (8.2%) | 5 (0.8%) | 0 (0.0%) | 2 (0.6%) | **< 0.001** |
| Strongly agree | 5 (4.1%) | 1 (0.2%) | 1 (0.1%) | 0 (0.0%) |  |
| Somewhat agree | 7 (5.7%) | 9 (1.4%) | 3 (0.4%) | 4 (1.2%) |  |
| Slightly disagree | 2 (1.6%) | 9 (1.4%) | 16 (2.2%) | 3 (0.9%) |  |
| Strongly disagree | 28 (23.0%) | 228 (34.4%) | 243 (33.0%) | 97 (30.0%) |  |
| No difference between men and women | 70 (57.4%) | 410 (61.9%) | 474 (64.3%) | 217 (67.2%) |  |
| *% Total of agreement** | *18.0%* | *2.4%* | *0.5%* | *1.8%* |  |
| **A woman who works will never be completely a good mother and partner** | | | | | |
| Completely agree | 1 (0.8%) | 2 (0.3%) | 1 (0.1%) | 0 (0.0%) | **< 0.001** |
| Strongly agree | 3 (2.5%) | 1 (0.2%) | 1 (0.1%) | 0 (0.0%) |  |
| Somewhat agree | 4 (3.3%) | 11 (1.7%) | 4 (0.5%) | 1 (0.3%) |  |
| Slightly disagree | 19 (15.6%) | 54 (8.2%) | 52 (7.1%) | 20 (6.2%) |  |
| Strongly disagree | 95 (77.9%) | 594 (89.7%) | 679 (92.1%) | 302 (93.5%) |  |
| No difference between men and women | 0 (0.0%) | 0 (0.0%) | 0 (0.0%) | 0 (0.0%) |  |
| *% Total of agreement** | *6.6%* | *2.2%* | *0.7%* | *0.3%* |  |
| **It is understandable that a man, more than a woman, can lose patience** | | | | | |
| Completely agree | 5 (4.1%) | 0 (0.0%) | 0 (0.0%) | 0 (0.0%) | **< 0.001** |
| Strongly agree | 9 (7.4%) | 3 (0.5%) | 5 (0.7%) | 0 (0.0%) |  |
| Somewhat agree | 13 (10.7%) | 17 (2.6%) | 6 (0.8%) | 7 (2.2%) |  |
| Slightly disagree | 10 (8.2%) | 42 (6.3%) | 26 (3.5%) | 5 (1.5%) |  |
| Strongly disagree | 26 (21.3%) | 241 (36.4%) | 263 (35.7%) | 117 (36.2%) |  |
| No difference between men and women | 59 (48.4%) | 359 (54.2%) | 437 (59.3%) | 194 (60.1%) |  |
| *% Total of agreement** | *22.2%* | *3.1%* | *1.5%* | *2.2%* |  |

*Note: The total percentage of agreement was calculated by summing the percentages of responses “completely agree”, “strongly agree”, and “somewhat agree”. This value is reported only for descriptive purposes and was not considered for the statistical analysis.

**Table 4Sa:** Stratification analyses conducted between each question composing the cluster “Personality traits” (total of 15 questions) and age categories. Results containing p-values < 0.05 are highlighted in bold.

|  | **15-18 (N=74)** | **19-22 (N=223)** | **23-30 (N=347)** | **31-40 (N=336)** | **41-50 (N=304)** | **51-60 (N=296)** | **61-70 (N=214)** | **Over 70 (N=50)** | **p-value** |
| --- | --- | --- | --- | --- | --- | --- | --- | --- | --- |
| **Women are braver than men** | | | | | | | | | |
| Completely agree | 1 (1.4%) | 3 (1.3%) | 3 (0.9%) | 11 (3.3%) | 13 (4.3%) | 22 (7.4%) | 14 (6.5%) | 3 (6.0%) | **< 0.001** |
| Strongly agree | 0 (0.0%) | 8 (3.6%) | 17 (4.9%) | 18 (5.4%) | 28 (9.2%) | 31 (10.5%) | 28 (13.1%) | 5 (10.0%) |  |
| Somewhat agree | 6 (8.1%) | 24 (10.8%) | 38 (11.0%) | 61 (18.2%) | 77 (25.3%) | 67 (22.6%) | 62 (29.0%) | 17 (34.0%) |  |
| Slightly disagree | 17 (23.0%) | 37 (16.6%) | 52 (15.0%) | 49 (14.6%) | 43 (14.1%) | 37 (12.5%) | 20 (9.3%) | 9 (18.0%) |  |
| Strongly disagree | 23 (31.1%) | 36 (16.1%) | 32 (9.2%) | 21 (6.2%) | 23 (7.6%) | 16 (5.4%) | 13 (6.1%) | 6 (12.0%) |  |
| No difference between men and women | 27 (36.5%) | 115 (51.6%) | 205 (59.1%) | 176 (52.4%) | 120 (39.5%) | 123 (41.6%) | 77 (36.0%) | 10 (20.0%) |  |
| *% Total of agreement** | *9.5%* | *15.7%* | *16.8%* | *26.9%* | *38.8%* | *40.5%* | *48.6%* | *50.0%* |  |
| **Women are more jealous than men** | | | | | | | | | |
| Completely agree | 8 (10.8%) | 2 (0.9%) | 3 (0.9%) | 2 (0.6%) | 0 (0.0%) | 1 (0.3%) | 1 (0.5%) | 0 (0.0%) | **< 0.001** |
| Strongly agree | 11 (14.9%) | 7 (3.1%) | 6 (1.7%) | 10 (3.0%) | 5 (1.6%) | 3 (1.0%) | 5 (2.3%) | 1 (2.0%) |  |
| Somewhat agree | 9 (12.2%) | 27 (12.1%) | 41 (11.8%) | 28 (8.3%) | 22 (7.2%) | 13 (4.4%) | 14 (6.5%) | 4 (8.0%) |  |
| Slightly disagree | 11 (14.9%) | 31 (13.9%) | 43 (12.4%) | 41 (12.2%) | 36 (11.8%) | 44 (14.9%) | 23 (10.7%) | 7 (14.0%) |  |
| Strongly disagree | 16 (21.6%) | 44 (19.7%) | 71 (20.5%) | 54 (16.1%) | 93 (30.6%) | 81 (27.4%) | 65 (30.4%) | 18 (36.0%) |  |
| No difference between men and women | 19 (25.7%) | 112 (50.2%) | 183 (52.7%) | 201 (59.8%) | 148 (48.7%) | 154 (52.0%) | 106 (49.5%) | 20 (40.0%) |  |
| *% Total of agreement** | *37.9%* | *16.1%* | *14.4%* | *11.9%* | *8.8%* | *5.7%* | *9.3%* | *10.0%* |  |
| **Women are more aggressive than men** | | | | | | | | | |
| Completely agree | 4 (5.4%) | 4 (1.8%) | 3 (0.9%) | 2 (0.6%) | 2 (0.7%) | 1 (0.3%) | 1 (0.5%) | 0 (0.0%) | **< 0.001** |
| Strongly agree | 4 (5.4%) | 1 (0.4%) | 2 (0.6%) | 3 (0.9%) | 1 (0.3%) | 5 (1.7%) | 1 (0.5%) | 0 (0.0%) |  |
| Somewhat agree | 8 (10.8%) | 8 (3.6%) | 12 (3.5%) | 7 (2.1%) | 18 (5.9%) | 19 (6.4%) | 16 (7.5%) | 5 (10.0%) |  |
| Slightly disagree | 12 (16.2%) | 35 (15.7%) | 55 (15.9%) | 56 (16.7%) | 45 (14.8%) | 49 (16.6%) | 44 (20.6%) | 9 (18.0%) |  |
| Strongly disagree | 28 (37.8%) | 71 (31.8%) | 110 (31.7%) | 96 (28.6%) | 102 (33.6%) | 98 (33.1%) | 67 (31.3%) | 17 (34.0%) |  |
| No difference between men and women | 18 (24.3%) | 104 (46.6%) | 165 (47.6%) | 172 (51.2%) | 136 (44.7%) | 124 (41.9%) | 85 (39.7%) | 19 (38.0%) |  |
| *% Total of agreement** | *21.6%* | *5.8%* | *5.0%* | *3.6%* | *6.9%* | *8.4%* | *8.5%* | *10.0%* |  |
| **Women are kinder than men** | | | | | | | | | |
| Completely agree | 1 (1.4%) | 2 (0.9%) | 2 (0.6%) | 1 (0.3%) | 4 (1.3%) | 3 (1.0%) | 4 (1.9%) | 2 (4.0%) | **< 0.001** |
| Strongly agree | 2 (2.7%) | 4 (1.8%) | 9 (2.6%) | 8 (2.4%) | 10 (3.3%) | 7 (2.4%) | 8 (3.7%) | 5 (10.0%) |  |
| Somewhat agree | 8 (10.8%) | 17 (7.6%) | 28 (8.1%) | 39 (11.6%) | 23 (7.6%) | 40 (13.5%) | 50 (23.4%) | 13 (26.0%) |  |
| Slightly disagree | 14 (18.9%) | 26 (11.7%) | 54 (15.6%) | 44 (13.1%) | 52 (17.1%) | 38 (12.8%) | 29 (13.6%) | 8 (16.0%) |  |
| Strongly disagree | 10 (13.5%) | 36 (16.1%) | 37 (10.7%) | 38 (11.3%) | 47 (15.5%) | 46 (15.5%) | 26 (12.1%) | 6 (12.0%) |  |
| No difference between men and women | 39 (52.7%) | 138 (61.9%) | 217 (62.5%) | 206 (61.3%) | 168 (55.3%) | 162 (54.7%) | 97 (45.3%) | 16 (32.0%) |  |
| *% Total of agreement** | *14.9%* | *10.3%* | *11.3%* | *14.3%* | *12.2%* | *16.9%* | *29.0%* | *40.0%* |  |
| **For men, more than for women, it is very important to have success in their job** | | | | | | | | | |
| Completely agree | 6 (8.1%) | 1 (0.4%) | 3 (0.9%) | 2 (0.6%) | 7 (2.3%) | 7 (2.4%) | 1 (0.5%) | 2 (4.0%) | **< 0.001** |
| Strongly agree | 4 (5.4%) | 7 (3.1%) | 7 (2.0%) | 17 (5.1%) | 21 (6.9%) | 7 (2.4%) | 11 (5.1%) | 4 (8.0%) |  |
| Somewhat agree | 13 (17.6%) | 11 (4.9%) | 18 (5.2%) | 43 (12.8%) | 59 (19.4%) | 60 (20.3%) | 49 (22.9%) | 8 (16.0%) |  |
| Slightly disagree | 10 (13.5%) | 23 (10.3%) | 34 (9.8%) | 38 (11.3%) | 37 (12.2%) | 33 (11.1%) | 26 (12.1%) | 11 (22.0%) |  |
| Strongly disagree | 11 (14.9%) | 49 (22.0%) | 83 (23.9%) | 65 (19.3%) | 58 (19.1%) | 55 (18.6%) | 35 (16.4%) | 9 (18.0%) |  |
| No difference between men and women | 30 (40.5%) | 132 (59.2%) | 202 (58.2%) | 171 (50.9%) | 122 (40.1%) | 134 (45.3%) | 92 (43.0%) | 16 (32.0%) |  |
| *% Total of agreement** | *31.1%* | *8.4%* | *8.1%* | *18.5%* | *28.6%* | *25.1%* | *28.5%* | *28.0%* |  |
| **Women are more generous than men** | | | | | | | | | |
| Completely agree | 1 (1.4%) | 2 (0.9%) | 1 (0.3%) | 0 (0.0%) | 2 (0.7%) | 1 (0.3%) | 4 (1.9%) | 0 (0.0%) | **< 0.001** |
| Strongly agree | 2 (2.7%) | 1 (0.4%) | 1 (0.3%) | 1 (0.3%) | 4 (1.3%) | 5 (1.7%) | 7 (3.3%) | 4 (8.0%) |  |
| Somewhat agree | 9 (12.2%) | 11 (4.9%) | 13 (3.7%) | 21 (6.2%) | 14 (4.6%) | 29 (9.8%) | 39 (18.2%) | 8 (16.0%) |  |
| Slightly disagree | 12 (16.2%) | 26 (11.7%) | 30 (8.6%) | 33 (9.8%) | 36 (11.8%) | 24 (8.1%) | 19 (8.9%) | 9 (18.0%) |  |
| Strongly disagree | 17 (23.0%) | 37 (16.6%) | 47 (13.5%) | 41 (12.2%) | 56 (18.4%) | 44 (14.9%) | 31 (14.5%) | 8 (16.0%) |  |
| No difference between men and women | 33 (44.6%) | 146 (65.5%) | 255 (73.5%) | 240 (71.4%) | 192 (63.2%) | 193 (65.2%) | 114 (53.3%) | 21 (42.0%) |  |
| *% Total of agreement** | *16.3%* | *6.2%* | *4.3%* | *6.5%* | *6.6%* | *11.8%* | *23.4%* | *24.0%* |  |
| **Women are nicer than men** | | | | | | | | | |
| Completely agree | 0 (0.0%) | 1 (0.4%) | 3 (0.9%) | 0 (0.0%) | 2 (0.7%) | 2 (0.7%) | 3 (1.4%) | 3 (6.0%) | **< 0.001** |
| Strongly agree | 1 (1.4%) | 0 (0.0%) | 3 (0.9%) | 2 (0.6%) | 2 (0.7%) | 3 (1.0%) | 5 (2.3%) | 0 (0.0%) |  |
| Somewhat agree | 4 (5.4%) | 6 (2.7%) | 8 (2.3%) | 7 (2.1%) | 7 (2.3%) | 9 (3.0%) | 11 (5.1%) | 4 (8.0%) |  |
| Slightly disagree | 17 (23.0%) | 26 (11.7%) | 21 (6.1%) | 22 (6.5%) | 22 (7.2%) | 21 (7.1%) | 16 (7.5%) | 6 (12.0%) |  |
| Strongly disagree | 15 (20.3%) | 34 (15.2%) | 49 (14.1%) | 47 (14.0%) | 48 (15.8%) | 57 (19.3%) | 41 (19.2%) | 10 (20.0%) |  |
| No difference between men and women | 37 (50.0%) | 156 (70.0%) | 263 (75.8%) | 258 (76.8%) | 223 (73.4%) | 204 (68.9%) | 138 (64.5%) | 27 (54.0%) |  |
| *% Total of agreement** | *6.8%* | *3.1%* | *4.1%* | *2.7%* | *3.7%* | *4.7%* | *8.8%* | *14.0%* |  |
| **Women are more competitive than men** | | | | | | | | | |
| Completely agree | 0 (0.0%) | 2 (0.9%) | 6 (1.7%) | 11 (3.3%) | 3 (1.0%) | 4 (1.4%) | 3 (1.4%) | 1 (2.0%) | 0.460 |
| Strongly agree | 3 (4.1%) | 7 (3.1%) | 9 (2.6%) | 12 (3.6%) | 11 (3.6%) | 10 (3.4%) | 4 (1.9%) | 1 (2.0%) |  |
| Somewhat agree | 11 (14.9%) | 19 (8.5%) | 48 (13.8%) | 37 (11.0%) | 35 (11.5%) | 31 (10.5%) | 26 (12.1%) | 6 (12.0%) |  |
| Slightly disagree | 12 (16.2%) | 25 (11.2%) | 27 (7.8%) | 34 (10.1%) | 33 (10.9%) | 31 (10.5%) | 32 (15.0%) | 10 (20.0%) |  |
| Strongly disagree | 19 (25.7%) | 36 (16.1%) | 39 (11.2%) | 37 (11.0%) | 52 (17.1%) | 51 (17.2%) | 29 (13.6%) | 9 (18.0%) |  |
| No difference between men and women | 29 (39.2%) | 134 (60.1%) | 218 (62.8%) | 205 (61.0%) | 170 (55.9%) | 169 (57.1%) | 120 (56.1%) | 23 (46.0%) |  |
| *% Total of agreement** | *19.0%* | *12.5%* | *18.1%* | *17.9%* | *16.1%* | *15.3%* | *15.4%* | *16.0%* |  |
| **Women are more selfish than men** | | | | | | | | | |
| Completely agree | 6 (8.1%) | 0 (0.0%) | 1 (0.3%) | 1 (0.3%) | 0 (0.0%) | 1 (0.3%) | 1 (0.5%) | 0 (0.0%) | **< 0.001** |
| Strongly agree | 6 (8.1%) | 2 (0.9%) | 4 (1.2%) | 1 (0.3%) | 1 (0.3%) | 1 (0.3%) | 0 (0.0%) | 1 (2.0%) |  |
| Somewhat agree | 10 (13.5%) | 11 (4.9%) | 10 (2.9%) | 5 (1.5%) | 7 (2.3%) | 10 (3.4%) | 7 (3.3%) | 0 (0.0%) |  |
| Slightly disagree | 11 (14.9%) | 18 (8.1%) | 27 (7.8%) | 25 (7.4%) | 27 (8.9%) | 19 (6.4%) | 25 (11.7%) | 5 (10.0%) |  |
| Strongly disagree | 12 (16.2%) | 47 (21.1%) | 75 (21.6%) | 68 (20.2%) | 79 (26.0%) | 85 (28.7%) | 69 (32.2%) | 21 (42.0%) |  |
| No difference between men and women | 29 (39.2%) | 145 (65.0%) | 230 (66.3%) | 236 (70.2%) | 190 (62.5%) | 180 (60.8%) | 112 (52.3%) | 23 (46.0%) |  |
| *% Total of agreement** | *29.7%* | *5.8%* | *4.4%* | *2.1%* | *2.6%* | *4.0%* | *3.8%* | *2.0%* |  |
| **Women are more sensitive than men** | | | | | | | | | |
| Completely agree | 9 (12.2%) | 5 (2.2%) | 7 (2.0%) | 5 (1.5%) | 4 (1.3%) | 9 (3.0%) | 9 (4.2%) | 3 (6.0%) | **< 0.001** |
| Strongly agree | 19 (25.7%) | 12 (5.4%) | 15 (4.3%) | 14 (4.2%) | 21 (6.9%) | 14 (4.7%) | 18 (8.4%) | 2 (4.0%) |  |
| Somewhat agree | 11 (14.9%) | 43 (19.3%) | 75 (21.6%) | 73 (21.7%) | 70 (23.0%) | 78 (26.4%) | 75 (35.0%) | 26 (52.0%) |  |
| Slightly disagree | 4 (5.4%) | 29 (13.0%) | 48 (13.8%) | 37 (11.0%) | 31 (10.2%) | 40 (13.5%) | 23 (10.7%) | 4 (8.0%) |  |
| Strongly disagree | 8 (10.8%) | 24 (10.8%) | 29 (8.4%) | 33 (9.8%) | 34 (11.2%) | 34 (11.5%) | 17 (7.9%) | 3 (6.0%) |  |
| No difference between men and women | 23 (31.1%) | 110 (49.3%) | 173 (49.9%) | 174 (51.8%) | 144 (47.4%) | 121 (40.9%) | 72 (33.6%) | 12 (24.0%) |  |
| *% Total of agreement** | *52.8%* | *26.9%* | *27.9%* | *27.4%* | *31.2%* | *34.1%* | *47.6%* | *62.0%* |  |
| **Women are more determined than men** | | | | | | | | | |
| Completely agree | 0 (0.0%) | 1 (0.4%) | 3 (0.9%) | 3 (0.9%) | 6 (2.0%) | 10 (3.4%) | 7 (3.3%) | 1 (2.0%) | **< 0.001** |
| Strongly agree | 2 (2.7%) | 8 (3.6%) | 6 (1.7%) | 14 (4.2%) | 17 (5.6%) | 18 (6.1%) | 12 (5.6%) | 7 (14.0%) |  |
| Somewhat agree | 10 (13.5%) | 21 (9.4%) | 38 (11.0%) | 43 (12.8%) | 60 (19.7%) | 59 (19.9%) | 65 (30.4%) | 20 (40.0%) |  |
| Slightly disagree | 16 (21.6%) | 11 (4.9%) | 17 (4.9%) | 23 (6.8%) | 20 (6.6%) | 20 (6.8%) | 17 (7.9%) | 3 (6.0%) |  |
| Strongly disagree | 15 (20.3%) | 29 (13.0%) | 30 (8.6%) | 29 (8.6%) | 24 (7.9%) | 22 (7.4%) | 12 (5.6%) | 2 (4.0%) |  |
| No difference between men and women | 31 (41.9%) | 153 (68.6%) | 253 (72.9%) | 224 (66.7%) | 177 (58.2%) | 167 (56.4%) | 101 (47.2%) | 17 (34.0%) |  |
| *% Total of agreement** | *16.2%* | *13.4%* | *13.6%* | *17.9%* | *27.3%* | *29.4%* | *39.3%* | *56.0%* |  |
| **Women are more disorganized than men** | | | | | | | | | |
| Completely agree | 3 (4.1%) | 2 (0.9%) | 2 (0.6%) | 2 (0.6%) | 0 (0.0%) | 1 (0.3%) | 0 (0.0%) | 0 (0.0%) | **< 0.001** |
| Strongly agree | 1 (1.4%) | 0 (0.0%) | 0 (0.0%) | 0 (0.0%) | 0 (0.0%) | 1 (0.3%) | 1 (0.5%) | 0 (0.0%) |  |
| Somewhat agree | 7 (9.5%) | 5 (2.2%) | 6 (1.7%) | 6 (1.8%) | 7 (2.3%) | 7 (2.4%) | 5 (2.3%) | 3 (6.0%) |  |
| Slightly disagree | 12 (16.2%) | 13 (5.8%) | 16 (4.6%) | 33 (9.8%) | 18 (5.9%) | 15 (5.1%) | 12 (5.6%) | 5 (10.0%) |  |
| Strongly disagree | 16 (21.6%) | 80 (35.9%) | 128 (36.9%) | 109 (32.4%) | 138 (45.4%) | 143 (48.3%) | 126 (58.9%) | 28 (56.0%) |  |
| No difference between men and women | 35 (47.3%) | 123 (55.2%) | 195 (56.2%) | 186 (55.4%) | 141 (46.4%) | 129 (43.6%) | 70 (32.7%) | 14 (28.0%) |  |
| *% Total of agreement** | *15.0%* | *3.1%* | *2.3%* | *2.4%* | *2.3%* | *3.0%* | *2.8%* | *6.0%* |  |
| **Women are more stubborn than men** | | | | | | | | | |
| Completely agree | 6 (8.1%) | 1 (0.4%) | 6 (1.7%) | 5 (1.5%) | 6 (2.0%) | 7 (2.4%) | 4 (1.9%) | 1 (2.0%) | **< 0.001** |
| Strongly agree | 11 (14.9%) | 10 (4.5%) | 6 (1.7%) | 14 (4.2%) | 7 (2.3%) | 8 (2.7%) | 9 (4.2%) | 1 (2.0%) |  |
| Somewhat agree | 12 (16.2%) | 21 (9.4%) | 32 (9.2%) | 31 (9.2%) | 32 (10.5%) | 39 (13.2%) | 40 (18.7%) | 14 (28.0%) |  |
| Slightly disagree | 7 (9.5%) | 18 (8.1%) | 20 (5.8%) | 18 (5.4%) | 15 (4.9%) | 22 (7.4%) | 12 (5.6%) | 4 (8.0%) |  |
| Strongly disagree | 8 (10.8%) | 33 (14.8%) | 29 (8.4%) | 32 (9.5%) | 54 (17.8%) | 39 (13.2%) | 31 (14.5%) | 6 (12.0%) |  |
| No difference between men and women | 30 (40.5%) | 140 (62.8%) | 254 (73.2%) | 236 (70.2%) | 190 (62.5%) | 181 (61.1%) | 118 (55.1%) | 24 (48.0%) |  |
| *% Total of agreement** | *39.2%* | *14.3%* | *12.6%* | *14.9%* | *14.8%* | *18.3%* | *24.8%* | *32.0%* |  |
| **Men are more aggressive than women** | | | | | | | | | |
| Completely agree | 9 (12.2%) | 4 (1.8%) | 6 (1.7%) | 5 (1.5%) | 4 (1.3%) | 4 (1.4%) | 4 (1.9%) | 1 (2.0%) | **< 0.001** |
| Strongly agree | 3 (4.1%) | 13 (5.8%) | 13 (3.7%) | 11 (3.3%) | 15 (4.9%) | 10 (3.4%) | 10 (4.7%) | 1 (2.0%) |  |
| Somewhat agree | 11 (14.9%) | 32 (14.3%) | 57 (16.4%) | 57 (17.0%) | 44 (14.5%) | 47 (15.9%) | 59 (27.6%) | 16 (32.0%) |  |
| Slightly disagree | 14 (18.9%) | 27 (12.1%) | 45 (13.0%) | 33 (9.8%) | 42 (13.8%) | 38 (12.8%) | 25 (11.7%) | 10 (20.0%) |  |
| Strongly disagree | 10 (13.5%) | 28 (12.6%) | 41 (11.8%) | 37 (11.0%) | 36 (11.8%) | 33 (11.1%) | 19 (8.9%) | 7 (14.0%) |  |
| No difference between men and women | 27 (36.5%) | 119 (53.4%) | 185 (53.3%) | 193 (57.4%) | 163 (53.6%) | 164 (55.4%) | 97 (45.3%) | 15 (30.0%) |  |
| *% Total of agreement** | *31.2%* | *21.9%* | *21.8%* | *21.8%* | *20.7%* | *20.7%* | *34.2%* | *36.0%* |  |
| **Women are more cunning than men** | | | | | | | | | |
| Completely agree | 4 (5.4%) | 5 (2.2%) | 11 (3.2%) | 5 (1.5%) | 12 (3.9%) | 14 (4.7%) | 10 (4.7%) | 6 (12.0%) | **< 0.001** |
| Strongly agree | 5 (6.8%) | 10 (4.5%) | 15 (4.3%) | 17 (5.1%) | 8 (2.6%) | 7 (2.4%) | 9 (4.2%) | 4 (8.0%) |  |
| Somewhat agree | 7 (9.5%) | 31 (13.9%) | 47 (13.5%) | 44 (13.1%) | 32 (10.5%) | 39 (13.2%) | 44 (20.6%) | 8 (16.0%) |  |
| Slightly disagree | 9 (12.2%) | 17 (7.6%) | 28 (8.1%) | 20 (6.0%) | 26 (8.6%) | 17 (5.7%) | 20 (9.3%) | 9 (18.0%) |  |
| Strongly disagree | 13 (17.6%) | 24 (10.8%) | 25 (7.2%) | 22 (6.5%) | 28 (9.2%) | 36 (12.2%) | 23 (10.7%) | 7 (14.0%) |  |
| No difference between men and women | 36 (48.6%) | 136 (61.0%) | 221 (63.7%) | 228 (67.9%) | 198 (65.1%) | 183 (61.8%) | 108 (50.5%) | 16 (32.0%) |  |
| *% Total of agreement** | *21.7%* | *20.6%* | *21.0%* | *19.7%* | *17.0%* | *20.3%* | *29.5%* | *36.0%* |  |

*Note: The total percentage of agreement was calculated by summing the percentages of responses “completely agree”, “strongly agree”, and “somewhat agree”. This value is reported only for descriptive purposes and was not considered for the statistical analysis.

**Table 4Sb:** Stratification analyses conducted between each question composing the latent construct “Personality traits” (total of 15 questions) and gender categories. Results containing p-values < 0.05 are highlighted in bold.

|  | **Female (N=1292)** | **Male (N=534)** | **Other/Do not want to answer (N=18)** | **p-value** |
| --- | --- | --- | --- | --- |
| **Women are braver than men** | | | | |
| Completely agree | 62 (4.8%) | 8 (1.5%) | 0 (0.0%) | **< 0.001** |
| Strongly agree | 110 (8.5%) | 24 (4.5%) | 1 (5.6%) |  |
| Somewhat agree | 277 (21.4%) | 71 (13.3%) | 4 (22.2%) |  |
| Slightly disagree | 159 (12.3%) | 103 (19.3%) | 2 (11.1%) |  |
| Strongly disagree | 97 (7.5%) | 71 (13.3%) | 2 (11.1%) |  |
| No difference between men and women | 587 (45.4%) | 257 (48.1%) | 9 (50.0%) |  |
| *% Total of agreement** | *34.7%* | *19.3%* | *27.8%* |  |
| **Women are more jealous than men** | | | | |
| Completely agree | 6 (0.5%) | 11 (2.1%) | 0 (0.0%) | **< 0.001** |
| Strongly agree | 16 (1.2%) | 32 (6.0%) | 0 (0.0%) |  |
| Somewhat agree | 89 (6.9%) | 69 (12.9%) | 0 (0.0%) |  |
| Slightly disagree | 152 (11.8%) | 82 (15.4%) | 2 (11.1%) |  |
| Strongly disagree | 351 (27.2%) | 85 (15.9%) | 6 (33.3%) |  |
| No difference between men and women | 678 (52.5%) | 255 (47.8%) | 10 (55.6%) |  |
| *% Total of agreement** | *8.6%* | *20.7%* | *0.0%* |  |
| **Women are more aggressive than men** | | | | |
| Completely agree | 4 (0.3%) | 13 (2.4%) | 0 (0.0%) | **< 0.001** |
| Strongly agree | 7 (0.5%) | 9 (1.7%) | 1 (5.6%) |  |
| Somewhat agree | 48 (3.7%) | 44 (8.2%) | 1 (5.6%) |  |
| Slightly disagree | 201 (15.6%) | 104 (19.5%) | 0 (0.0%) |  |
| Strongly disagree | 437 (33.8%) | 145 (27.2%) | 7 (38.9%) |  |
| No difference between men and women | 595 (46.1%) | 219 (41.0%) | 9 (50.0%) |  |
| *% Total of agreement** | *4.5%* | *12.3%* | *11.2%* |  |
| **Women are kinder than men** | | | | |
| Completely agree | 13 (1.0%) | 6 (1.1%) | 0 (0.0%) | **0.046** |
| Strongly agree | 27 (2.1%) | 23 (4.3%) | 3 (16.7%) |  |
| Somewhat agree | 142 (11.0%) | 74 (13.9%) | 2 (11.1%) |  |
| Slightly disagree | 185 (14.3%) | 79 (14.8%) | 1 (5.6%) |  |
| Strongly disagree | 187 (14.5%) | 58 (10.9%) | 1 (5.6%) |  |
| No difference between men and women | 738 (57.1%) | 294 (55.1%) | 11 (61.1%) |  |
| *% Total of agreement** | *14.1%* | *19.3%* | *27.8%* |  |
| **For men, more than for women, it is very important to have success in their job** | | | | |
| Completely agree | 12 (0.9%) | 16 (3.0%) | 1 (5.6%) | 0.103 |
| Strongly agree | 45 (3.5%) | 32 (6.0%) | 1 (5.6%) |  |
| Somewhat agree | 183 (14.2%) | 76 (14.2%) | 2 (11.1%) |  |
| Slightly disagree | 142 (11.0%) | 70 (13.1%) | 0 (0.0%) |  |
| Strongly disagree | 295 (22.8%) | 67 (12.5%) | 3 (16.7%) |  |
| No difference between men and women | 615 (47.6%) | 273 (51.1%) | 11 (61.1%) |  |
| *% Total of agreement** | *18.6%* | *23.2%* | *22.3%* |  |
| **Women are more generous than men** | | | | |
| Completely agree | 8 (0.6%) | 3 (0.6%) | 0 (0.0%) | 0.464 |
| Strongly agree | 18 (1.4%) | 6 (1.1%) | 1 (5.6%) |  |
| Somewhat agree | 104 (8.0%) | 39 (7.3%) | 1 (5.6%) |  |
| Slightly disagree | 111 (8.6%) | 76 (14.2%) | 2 (11.1%) |  |
| Strongly disagree | 201 (15.6%) | 77 (14.4%) | 3 (16.7%) |  |
| No difference between men and women | 850 (65.8%) | 333 (62.4%) | 11 (61.1%) |  |
| *% Total of agreement** | *10.0%* | *9.0%* | *11.2%* |  |
| **Women are nicer than men** | | | | |
| Completely agree | 11 (0.9%) | 2 (0.4%) | 1 (5.6%) | **< 0.001** |
| Strongly agree | 6 (0.5%) | 10 (1.9%) | 0 (0.0%) |  |
| Somewhat agree | 26 (2.0%) | 28 (5.2%) | 2 (11.1%) |  |
| Slightly disagree | 81 (6.3%) | 70 (13.1%) | 0 (0.0%) |  |
| Strongly disagree | 218 (16.9%) | 81 (15.2%) | 2 (11.1%) |  |
| No difference between men and women | 950 (73.5%) | 343 (64.2%) | 13 (72.2%) |  |
| *% Total of agreement** | *3.4%* | *7.5%* | *16.7%* |  |
| **Women are more competitive than men** | | | | |
| Completely agree | 22 (1.7%) | 8 (1.5%) | 0 (0.0%) | **< 0.001** |
| Strongly agree | 25 (1.9%) | 32 (6.0%) | 0 (0.0%) |  |
| Somewhat agree | 140 (10.8%) | 73 (13.7%) | 0 (0.0%) |  |
| Slightly disagree | 134 (10.4%) | 67 (12.5%) | 3 (16.7%) |  |
| Strongly disagree | 198 (15.3%) | 72 (13.5%) | 2 (11.1%) |  |
| No difference between men and women | 773 (59.8%) | 282 (52.8%) | 13 (72.2%) |  |
| *% Total of agreement** | *14.4%* | *21.2%* | *0.0%* |  |
| **Women are more selfish than men** | | | | |
| Completely agree | 2 (0.2%) | 8 (1.5%) | 0 (0.0%) | **< 0.001** |
| Strongly agree | 3 (0.2%) | 12 (2.2%) | 1 (5.6%) |  |
| Somewhat agree | 21 (1.6%) | 39 (7.3%) | 0 (0.0%) |  |
| Slightly disagree | 93 (7.2%) | 63 (11.8%) | 1 (5.6%) |  |
| Strongly disagree | 374 (28.9%) | 77 (14.4%) | 5 (27.8%) |  |
| No difference between men and women | 799 (61.8%) | 335 (62.7%) | 11 (61.1%) |  |
| *% Total of agreement** | *2.0%* | *11.0%* | *5.6%* |  |
| **Women are more sensitive than men** | | | | |
| Completely agree | 26 (2.0%) | 25 (4.7%) | 0 (0.0%) | **0.001** |
| Strongly agree | 62 (4.8%) | 49 (9.2%) | 4 (22.2%) |  |
| Somewhat agree | 315 (24.4%) | 132 (24.7%) | 4 (22.2%) |  |
| Slightly disagree | 155 (12.0%) | 61 (11.4%) | 0 (0.0%) |  |
| Strongly disagree | 136 (10.5%) | 45 (8.4%) | 1 (5.6%) |  |
| No difference between men and women | 598 (46.3%) | 222 (41.6%) | 9 (50.0%) |  |
| *% Total of agreement** | *31.2%* | *38.6%* | *44.4%* |  |
| **Women are more determined than men** | | | | |
| Completely agree | 23 (1.8%) | 8 (1.5%) | 0 (0.0%) | 0.131 |
| Strongly agree | 64 (5.0%) | 19 (3.6%) | 1 (5.6%) |  |
| Somewhat agree | 205 (15.9%) | 109 (20.4%) | 2 (11.1%) |  |
| Slightly disagree | 81 (6.3%) | 46 (8.6%) | 0 (0.0%) |  |
| Strongly disagree | 112 (8.7%) | 50 (9.4%) | 1 (5.6%) |  |
| No difference between men and women | 807 (62.5%) | 302 (56.6%) | 14 (77.8%) |  |
| *% Total of agreement** | *22.7%* | *25.5%* | *16.7%* |  |
| **Women are more disorganized than men** | | | | |
| Completely agree | 1 (0.1%) | 9 (1.7%) | 0 (0.0%) | **0.003** |
| Strongly agree | 2 (0.2%) | 1 (0.2%) | 0 (0.0%) |  |
| Somewhat agree | 20 (1.5%) | 25 (4.7%) | 1 (5.6%) |  |
| Slightly disagree | 60 (4.6%) | 64 (12.0%) | 0 (0.0%) |  |
| Strongly disagree | 610 (47.2%) | 152 (28.5%) | 6 (33.3%) |  |
| No difference between men and women | 599 (46.4%) | 283 (53.0%) | 11 (61.1%) |  |
| *% Total of agreement** | *1.8%* | *6.6%* | *5.6%* |  |
| **Women are more stubborn than men** | | | | |
| Completely agree | 18 (1.4%) | 18 (3.4%) | 0 (0.0%) | **< 0.001** |
| Strongly agree | 32 (2.5%) | 33 (6.2%) | 1 (5.6%) |  |
| Somewhat agree | 140 (10.8%) | 79 (14.8%) | 2 (11.1%) |  |
| Slightly disagree | 76 (5.9%) | 40 (7.5%) | 0 (0.0%) |  |
| Strongly disagree | 180 (13.9%) | 50 (9.4%) | 2 (11.1%) |  |
| No difference between men and women | 846 (65.5%) | 314 (58.8%) | 13 (72.2%) |  |
| *% Total of agreement** | *14.7%* | *24.4%* | *16.7%* |  |
| **Men are more aggressive than women** | | | | |
| Completely agree | 15 (1.2%) | 22 (4.1%) | 0 (0.0%) | **< 0.001** |
| Strongly agree | 43 (3.3%) | 28 (5.2%) | 5 (27.8%) |  |
| Somewhat agree | 215 (16.6%) | 106 (19.9%) | 2 (11.1%) |  |
| Slightly disagree | 171 (13.2%) | 62 (11.6%) | 1 (5.6%) |  |
| Strongly disagree | 152 (11.8%) | 58 (10.9%) | 1 (5.6%) |  |
| No difference between men and women | 696 (53.9%) | 258 (48.3%) | 9 (50.0%) |  |
| *% Total of agreement** | *21.1%* | *29.2%* | *38.9%* |  |
| **Women are more cunning than men** | | | | |
| Completely agree | 33 (2.6%) | 34 (6.4%) | 0 (0.0%) | **< 0.001** |
| Strongly agree | 42 (3.3%) | 32 (6.0%) | 1 (5.6%) |  |
| Somewhat agree | 183 (14.2%) | 69 (12.9%) | 0 (0.0%) |  |
| Slightly disagree | 95 (7.4%) | 48 (9.0%) | 3 (16.7%) |  |
| Strongly disagree | 130 (10.1%) | 46 (8.6%) | 2 (11.1%) |  |
| No difference between men and women | 809 (62.6%) | 305 (57.1%) | 12 (66.7%) |  |
| *% Total of agreement** | *20.1%* | *25.3%* | *5.6%* |  |

*Note: The total percentage of agreement was calculated by summing the percentages of responses “completely agree”, “strongly agree”, and “somewhat agree”. This value is reported only for descriptive purposes and was not considered for the statistical analysis.

**Table 4Sc:** Stratification analyses conducted between each question composing the latent construct “Personality traits” (total of 15 questions) and degree categories. Results containing p-values < 0.05 are highlighted in bold.

|  | **Elementary/Secondary school degrees**  **(N=122)** | **High school degree (N=662)** | **University degree (N=737)** | **Post-University degree (N=323)** | **p-value** |
| --- | --- | --- | --- | --- | --- |
| **Women are braver than men** | | | | | |
| Completely agree | 6 (4.9%) | 27 (4.1%) | 31 (4.2%) | 6 (1.9%) | 0.445 |
| Strongly agree | 12 (9.8%) | 48 (7.3%) | 50 (6.8%) | 25 (7.7%) |  |
| Somewhat agree | 20 (16.4%) | 126 (19.0%) | 142 (19.3%) | 64 (19.8%) |  |
| Slightly disagree | 20 (16.4%) | 95 (14.4%) | 105 (14.2%) | 44 (13.6%) |  |
| Strongly disagree | 20 (16.4%) | 64 (9.7%) | 58 (7.9%) | 28 (8.7%) |  |
| No difference between men and women | 44 (36.1%) | 302 (45.6%) | 351 (47.6%) | 156 (48.3%) |  |
| *% Total of agreement** | *31.1%* | *30.4%* | *30.3%* | *29.4%* |  |
| **Women are more jealous than men** | | | | | |
| Completely agree | 7 (5.7%) | 4 (0.6%) | 3 (0.4%) | 3 (0.9%) | **< 0.001** |
| Strongly agree | 12 (9.8%) | 19 (2.9%) | 10 (1.4%) | 7 (2.2%) |  |
| Somewhat agree | 12 (9.8%) | 67 (10.1%) | 55 (7.5%) | 24 (7.4%) |  |
| Slightly disagree | 18 (14.8%) | 75 (11.3%) | 105 (14.2%) | 38 (11.8%) |  |
| Strongly disagree | 32 (26.2%) | 173 (26.1%) | 167 (22.7%) | 70 (21.7%) |  |
| No difference between men and women | 41 (33.6%) | 324 (48.9%) | 397 (53.9%) | 181 (56.0%) |  |
| *% Total of agreement** | *25.3%* | *13.6%* | *9.3%* | *10.5%* |  |
| **Women are more aggressive than men** | | | | | |
| Completely agree | 4 (3.3%) | 8 (1.2%) | 4 (0.5%) | 1 (0.3%) | **0.001** |
| Strongly agree | 3 (2.5%) | 8 (1.2%) | 4 (0.5%) | 2 (0.6%) |  |
| Somewhat agree | 9 (7.4%) | 40 (6.0%) | 31 (4.2%) | 13 (4.0%) |  |
| Slightly disagree | 21 (17.2%) | 101 (15.3%) | 118 (16.0%) | 65 (20.1%) |  |
| Strongly disagree | 44 (36.1%) | 216 (32.6%) | 232 (31.5%) | 97 (30.0%) |  |
| No difference between men and women | 41 (33.6%) | 289 (43.7%) | 348 (47.2%) | 145 (44.9%) |  |
| *% Total of agreement** | *13.2%* | *8.4%* | *5.2%* | *4.9%* |  |
| **Women are kinder than men** | | | | | |
| Completely agree | 2 (1.6%) | 7 (1.1%) | 7 (0.9%) | 3 (0.9%) | 0.682 |
| Strongly agree | 4 (3.3%) | 17 (2.6%) | 19 (2.6%) | 13 (4.0%) |  |
| Somewhat agree | 15 (12.3%) | 85 (12.8%) | 73 (9.9%) | 45 (13.9%) |  |
| Slightly disagree | 17 (13.9%) | 86 (13.0%) | 117 (15.9%) | 45 (13.9%) |  |
| Strongly disagree | 15 (12.3%) | 100 (15.1%) | 101 (13.7%) | 30 (9.3%) |  |
| No difference between men and women | 69 (56.6%) | 367 (55.4%) | 420 (57.0%) | 187 (57.9%) |  |
| *% Total of agreement** | *17.2%* | *16.5%* | *13.4%* | *18.8%* |  |
| **For men, more than for women, it is very important to have success in their job** | | | | | |
| Completely agree | 9 (7.4%) | 9 (1.4%) | 5 (0.7%) | 6 (1.9%) | **0.006** |
| Strongly agree | 6 (4.9%) | 24 (3.6%) | 28 (3.8%) | 20 (6.2%) |  |
| Somewhat agree | 19 (15.6%) | 83 (12.5%) | 110 (14.9%) | 49 (15.2%) |  |
| Slightly disagree | 13 (10.7%) | 70 (10.6%) | 93 (12.6%) | 36 (11.1%) |  |
| Strongly disagree | 16 (13.1%) | 131 (19.8%) | 147 (19.9%) | 71 (22.0%) |  |
| No difference between men and women | 59 (48.4%) | 345 (52.1%) | 354 (48.0%) | 141 (43.7%) |  |
| *% Total of agreement** | *27.9%* | *17.5%* | *19.4%* | *23.3%* |  |
| **Women are more generous than men** | | | | | |
| Completely agree | 2 (1.6%) | 5 (0.8%) | 3 (0.4%) | 1 (0.3%) | **0.002** |
| Strongly agree | 6 (4.9%) | 6 (0.9%) | 8 (1.1%) | 5 (1.5%) |  |
| Somewhat agree | 13 (10.7%) | 49 (7.4%) | 54 (7.3%) | 28 (8.7%) |  |
| Slightly disagree | 16 (13.1%) | 74 (11.2%) | 71 (9.6%) | 28 (8.7%) |  |
| Strongly disagree | 20 (16.4%) | 110 (16.6%) | 107 (14.5%) | 44 (13.6%) |  |
| No difference between men and women | 65 (53.3%) | 418 (63.1%) | 494 (67.0%) | 217 (67.2%) |  |
| *% Total of agreement** | *17.2%* | *9.1%* | *8.8%* | *10.5%* |  |
| **Women are nicer than men** | | | | | |
| Completely agree | 3 (2.5%) | 8 (1.2%) | 3 (0.4%) | 0 (0.0%) | **< 0.001** |
| Strongly agree | 1 (0.8%) | 8 (1.2%) | 5 (0.7%) | 2 (0.6%) |  |
| Somewhat agree | 6 (4.9%) | 24 (3.6%) | 15 (2.0%) | 11 (3.4%) |  |
| Slightly disagree | 20 (16.4%) | 58 (8.8%) | 56 (7.6%) | 17 (5.3%) |  |
| Strongly disagree | 24 (19.7%) | 113 (17.1%) | 115 (15.6%) | 49 (15.2%) |  |
| No difference between men and women | 68 (55.7%) | 451 (68.1%) | 543 (73.7%) | 244 (75.5%) |  |
| *% Total of agreement** | *8.2%* | *6.0%* | *3.1%* | *4.0%* |  |
| **Women are more competitive than men** | | | | | |
| Completely agree | 6 (4.9%) | 6 (0.9%) | 13 (1.8%) | 5 (1.5%) | **0.002** |
| Strongly agree | 4 (3.3%) | 26 (3.9%) | 21 (2.8%) | 6 (1.9%) |  |
| Somewhat agree | 18 (14.8%) | 85 (12.8%) | 76 (10.3%) | 34 (10.5%) |  |
| Slightly disagree | 20 (16.4%) | 73 (11.0%) | 76 (10.3%) | 35 (10.8%) |  |
| Strongly disagree | 21 (17.2%) | 95 (14.4%) | 111 (15.1%) | 45 (13.9%) |  |
| No difference between men and women | 53 (43.4%) | 377 (56.9%) | 440 (59.7%) | 198 (61.3%) |  |
| *% Total of agreement** | *23.0%* | *17.6%* | *14.9%* | *13.9%* |  |
| **Women are more selfish than men** | | | | | |
| Completely agree | 6 (4.9%) | 3 (0.5%) | 1 (0.1%) | 0 (0.0%) | **< 0.001** |
| Strongly agree | 7 (5.7%) | 3 (0.5%) | 5 (0.7%) | 1 (0.3%) |  |
| Somewhat agree | 10 (8.2%) | 29 (4.4%) | 15 (2.0%) | 6 (1.9%) |  |
| Slightly disagree | 11 (9.0%) | 58 (8.8%) | 56 (7.6%) | 32 (9.9%) |  |
| Strongly disagree | 32 (26.2%) | 167 (25.2%) | 187 (25.4%) | 70 (21.7%) |  |
| No difference between men and women | 56 (45.9%) | 402 (60.7%) | 473 (64.2%) | 214 (66.3%) |  |
| *% Total of agreement** | *18.8%* | *5.4%* | *2.8%* | *2.2%* |  |
| **Women are more sensitive than men** | | | | | |
| Completely agree | 16 (13.1%) | 18 (2.7%) | 13 (1.8%) | 4 (1.2%) | **< 0.001** |
| Strongly agree | 23 (18.9%) | 42 (6.3%) | 32 (4.3%) | 18 (5.6%) |  |
| Somewhat agree | 22 (18.0%) | 160 (24.2%) | 183 (24.8%) | 86 (26.6%) |  |
| Slightly disagree | 8 (6.6%) | 83 (12.5%) | 83 (11.3%) | 42 (13.0%) |  |
| Strongly disagree | 9 (7.4%) | 73 (11.0%) | 70 (9.5%) | 30 (9.3%) |  |
| No difference between men and women | 44 (36.1%) | 286 (43.2%) | 356 (48.3%) | 143 (44.3%) |  |
| *% Total of agreement** | *50.0%* | *33.2%* | *30.9%* | *33.4%* |  |
| **Women are more determined than men** | | | | | |
| Completely agree | 6 (4.9%) | 12 (1.8%) | 10 (1.4%) | 3 (0.9%) | **< 0.001** |
| Strongly agree | 6 (4.9%) | 36 (5.4%) | 30 (4.1%) | 12 (3.7%) |  |
| Somewhat agree | 27 (22.1%) | 127 (19.2%) | 108 (14.7%) | 54 (16.7%) |  |
| Slightly disagree | 13 (10.7%) | 38 (5.7%) | 50 (6.8%) | 26 (8.0%) |  |
| Strongly disagree | 14 (11.5%) | 68 (10.3%) | 56 (7.6%) | 25 (7.7%) |  |
| No difference between men and women | 56 (45.9%) | 381 (57.6%) | 483 (65.5%) | 203 (62.8%) |  |
| *% Total of agreement** | *31.9%* | *26.4%* | *20.2%* | *21.3%* |  |
| **Women are more disorganized than men** | | | | | |
| Completely agree | 3 (2.5%) | 2 (0.3%) | 3 (0.4%) | 2 (0.6%) | **0.006** |
| Strongly agree | 1 (0.8%) | 0 (0.0%) | 1 (0.1%) | 1 (0.3%) |  |
| Somewhat agree | 10 (8.2%) | 19 (2.9%) | 15 (2.0%) | 2 (0.6%) |  |
| Slightly disagree | 10 (8.2%) | 47 (7.1%) | 45 (6.1%) | 22 (6.8%) |  |
| Strongly disagree | 40 (32.8%) | 282 (42.6%) | 306 (41.5%) | 140 (43.3%) |  |
| No difference between men and women | 58 (47.5%) | 312 (47.1%) | 367 (49.8%) | 156 (48.3%) |  |
| *% Total of agreement** | *11.5%* | *3.2%* | *2.5%* | *1.5%* |  |
| **Women are more stubborn than men** | | | | | |
| Completely agree | 9 (7.4%) | 10 (1.5%) | 12 (1.6%) | 5 (1.5%) | **< 0.001** |
| Strongly agree | 13 (10.7%) | 27 (4.1%) | 16 (2.2%) | 10 (3.1%) |  |
| Somewhat agree | 22 (18.0%) | 90 (13.6%) | 76 (10.3%) | 33 (10.2%) |  |
| Slightly disagree | 7 (5.7%) | 51 (7.7%) | 40 (5.4%) | 18 (5.6%) |  |
| Strongly disagree | 8 (6.6%) | 97 (14.7%) | 89 (12.1%) | 38 (11.8%) |  |
| No difference between men and women | 63 (51.6%) | 387 (58.5%) | 504 (68.4%) | 219 (67.8%) |  |
| *% Total of agreement** | *36.1%* | *19.2%* | *14.1%* | *14.8%* |  |
| **Men are more aggressive than women** | | | | | |
| Completely agree | 9 (7.4%) | 12 (1.8%) | 10 (1.4%) | 6 (1.9%) | **0.003** |
| Strongly agree | 7 (5.7%) | 26 (3.9%) | 27 (3.7%) | 16 (5.0%) |  |
| Somewhat agree | 24 (19.7%) | 117 (17.7%) | 119 (16.1%) | 63 (19.5%) |  |
| Slightly disagree | 14 (11.5%) | 77 (11.6%) | 97 (13.2%) | 46 (14.2%) |  |
| Strongly disagree | 16 (13.1%) | 81 (12.2%) | 82 (11.1%) | 32 (9.9%) |  |
| No difference between men and women | 52 (42.6%) | 349 (52.7%) | 402 (54.5%) | 160 (49.5%) |  |
| *% Total of agreement** | *32.8%* | *23.4%* | *21.2%* | *26.4%* |  |
| **Women are more cunning than men** | | | | | |
| Completely agree | 15 (12.3%) | 26 (3.9%) | 19 (2.6%) | 7 (2.2%) | **< 0.001** |
| Strongly agree | 9 (7.4%) | 30 (4.5%) | 26 (3.5%) | 10 (3.1%) |  |
| Somewhat agree | 19 (15.6%) | 104 (15.7%) | 95 (12.9%) | 34 (10.5%) |  |
| Slightly disagree | 9 (7.4%) | 51 (7.7%) | 58 (7.9%) | 28 (8.7%) |  |
| Strongly disagree | 14 (11.5%) | 72 (10.9%) | 66 (9.0%) | 26 (8.0%) |  |
| No difference between men and women | 56 (45.9%) | 379 (57.3%) | 473 (64.2%) | 218 (67.5%) |  |
| *% Total of agreement** | *35.3%* | *24.1%* | *19.0%* | *15.8%* |  |

*Note: The total percentage of agreement was calculated by summing the percentages of responses “completely agree”, “strongly agree”, and “somewhat agree”. This value is reported only for descriptive purposes and was not considered for the statistical analysis.

**Table 5Sa:** Stratification analyses conducted between each question composing the latent construct “Profession” (total of 10 questions) and age categories. Results containing p-values < 0.05 are highlighted in bold.

|  | **15-18 (N=74)** | **19-22 (N=223)** | **23-30 (N=347)** | **31-40 (N=336)** | **41-50 (N=304)** | **51-60 (N=296)** | **61-70 (N=214)** | **Over 70 (N=50)** | **p-value** |
| --- | --- | --- | --- | --- | --- | --- | --- | --- | --- |
| **The surgical profession is a job for men** | | | | | | | | | |
| Completely agree | 2 (2.7%) | 1 (0.4%) | 0 (0.0%) | 0 (0.0%) | 2 (0.7%) | 1 (0.3%) | 0 (0.0%) | 0 (0.0%) | **< 0.001** |
| Strongly agree | 3 (4.1%) | 1 (0.4%) | 2 (0.6%) | 4 (1.2%) | 3 (1.0%) | 2 (0.7%) | 1 (0.5%) | 0 (0.0%) |  |
| Somewhat agree | 4 (5.4%) | 1 (0.4%) | 8 (2.3%) | 11 (3.3%) | 16 (5.3%) | 10 (3.4%) | 8 (3.7%) | 2 (4.0%) |  |
| Slightly disagree | 11 (14.9%) | 13 (5.8%) | 22 (6.3%) | 34 (10.1%) | 29 (9.5%) | 31 (10.5%) | 26 (12.1%) | 5 (10.0%) |  |
| Strongly disagree | 21 (28.4%) | 83 (37.2%) | 98 (28.2%) | 100 (29.8%) | 111 (36.5%) | 115 (38.9%) | 84 (39.3%) | 30 (60.0%) |  |
| No difference between men and women | 33 (44.6%) | 124 (55.6%) | 217 (62.5%) | 187 (55.7%) | 143 (47.0%) | 137 (46.3%) | 95 (44.4%) | 13 (26.0%) |  |
| *% Total of agreement** | *12.2%* | *1.2%* | *2.9%* | *4.5%* | *7.0%* | *4.4%* | *4.2%* | *4.0%* |  |
| **Being a bus driver is a job for women** | | | | | | | | | |
| Completely agree | 0 (0.0%) | 0 (0.0%) | 1 (0.3%) | 2 (0.6%) | 2 (0.7%) | 4 (1.4%) | 3 (1.4%) | 1 (2.0%) | **0.013** |
| Strongly agree | 1 (1.4%) | 2 (0.9%) | 1 (0.3%) | 5 (1.5%) | 6 (2.0%) | 3 (1.0%) | 1 (0.5%) | 1 (2.0%) |  |
| Somewhat agree | 3 (4.1%) | 7 (3.1%) | 8 (2.3%) | 13 (3.9%) | 17 (5.6%) | 13 (4.4%) | 4 (1.9%) | 2 (4.0%) |  |
| Slightly disagree | 12 (16.2%) | 27 (12.1%) | 47 (13.5%) | 56 (16.7%) | 45 (14.8%) | 28 (9.5%) | 27 (12.6%) | 7 (14.0%) |  |
| Strongly disagree | 26 (35.1%) | 48 (21.5%) | 47 (13.5%) | 38 (11.3%) | 58 (19.1%) | 65 (22.0%) | 38 (17.8%) | 11 (22.0%) |  |
| No difference between men and women | 32 (43.2%) | 139 (62.3%) | 243 (70.0%) | 222 (66.1%) | 176 (57.9%) | 183 (61.8%) | 141 (65.9%) | 28 (56.0%) |  |
| *% Total of agreement** | *5.5%* | *4.0%* | *2.9%* | *6.0%* | *8.3%* | *6.8%* | *3.8%* | *8.0%* |  |
| **The military profession is for men** | | | | | | | | | |
| Completely agree | 14 (18.9%) | 3 (1.3%) | 2 (0.6%) | 8 (2.4%) | 8 (2.6%) | 9 (3.0%) | 6 (2.8%) | 1 (2.0%) | **< 0.001** |
| Strongly agree | 14 (18.9%) | 13 (5.8%) | 16 (4.6%) | 17 (5.1%) | 13 (4.3%) | 12 (4.1%) | 8 (3.7%) | 2 (4.0%) |  |
| Somewhat agree | 9 (12.2%) | 49 (22.0%) | 52 (15.0%) | 71 (21.1%) | 63 (20.7%) | 52 (17.6%) | 39 (18.2%) | 6 (12.0%) |  |
| Slightly disagree | 5 (6.8%) | 31 (13.9%) | 57 (16.4%) | 46 (13.7%) | 57 (18.8%) | 58 (19.6%) | 46 (21.5%) | 17 (34.0%) |  |
| Strongly disagree | 12 (16.2%) | 40 (17.9%) | 45 (13.0%) | 46 (13.7%) | 47 (15.5%) | 51 (17.2%) | 37 (17.3%) | 8 (16.0%) |  |
| No difference between men and women | 20 (27.0%) | 87 (39.0%) | 175 (50.4%) | 148 (44.0%) | 116 (38.2%) | 114 (38.5%) | 78 (36.4%) | 16 (32.0%) |  |
| *% Total of agreement** | *50.0%* | *29.1%* | *20.2%* | *28.6%* | *27.6%* | *24.7%* | *24.7%* | *18.0%* |  |
| **Nursing profession is for women** | | | | | | | | | |
| Completely agree | 4 (5.4%) | 0 (0.0%) | 2 (0.6%) | 0 (0.0%) | 1 (0.3%) | 0 (0.0%) | 0 (0.0%) | 0 (0.0%) | **< 0.001** |
| Strongly agree | 2 (2.7%) | 4 (1.8%) | 2 (0.6%) | 5 (1.5%) | 1 (0.3%) | 4 (1.4%) | 2 (0.9%) | 2 (4.0%) |  |
| Somewhat agree | 16 (21.6%) | 17 (7.6%) | 15 (4.3%) | 14 (4.2%) | 14 (4.6%) | 11 (3.7%) | 6 (2.8%) | 4 (8.0%) |  |
| Slightly disagree | 5 (6.8%) | 21 (9.4%) | 33 (9.5%) | 30 (8.9%) | 24 (7.9%) | 26 (8.8%) | 28 (13.1%) | 8 (16.0%) |  |
| Strongly disagree | 11 (14.9%) | 31 (13.9%) | 47 (13.5%) | 44 (13.1%) | 55 (18.1%) | 66 (22.3%) | 38 (17.8%) | 10 (20.0%) |  |
| No difference between men and women | 36 (48.6%) | 150 (67.3%) | 248 (71.5%) | 243 (72.3%) | 209 (68.8%) | 189 (63.9%) | 140 (65.4%) | 26 (52.0%) |  |
| *% Total of agreement** | *29.7%* | *9.4%* | *5.5%* | *5.7%* | *5.2%* | *5.1%* | *3.7%* | *12.0%* |  |
| **Working in a butcher shop is for men** | | | | | | | | | |
| Completely agree | 12 (16.2%) | 1 (0.4%) | 3 (0.9%) | 2 (0.6%) | 6 (2.0%) | 4 (1.4%) | 3 (1.4%) | 4 (8.0%) | **< 0.001** |
| Strongly agree | 6 (8.1%) | 2 (0.9%) | 3 (0.9%) | 6 (1.8%) | 5 (1.6%) | 6 (2.0%) | 6 (2.8%) | 3 (6.0%) |  |
| Somewhat agree | 13 (17.6%) | 25 (11.2%) | 21 (6.1%) | 41 (12.2%) | 47 (15.5%) | 40 (13.5%) | 43 (20.1%) | 12 (24.0%) |  |
| Slightly disagree | 3 (4.1%) | 24 (10.8%) | 46 (13.3%) | 30 (8.9%) | 35 (11.5%) | 43 (14.5%) | 29 (13.6%) | 6 (12.0%) |  |
| Strongly disagree | 11 (14.9%) | 34 (15.2%) | 45 (13.0%) | 45 (13.4%) | 50 (16.4%) | 60 (20.3%) | 35 (16.4%) | 7 (14.0%) |  |
| No difference between men and women | 29 (39.2%) | 137 (61.4%) | 229 (66.0%) | 212 (63.1%) | 161 (53.0%) | 143 (48.3%) | 98 (45.8%) | 18 (36.0%) |  |
| *% Total of agreement** | *41.9%* | *12.5%* | *7.9%* | *14.6%* | *19.1%* | *16.9%* | *24.3%* | *38.0%* |  |
| **Men are more inclined towards scientific subjects than women** | | | | | | | | | |
| Completely agree | 2 (2.7%) | 1 (0.4%) | 0 (0.0%) | 1 (0.3%) | 0 (0.0%) | 1 (0.3%) | 0 (0.0%) | 0 (0.0%) | **< 0.001** |
| Strongly agree | 2 (2.7%) | 2 (0.9%) | 0 (0.0%) | 1 (0.3%) | 3 (1.0%) | 0 (0.0%) | 0 (0.0%) | 1 (2.0%) |  |
| Somewhat agree | 3 (4.1%) | 8 (3.6%) | 9 (2.6%) | 15 (4.5%) | 11 (3.6%) | 16 (5.4%) | 7 (3.3%) | 2 (4.0%) |  |
| Slightly disagree | 13 (17.6%) | 8 (3.6%) | 12 (3.5%) | 18 (5.4%) | 20 (6.6%) | 26 (8.8%) | 23 (10.7%) | 5 (10.0%) |  |
| Strongly disagree | 17 (23.0%) | 53 (23.8%) | 85 (24.5%) | 75 (22.3%) | 71 (23.4%) | 93 (31.4%) | 74 (34.6%) | 22 (44.0%) |  |
| No difference between men and women | 37 (50.0%) | 151 (67.7%) | 241 (69.5%) | 226 (67.3%) | 199 (65.5%) | 160 (54.1%) | 110 (51.4%) | 20 (40.0%) |  |
| *% Total of agreement** | *9.5%* | *4.9%* | *2.6%* | *5.1%* | *4.6%* | *5.7%* | *3.3%* | *6.0%* |  |
| **The profession of psychotherapist is for women** | | | | | | | | | |
| Completely agree | 5 (6.8%) | 1 (0.4%) | 2 (0.6%) | 0 (0.0%) | 2 (0.7%) | 0 (0.0%) | 0 (0.0%) | 0 (0.0%) | **< 0.001** |
| Strongly agree | 6 (8.1%) | 2 (0.9%) | 0 (0.0%) | 2 (0.6%) | 2 (0.7%) | 0 (0.0%) | 0 (0.0%) | 1 (2.0%) |  |
| Somewhat agree | 6 (8.1%) | 6 (2.7%) | 8 (2.3%) | 7 (2.1%) | 7 (2.3%) | 7 (2.4%) | 7 (3.3%) | 1 (2.0%) |  |
| Slightly disagree | 7 (9.5%) | 11 (4.9%) | 12 (3.5%) | 10 (3.0%) | 15 (4.9%) | 15 (5.1%) | 15 (7.0%) | 5 (10.0%) |  |
| Strongly disagree | 8 (10.8%) | 39 (17.5%) | 45 (13.0%) | 44 (13.1%) | 51 (16.8%) | 60 (20.3%) | 43 (20.1%) | 12 (24.0%) |  |
| No difference between men and women | 42 (56.8%) | 164 (73.5%) | 280 (80.7%) | 273 (81.2%) | 227 (74.7%) | 214 (72.3%) | 149 (69.6%) | 31 (62.0%) |  |
| *% Total of agreement** | *23.0%* | *4.0%* | *2.9%* | *2.7%* | *3.7%* | *2.4%* | *3.3%* | *4.0%* |  |
| **Working as a babysitter is for women** | | | | | | | | | |
| Completely agree | 10 (13.5%) | 2 (0.9%) | 6 (1.7%) | 7 (2.1%) | 8 (2.6%) | 7 (2.4%) | 6 (2.8%) | 3 (6.0%) | **< 0.001** |
| Strongly agree | 10 (13.5%) | 8 (3.6%) | 11 (3.2%) | 19 (5.7%) | 15 (4.9%) | 11 (3.7%) | 9 (4.2%) | 4 (8.0%) |  |
| Somewhat agree | 7 (9.5%) | 31 (13.9%) | 56 (16.1%) | 77 (22.9%) | 74 (24.3%) | 58 (19.6%) | 62 (29.0%) | 15 (30.0%) |  |
| Slightly disagree | 11 (14.9%) | 25 (11.2%) | 42 (12.1%) | 41 (12.2%) | 41 (13.5%) | 58 (19.6%) | 32 (15.0%) | 10 (20.0%) |  |
| Strongly disagree | 10 (13.5%) | 33 (14.8%) | 42 (12.1%) | 32 (9.5%) | 38 (12.5%) | 46 (15.5%) | 33 (15.4%) | 4 (8.0%) |  |
| No difference between men and women | 26 (35.1%) | 124 (55.6%) | 190 (54.8%) | 160 (47.6%) | 128 (42.1%) | 116 (39.2%) | 72 (33.6%) | 14 (28.0%) |  |
| *% Total of agreement** | *36.5%* | *18.4%* | *21.0%* | *30.7%* | *31.8%* | *25.7%* | *36.0%* | *44.0%* |  |
| **Being a pilot is a job for men** | | | | | | | | | |
| Completely agree | 13 (17.6%) | 4 (1.8%) | 1 (0.3%) | 3 (0.9%) | 1 (0.3%) | 3 (1.0%) | 0 (0.0%) | 0 (0.0%) | **< 0.001** |
| Strongly agree | 4 (5.4%) | 4 (1.8%) | 5 (1.4%) | 7 (2.1%) | 5 (1.6%) | 3 (1.0%) | 1 (0.5%) | 1 (2.0%) |  |
| Somewhat agree | 7 (9.5%) | 15 (6.7%) | 12 (3.5%) | 18 (5.4%) | 20 (6.6%) | 12 (4.1%) | 8 (3.7%) | 6 (12.0%) |  |
| Slightly disagree | 5 (6.8%) | 19 (8.5%) | 25 (7.2%) | 30 (8.9%) | 20 (6.6%) | 27 (9.1%) | 29 (13.6%) | 9 (18.0%) |  |
| Strongly disagree | 10 (13.5%) | 47 (21.1%) | 72 (20.7%) | 52 (15.5%) | 76 (25.0%) | 81 (27.4%) | 70 (32.7%) | 15 (30.0%) |  |
| No difference between men and women | 35 (47.3%) | 134 (60.1%) | 232 (66.9%) | 226 (67.3%) | 182 (59.9%) | 170 (57.4%) | 106 (49.5%) | 19 (38.0%) |  |
| *% Total of agreement** | *32.5%* | *10.3%* | *5.2%* | *8.4%* | *8.5%* | *6.1%* | *4.2%* | *14.0%* |  |
| **The surgical profession is a job for women** | | | | | | | | | |
| Completely agree | 1 (1.4%) | 0 (0.0%) | 1 (0.3%) | 2 (0.6%) | 1 (0.3%) | 1 (0.3%) | 1 (0.5%) | 0 (0.0%) | **< 0.001** |
| Strongly agree | 0 (0.0%) | 1 (0.4%) | 3 (0.9%) | 3 (0.9%) | 1 (0.3%) | 3 (1.0%) | 2 (0.9%) | 0 (0.0%) |  |
| Somewhat agree | 9 (12.2%) | 4 (1.8%) | 8 (2.3%) | 4 (1.2%) | 8 (2.6%) | 4 (1.4%) | 5 (2.3%) | 3 (6.0%) |  |
| Slightly disagree | 9 (12.2%) | 3 (1.3%) | 16 (4.6%) | 22 (6.5%) | 22 (7.2%) | 19 (6.4%) | 18 (8.4%) | 3 (6.0%) |  |
| Strongly disagree | 9 (12.2%) | 33 (14.8%) | 28 (8.1%) | 31 (9.2%) | 38 (12.5%) | 41 (13.9%) | 29 (13.6%) | 9 (18.0%) |  |
| No difference between men and women | 46 (62.2%) | 182 (81.6%) | 291 (83.9%) | 274 (81.5%) | 234 (77.0%) | 228 (77.0%) | 159 (74.3%) | 35 (70.0%) |  |
| *% Total of agreement** | *13.6%* | *2.2%* | *3.5%* | *2.7%* | *1.2%* | *2.7%* | *3.7%* | *6.0%* |  |

*Note: The total percentage of agreement was calculated by summing the percentages of responses “completely agree”, “strongly agree”, and “somewhat agree”. This value is reported only for descriptive purposes and was not considered for the statistical analysis.

**Table 5Sb:** Stratification analyses conducted between each question composing the latent construct “Profession” (total of 10 questions) and gender categories. Results containing p-values < 0.05 are highlighted in bold.

|  | **Female (N=1292)** | **Male (N=534)** | **Other/Do not want to answer (N=18)** | **p-value** |
| --- | --- | --- | --- | --- |
| **The surgical profession is a job for men** | | | | |
| Completely agree | 1 (0.1%) | 5 (0.9%) | 0 (0.0%) | **< 0.001** |
| Strongly agree | 8 (0.6%) | 8 (1.5%) | 0 (0.0%) |  |
| Somewhat agree | 34 (2.6%) | 26 (4.9%) | 0 (0.0%) |  |
| Slightly disagree | 95 (7.4%) | 76 (14.2%) | 0 (0.0%) |  |
| Strongly disagree | 498 (38.5%) | 139 (26.0%) | 5 (27.8%) |  |
| No difference between men and women | 656 (50.8%) | 280 (52.4%) | 13 (72.2%) |  |
| *% Total of agreement** | *3.3%* | *7.3%* | *0.0%* |  |
| **Being a bus driver is a job for women** | | | | |
| Completely agree | 12 (0.9%) | 1 (0.2%) | 0 (0.0%) | **0.013** |
| Strongly agree | 15 (1.2%) | 5 (0.9%) | 0 (0.0%) |  |
| Somewhat agree | 45 (3.5%) | 21 (3.9%) | 1 (5.6%) |  |
| Slightly disagree | 151 (11.7%) | 98 (18.4%) | 0 (0.0%) |  |
| Strongly disagree | 222 (17.2%) | 107 (20.0%) | 2 (11.1%) |  |
| No difference between men and women | 847 (65.6%) | 302 (56.6%) | 15 (83.3%) |  |
| *% Total of agreement** | *5.6%* | *5.0%* | *5.6%* |  |
| **The military profession is for men** | | | | |
| Completely agree | 19 (1.5%) | 30 (5.6%) | 2 (11.1%) | **< 0.001** |
| Strongly agree | 52 (4.0%) | 43 (8.1%) | 0 (0.0%) |  |
| Somewhat agree | 220 (17.0%) | 118 (22.1%) | 3 (16.7%) |  |
| Slightly disagree | 239 (18.5%) | 78 (14.6%) | 0 (0.0%) |  |
| Strongly disagree | 237 (18.3%) | 46 (8.6%) | 3 (16.7%) |  |
| No difference between men and women | 525 (40.6%) | 219 (41.0%) | 10 (55.6%) |  |
| *% Total of agreement** | *22.5%* | *35.8%* | *27.8%* |  |
| **Nursing profession is for women** | | | | |
| Completely agree | 0 (0.0%) | 7 (1.3%) | 0 (0.0%) | **< 0.001** |
| Strongly agree | 8 (0.6%) | 14 (2.6%) | 0 (0.0%) |  |
| Somewhat agree | 49 (3.8%) | 47 (8.8%) | 1 (5.6%) |  |
| Slightly disagree | 102 (7.9%) | 71 (13.3%) | 2 (11.1%) |  |
| Strongly disagree | 247 (19.1%) | 54 (10.1%) | 1 (5.6%) |  |
| No difference between men and women | 886 (68.6%) | 341 (63.9%) | 14 (77.8%) |  |
| *% Total of agreement** | *4.4%* | *12.7%* | *5.6%* |  |
| **Working in a butcher shop is for men** | | | | |
| Completely agree | 14 (1.1%) | 20 (3.7%) | 1 (5.6%) | **< 0.001** |
| Strongly agree | 13 (1.0%) | 24 (4.5%) | 0 (0.0%) |  |
| Somewhat agree | 145 (11.2%) | 96 (18.0%) | 1 (5.6%) |  |
| Slightly disagree | 168 (13.0%) | 48 (9.0%) | 0 (0.0%) |  |
| Strongly disagree | 234 (18.1%) | 49 (9.2%) | 4 (22.2%) |  |
| No difference between men and women | 718 (55.6%) | 297 (55.6%) | 12 (66.7%) |  |
| *% Total of agreement** | *13.3%* | *26.2%* | *11.2%* |  |
| **Men are more inclined towards scientific subjects than women** | | | | |
| Completely agree | 2 (0.2%) | 3 (0.6%) | 0 (0.0%) | 0.235 |
| Strongly agree | 2 (0.2%) | 6 (1.1%) | 1 (5.6%) |  |
| Somewhat agree | 47 (3.6%) | 24 (4.5%) | 0 (0.0%) |  |
| Slightly disagree | 71 (5.5%) | 54 (10.1%) | 0 (0.0%) |  |
| Strongly disagree | 384 (29.7%) | 102 (19.1%) | 4 (22.2%) |  |
| No difference between men and women | 786 (60.8%) | 345 (64.6%) | 13 (72.2%) |  |
| *% Total of agreement** | *4.0%* | *6.2%* | *5.6%* |  |
| **The profession of psychotherapist is for women** | | | | |
| Completely agree | 0 (0.0%) | 10 (1.9%) | 0 (0.0%) | **< 0.001** |
| Strongly agree | 1 (0.1%) | 11 (2.1%) | 1 (5.6%) |  |
| Somewhat agree | 27 (2.1%) | 22 (4.1%) | 0 (0.0%) |  |
| Slightly disagree | 49 (3.8%) | 41 (7.7%) | 0 (0.0%) |  |
| Strongly disagree | 229 (17.7%) | 70 (13.1%) | 3 (16.7%) |  |
| No difference between men and women | 986 (76.3%) | 380 (71.2%) | 14 (77.8%) |  |
| *% Total of agreement** | *2.2%* | *8.1%* | *5.6%* |  |
| **Working as a babysitter is for women** | | | | |
| Completely agree | 18 (1.4%) | 31 (5.8%) | 0 (0.0%) | **< 0.001** |
| Strongly agree | 47 (3.6%) | 39 (7.3%) | 1 (5.6%) |  |
| Somewhat agree | 249 (19.3%) | 128 (24.0%) | 3 (16.7%) |  |
| Slightly disagree | 202 (15.6%) | 57 (10.7%) | 1 (5.6%) |  |
| Strongly disagree | 192 (14.9%) | 42 (7.9%) | 4 (22.2%) |  |
| No difference between men and women | 584 (45.2%) | 237 (44.4%) | 9 (50.0%) |  |
| *% Total of agreement** | *24.3%* | *37.1%* | *22.3%* |  |
| **Being a pilot is a job for men** | | | | |
| Completely agree | 6 (0.5%) | 19 (3.6%) | 0 (0.0%) | **< 0.001** |
| Strongly agree | 13 (1.0%) | 16 (3.0%) | 1 (5.6%) |  |
| Somewhat agree | 48 (3.7%) | 50 (9.4%) | 0 (0.0%) |  |
| Slightly disagree | 116 (9.0%) | 48 (9.0%) | 0 (0.0%) |  |
| Strongly disagree | 329 (25.5%) | 89 (16.7%) | 5 (27.8%) |  |
| No difference between men and women | 780 (60.4%) | 312 (58.4%) | 12 (66.7%) |  |
| *% Total of agreement** | *5.2%* | *16.0%* | *5.6%* |  |
| **The surgical profession is a job for women** | | | | |
| Completely agree | 6 (0.5%) | 1 (0.2%) | 0 (0.0%) | **0.019** |
| Strongly agree | 9 (0.7%) | 4 (0.7%) | 0 (0.0%) |  |
| Somewhat agree | 28 (2.2%) | 17 (3.2%) | 0 (0.0%) |  |
| Slightly disagree | 64 (5.0%) | 48 (9.0%) | 0 (0.0%) |  |
| Strongly disagree | 151 (11.7%) | 65 (12.2%) | 2 (11.1%) |  |
| No difference between men and women | 1034 (80.0%) | 399 (74.7%) | 16 (88.9%) |  |
| *% Total of agreement** | *3.4%* | *4.1%* | *0.0%* |  |

*Note: The total percentage of agreement was calculated by summing the percentages of responses “completely agree”, “strongly agree”, and “somewhat agree”. This value is reported only for descriptive purposes and was not considered for the statistical analysis.

**Table 5Sc:** Stratification analyses conducted between each question composing the latent construct “Profession” (total of 10 questions) and degree categories. Results containing p-values < 0.05 are highlighted in bold.

|  | **Elementary/Secondary school degrees (N=122)** | **High school degree (N=662)** | **University degree (N=737)** | **Post-University degree (N=323)** | **p-value** |
| --- | --- | --- | --- | --- | --- |
| **The surgical profession is a job for men** | | | | | |
| Completely agree | 3 (2.5%) | 3 (0.5%) | 0 (0.0%) | 0 (0.0%) | **0.001** |
| Strongly agree | 4 (3.3%) | 4 (0.6%) | 5 (0.7%) | 3 (0.9%) |  |
| Somewhat agree | 4 (3.3%) | 15 (2.3%) | 24 (3.3%) | 17 (5.3%) |  |
| Slightly disagree | 14 (11.5%) | 52 (7.9%) | 69 (9.4%) | 36 (11.1%) |  |
| Strongly disagree | 43 (35.2%) | 245 (37.0%) | 242 (32.8%) | 112 (34.7%) |  |
| No difference between men and women | 54 (44.3%) | 343 (51.8%) | 397 (53.9%) | 155 (48.0%) |  |
| *% Total of agreement** | *9.1%* | *3.4%* | *4.0%* | *6.2%* |  |
| **Being a bus driver is a job for women** | | | | | |
| Completely agree | 0 (0.0%) | 4 (0.6%) | 5 (0.7%) | 4 (1.2%) | 0.267 |
| Strongly agree | 3 (2.5%) | 5 (0.8%) | 9 (1.2%) | 3 (0.9%) |  |
| Somewhat agree | 5 (4.1%) | 24 (3.6%) | 26 (3.5%) | 12 (3.7%) |  |
| Slightly disagree | 16 (13.1%) | 91 (13.7%) | 91 (12.3%) | 51 (15.8%) |  |
| Strongly disagree | 31 (25.4%) | 130 (19.6%) | 115 (15.6%) | 55 (17.0%) |  |
| No difference between men and women | 67 (54.9%) | 408 (61.6%) | 491 (66.6%) | 198 (61.3%) |  |
| *% Total of agreement** | *6.6%* | *5.0%* | *5.4%* | *5.8%* |  |
| **The military profession is for men** | | | | | |
| Completely agree | 17 (13.9%) | 19 (2.9%) | 10 (1.4%) | 5 (1.5%) | **0.022** |
| Strongly agree | 10 (8.2%) | 37 (5.6%) | 30 (4.1%) | 18 (5.6%) |  |
| Somewhat agree | 14 (11.5%) | 126 (19.0%) | 145 (19.7%) | 56 (17.3%) |  |
| Slightly disagree | 17 (13.9%) | 94 (14.2%) | 140 (19.0%) | 66 (20.4%) |  |
| Strongly disagree | 16 (13.1%) | 118 (17.8%) | 110 (14.9%) | 42 (13.0%) |  |
| No difference between men and women | 48 (39.3%) | 268 (40.5%) | 302 (41.0%) | 136 (42.1%) |  |
| *% Total of agreement** | *33.6%* | *27.5%* | *25.2%* | *24.4%* |  |
| **Nursing profession is for women** | | | | | |
| Completely agree | 4 (3.3%) | 1 (0.2%) | 1 (0.1%) | 1 (0.3%) | **< 0.001** |
| Strongly agree | 3 (2.5%) | 9 (1.4%) | 5 (0.7%) | 5 (1.5%) |  |
| Somewhat agree | 17 (13.9%) | 35 (5.3%) | 30 (4.1%) | 15 (4.6%) |  |
| Slightly disagree | 11 (9.0%) | 62 (9.4%) | 77 (10.4%) | 25 (7.7%) |  |
| Strongly disagree | 19 (15.6%) | 117 (17.7%) | 120 (16.3%) | 46 (14.2%) |  |
| No difference between men and women | 68 (55.7%) | 438 (66.2%) | 504 (68.4%) | 231 (71.5%) |  |
| *% Total of agreement** | *19.7%* | *6.9%* | *4.9%* | *6.4%* |  |
| **Working in a butcher shop is for men** | | | | | |
| Completely agree | 16 (13.1%) | 8 (1.2%) | 6 (0.8%) | 5 (1.5%) | **< 0.001** |
| Strongly agree | 7 (5.7%) | 18 (2.7%) | 9 (1.2%) | 3 (0.9%) |  |
| Somewhat agree | 23 (18.9%) | 87 (13.1%) | 93 (12.6%) | 39 (12.1%) |  |
| Slightly disagree | 8 (6.6%) | 62 (9.4%) | 103 (14.0%) | 43 (13.3%) |  |
| Strongly disagree | 17 (13.9%) | 113 (17.1%) | 105 (14.2%) | 52 (16.1%) |  |
| No difference between men and women | 51 (41.8%) | 374 (56.5%) | 421 (57.1%) | 181 (56.0%) |  |
| *% Total of agreement** | *37.7%* | *17.0%* | *14.6%* | *14.5%* |  |
| **Men are more inclined towards scientific subjects than women** | | | | | |
| Completely agree | 2 (1.6%) | 3 (0.5%) | 0 (0.0%) | 0 (0.0%) | **0.023** |
| Strongly agree | 3 (2.5%) | 3 (0.5%) | 2 (0.3%) | 1 (0.3%) |  |
| Somewhat agree | 4 (3.3%) | 27 (4.1%) | 31 (4.2%) | 9 (2.8%) |  |
| Slightly disagree | 17 (13.9%) | 47 (7.1%) | 41 (5.6%) | 20 (6.2%) |  |
| Strongly disagree | 24 (19.7%) | 176 (26.6%) | 200 (27.1%) | 90 (27.9%) |  |
| No difference between men and women | 72 (59.0%) | 406 (61.3%) | 463 (62.8%) | 203 (62.8%) |  |
| *% Total of agreement** | *7.4%* | *5.1%* | *4.5%* | *3.1%* |  |
| **The profession of psychotherapist is for women** | | | | | |
| Completely agree | 6 (4.9%) | 2 (0.3%) | 2 (0.3%) | 0 (0.0%) | **< 0.001** |
| Strongly agree | 7 (5.7%) | 4 (0.6%) | 1 (0.1%) | 1 (0.3%) |  |
| Somewhat agree | 7 (5.7%) | 20 (3.0%) | 16 (2.2%) | 6 (1.9%) |  |
| Slightly disagree | 11 (9.0%) | 37 (5.6%) | 27 (3.7%) | 15 (4.6%) |  |
| Strongly disagree | 15 (12.3%) | 116 (17.5%) | 117 (15.9%) | 54 (16.7%) |  |
| No difference between men and women | 76 (62.3%) | 483 (73.0%) | 574 (77.9%) | 247 (76.5%) |  |
| *% Total of agreement** | *16.0%* | *3.9%* | *2.6%* | *2.2%* |  |
| **Working as a babysitter is for women** | | | | | |
| Completely agree | 13 (10.7%) | 15 (2.3%) | 10 (1.4%) | 11 (3.4%) | **0.018** |
| Strongly agree | 14 (11.5%) | 31 (4.7%) | 36 (4.9%) | 6 (1.9%) |  |
| Somewhat agree | 17 (13.9%) | 133 (20.1%) | 158 (21.4%) | 72 (22.3%) |  |
| Slightly disagree | 13 (10.7%) | 96 (14.5%) | 104 (14.1%) | 47 (14.6%) |  |
| Strongly disagree | 16 (13.1%) | 86 (13.0%) | 89 (12.1%) | 47 (14.6%) |  |
| No difference between men and women | 49 (40.2%) | 301 (45.5%) | 340 (46.1%) | 140 (43.3%) |  |
| *% Total of agreement** | *36.1%* | *27.1%* | *27.7%* | *27.6%* |  |
| **Being a pilot is a job for men** | | | | | |
| Completely agree | 12 (9.8%) | 8 (1.2%) | 3 (0.4%) | 2 (0.6%) | **< 0.001** |
| Strongly agree | 6 (4.9%) | 12 (1.8%) | 9 (1.2%) | 3 (0.9%) |  |
| Somewhat agree | 4 (3.3%) | 43 (6.5%) | 32 (4.3%) | 19 (5.9%) |  |
| Slightly disagree | 13 (10.7%) | 54 (8.2%) | 65 (8.8%) | 32 (9.9%) |  |
| Strongly disagree | 20 (16.4%) | 153 (23.1%) | 178 (24.2%) | 72 (22.3%) |  |
| No difference between men and women | 67 (54.9%) | 392 (59.2%) | 450 (61.1%) | 195 (60.4%) |  |
| *% Total of agreement** | *18.0%* | *9.5%* | *5.9%* | *7.4%* |  |
| **The surgical profession is a job for women** | | | | | |
| Completely agree | 1 (0.8%) | 0 (0.0%) | 5 (0.7%) | 1 (0.3%) | **< 0.001** |
| Strongly agree | 0 (0.0%) | 6 (0.9%) | 6 (0.8%) | 1 (0.3%) |  |
| Somewhat agree | 14 (11.5%) | 10 (1.5%) | 12 (1.6%) | 9 (2.8%) |  |
| Slightly disagree | 11 (9.0%) | 29 (4.4%) | 45 (6.1%) | 27 (8.4%) |  |
| Strongly disagree | 14 (11.5%) | 98 (14.8%) | 73 (9.9%) | 33 (10.2%) |  |
| No difference between men and women | 82 (67.2%) | 519 (78.4%) | 596 (80.9%) | 252 (78.0%) |  |
| *% Total of agreement** | *12.3%* | *2.4%* | *3.1%* | *3.4%* |  |

*Note: The total percentage of agreement was calculated by summing the percentages of responses “completely agree”, “strongly agree”, and “somewhat agree”. This value is reported only for descriptive purposes and was not considered for the statistical analysis.

**Table 6Sa:** Stratification analyses conducted between each question composing the latent construct “Sports” (total of 10 questions) and age categories. Results containing p-values < 0.05 are highlighted in bold.

|  | **15-18 (N=74)** | **19-22 (N=223)** | **23-30 (N=347)** | **31-40 (N=336)** | **41-50 (N=304)** | **51-60 (N=296)** | **61-70 (N=214)** | **Over 70 (N=50)** | **p-value** |
| --- | --- | --- | --- | --- | --- | --- | --- | --- | --- |
| **Tennis is a sport for males** | | | | | | | | | |
| Completely agree | 1 (1.4%) | 0 (0.0%) | 0 (0.0%) | 0 (0.0%) | 0 (0.0%) | 0 (0.0%) | 0 (0.0%) | 0 (0.0%) | **< 0.001** |
| Strongly agree | 0 (0.0%) | 1 (0.4%) | 1 (0.3%) | 0 (0.0%) | 0 (0.0%) | 1 (0.3%) | 0 (0.0%) | 0 (0.0%) |  |
| Somewhat agree | 10 (13.5%) | 2 (0.9%) | 2 (0.6%) | 5 (1.5%) | 2 (0.7%) | 1 (0.3%) | 0 (0.0%) | 0 (0.0%) |  |
| Slightly disagree | 8 (10.8%) | 10 (4.5%) | 12 (3.5%) | 14 (4.2%) | 16 (5.3%) | 16 (5.4%) | 12 (5.6%) | 3 (6.0%) |  |
| Strongly disagree | 13 (17.6%) | 62 (27.8%) | 63 (18.2%) | 62 (18.5%) | 87 (28.6%) | 87 (29.4%) | 74 (34.6%) | 18 (36.0%) |  |
| No difference between men and women | 42 (56.8%) | 148 (66.4%) | 269 (77.5%) | 255 (75.9%) | 199 (65.5%) | 191 (64.5%) | 128 (59.8%) | 29 (58.0%) |  |
| *% Total of agreement** | *14.9%* | *1.3%* | *0.9%* | *1.5%* | *0.7%* | *0.6%* | *0.0%* | *0.0%* |  |
| **Artistic gymnastics is for females** | | | | | | | | | |
| Completely agree | 8 (10.8%) | 3 (1.3%) | 5 (1.4%) | 7 (2.1%) | 9 (3.0%) | 3 (1.0%) | 2 (0.9%) | 1 (2.0%) | **< 0.001** |
| Strongly agree | 13 (17.6%) | 6 (2.7%) | 4 (1.2%) | 10 (3.0%) | 10 (3.3%) | 4 (1.4%) | 1 (0.5%) | 1 (2.0%) |  |
| Somewhat agree | 13 (17.6%) | 32 (14.3%) | 44 (12.7%) | 49 (14.6%) | 47 (15.5%) | 34 (11.5%) | 29 (13.6%) | 13 (26.0%) |  |
| Slightly disagree | 6 (8.1%) | 21 (9.4%) | 43 (12.4%) | 36 (10.7%) | 41 (13.5%) | 39 (13.2%) | 30 (14.0%) | 3 (6.0%) |  |
| Strongly disagree | 8 (10.8%) | 33 (14.8%) | 44 (12.7%) | 47 (14.0%) | 43 (14.1%) | 55 (18.6%) | 39 (18.2%) | 12 (24.0%) |  |
| No difference between men and women | 26 (35.1%) | 128 (57.4%) | 207 (59.7%) | 187 (55.7%) | 154 (50.7%) | 161 (54.4%) | 113 (52.8%) | 20 (40.0%) |  |
| *% Total of agreement** | *46.0%* | *18.3%* | *15.3%* | *19.7%* | *21.8%* | *13.9%* | *15.0%* | *30.0%* |  |
| **Skiing is a sport for males** | | | | | | | | | |
| Completely agree | 1 (1.4%) | 1 (0.4%) | 0 (0.0%) | 0 (0.0%) | 0 (0.0%) | 0 (0.0%) | 0 (0.0%) | 0 (0.0%) | **< 0.001** |
| Strongly agree | 0 (0.0%) | 0 (0.0%) | 0 (0.0%) | 0 (0.0%) | 0 (0.0%) | 0 (0.0%) | 0 (0.0%) | 0 (0.0%) |  |
| Somewhat agree | 10 (13.5%) | 0 (0.0%) | 2 (0.6%) | 1 (0.3%) | 1 (0.3%) | 1 (0.3%) | 0 (0.0%) | 1 (2.0%) |  |
| Slightly disagree | 4 (5.4%) | 4 (1.8%) | 6 (1.7%) | 8 (2.4%) | 7 (2.3%) | 14 (4.7%) | 9 (4.2%) | 1 (2.0%) |  |
| Strongly disagree | 14 (18.9%) | 55 (24.7%) | 68 (19.6%) | 63 (18.8%) | 77 (25.3%) | 84 (28.4%) | 68 (31.8%) | 20 (40.0%) |  |
| No difference between men and women | 45 (60.8%) | 163 (73.1%) | 271 (78.1%) | 264 (78.6%) | 219 (72.0%) | 197 (66.6%) | 137 (64.0%) | 28 (56.0%) |  |
| *% Total of agreement** | *14.9%* | *0.4%* | *0.6%* | *0.3%* | *0.3%* | *0.3%* | *0.0%* | *2.0%* |  |
| **Rugby is a sport for males** | | | | | | | | | |
| Completely agree | 22 (29.7%) | 4 (1.8%) | 8 (2.3%) | 12 (3.6%) | 14 (4.6%) | 14 (4.7%) | 8 (3.7%) | 6 (12.0%) | **< 0.001** |
| Strongly agree | 8 (10.8%) | 18 (8.1%) | 20 (5.8%) | 14 (4.2%) | 14 (4.6%) | 18 (6.1%) | 21 (9.8%) | 5 (10.0%) |  |
| Somewhat agree | 8 (10.8%) | 39 (17.5%) | 52 (15.0%) | 74 (22.0%) | 94 (30.9%) | 89 (30.1%) | 77 (36.0%) | 21 (42.0%) |  |
| Slightly disagree | 4 (5.4%) | 31 (13.9%) | 47 (13.5%) | 44 (13.1%) | 52 (17.1%) | 52 (17.6%) | 40 (18.7%) | 7 (14.0%) |  |
| Strongly disagree | 11 (14.9%) | 29 (13.0%) | 52 (15.0%) | 43 (12.8%) | 29 (9.5%) | 45 (15.2%) | 23 (10.7%) | 3 (6.0%) |  |
| No difference between men and women | 21 (28.4%) | 102 (45.7%) | 168 (48.4%) | 149 (44.3%) | 101 (33.2%) | 78 (26.4%) | 45 (21.0%) | 8 (16.0%) |  |
| *% Total of agreement** | *51.3%* | *27.4%* | *23.1%* | *29.8%* | *40.1%* | *40.9%* | *49.5%* | *64.0%* |  |
| **Cycling is a sport for males** | | | | | | | | | |
| Completely agree | 3 (4.1%) | 1 (0.4%) | 2 (0.6%) | 2 (0.6%) | 1 (0.3%) | 2 (0.7%) | 0 (0.0%) | 0 (0.0%) | **< 0.001** |
| Strongly agree | 3 (4.1%) | 3 (1.3%) | 1 (0.3%) | 2 (0.6%) | 3 (1.0%) | 2 (0.7%) | 1 (0.5%) | 1 (2.0%) |  |
| Somewhat agree | 11 (14.9%) | 14 (6.3%) | 17 (4.9%) | 15 (4.5%) | 28 (9.2%) | 20 (6.8%) | 17 (7.9%) | 9 (18.0%) |  |
| Slightly disagree | 8 (10.8%) | 16 (7.2%) | 25 (7.2%) | 24 (7.1%) | 27 (8.9%) | 34 (11.5%) | 33 (15.4%) | 8 (16.0%) |  |
| Strongly disagree | 9 (12.2%) | 38 (17.0%) | 59 (17.0%) | 59 (17.6%) | 62 (20.4%) | 65 (22.0%) | 47 (22.0%) | 10 (20.0%) |  |
| No difference between men and women | 40 (54.1%) | 151 (67.7%) | 243 (70.0%) | 234 (69.6%) | 183 (60.2%) | 173 (58.4%) | 116 (54.2%) | 22 (44.0%) |  |
| *% Total of agreement** | *23.1%* | *8.0%* | *5.8%* | *5.7%* | *10.5%* | *8.2%* | *8.4%* | *20.0%* |  |
| **Boxing is a sport for males** | | | | | | | | | |
| Completely agree | 14 (18.9%) | 2 (0.9%) | 7 (2.0%) | 8 (2.4%) | 15 (4.9%) | 18 (6.1%) | 10 (4.7%) | 3 (6.0%) | **< 0.001** |
| Strongly agree | 6 (8.1%) | 12 (5.4%) | 6 (1.7%) | 13 (3.9%) | 16 (5.3%) | 12 (4.1%) | 25 (11.7%) | 5 (10.0%) |  |
| Somewhat agree | 11 (14.9%) | 26 (11.7%) | 37 (10.7%) | 54 (16.1%) | 70 (23.0%) | 80 (27.0%) | 63 (29.4%) | 21 (42.0%) |  |
| Slightly disagree | 6 (8.1%) | 22 (9.9%) | 30 (8.6%) | 34 (10.1%) | 46 (15.1%) | 44 (14.9%) | 25 (11.7%) | 8 (16.0%) |  |
| Strongly disagree | 10 (13.5%) | 39 (17.5%) | 51 (14.7%) | 40 (11.9%) | 32 (10.5%) | 38 (12.8%) | 22 (10.3%) | 4 (8.0%) |  |
| No difference between men and women | 27 (36.5%) | 122 (54.7%) | 216 (62.2%) | 187 (55.7%) | 125 (41.1%) | 104 (35.1%) | 69 (32.2%) | 9 (18.0%) |  |
| *% Total of agreement** | *41.9%* | *18.0%* | *14.4%* | *22.4%* | *33.2%* | *37.2%* | *45.8%* | *58.0%* |  |
| **Karate is a sport for males** | | | | | | | | | |
| Completely agree | 7 (9.5%) | 1 (0.4%) | 2 (0.6%) | 2 (0.6%) | 3 (1.0%) | 1 (0.3%) | 0 (0.0%) | 3 (6.0%) | **< 0.001** |
| Strongly agree | 3 (4.1%) | 1 (0.4%) | 1 (0.3%) | 3 (0.9%) | 2 (0.7%) | 5 (1.7%) | 1 (0.5%) | 1 (2.0%) |  |
| Somewhat agree | 8 (10.8%) | 3 (1.3%) | 9 (2.6%) | 6 (1.8%) | 7 (2.3%) | 5 (1.7%) | 14 (6.5%) | 3 (6.0%) |  |
| Slightly disagree | 4 (5.4%) | 16 (7.2%) | 12 (3.5%) | 17 (5.1%) | 24 (7.9%) | 13 (4.4%) | 21 (9.8%) | 7 (14.0%) |  |
| Strongly disagree | 12 (16.2%) | 50 (22.4%) | 62 (17.9%) | 49 (14.6%) | 70 (23.0%) | 79 (26.7%) | 62 (29.0%) | 13 (26.0%) |  |
| No difference between men and women | 40 (54.1%) | 152 (68.2%) | 261 (75.2%) | 259 (77.1%) | 198 (65.1%) | 193 (65.2%) | 116 (54.2%) | 23 (46.0%) |  |
| *% Total of agreement** | *24.4%* | *2.1%* | *3.5%* | *3.3%* | *4.0%* | *3.7%* | *7.0%* | *14.0%* |  |
| **Football is a sport for males** | | | | | | | | | |
| Completely agree | 14 (18.9%) | 8 (3.6%) | 3 (0.9%) | 6 (1.8%) | 6 (2.0%) | 4 (1.4%) | 1 (0.5%) | 1 (2.0%) | **< 0.001** |
| Strongly agree | 8 (10.8%) | 5 (2.2%) | 6 (1.7%) | 9 (2.7%) | 9 (3.0%) | 7 (2.4%) | 4 (1.9%) | 2 (4.0%) |  |
| Somewhat agree | 9 (12.2%) | 22 (9.9%) | 26 (7.5%) | 22 (6.5%) | 31 (10.2%) | 27 (9.1%) | 22 (10.3%) | 3 (6.0%) |  |
| Slightly disagree | 6 (8.1%) | 29 (13.0%) | 30 (8.6%) | 34 (10.1%) | 32 (10.5%) | 38 (12.8%) | 36 (16.8%) | 11 (22.0%) |  |
| Strongly disagree | 6 (8.1%) | 42 (18.8%) | 59 (17.0%) | 57 (17.0%) | 63 (20.7%) | 76 (25.7%) | 67 (31.3%) | 11 (22.0%) |  |
| No difference between men and women | 31 (41.9%) | 117 (52.5%) | 223 (64.3%) | 208 (61.9%) | 163 (53.6%) | 144 (48.6%) | 84 (39.3%) | 22 (44.0%) |  |
| *% Total of agreement** | *41.9%* | *15.7%* | *10.1%* | *11.0%* | *15.2%* | *12.9%* | *12.7%* | *12.0%* |  |
| **Rugby is a sport for females** | | | | | | | | | |
| Completely agree | 0 (0.0%) | 0 (0.0%) | 0 (0.0%) | 0 (0.0%) | 0 (0.0%) | 1 (0.3%) | 1 (0.5%) | 2 (4.0%) | **< 0.001** |
| Strongly agree | 1 (1.4%) | 1 (0.4%) | 0 (0.0%) | 1 (0.3%) | 0 (0.0%) | 2 (0.7%) | 0 (0.0%) | 3 (6.0%) |  |
| Somewhat agree | 1 (1.4%) | 6 (2.7%) | 5 (1.4%) | 6 (1.8%) | 8 (2.6%) | 8 (2.7%) | 7 (3.3%) | 1 (2.0%) |  |
| Slightly disagree | 10 (13.5%) | 53 (23.8%) | 65 (18.7%) | 87 (25.9%) | 98 (32.2%) | 83 (28.0%) | 69 (32.2%) | 21 (42.0%) |  |
| Strongly disagree | 33 (44.6%) | 37 (16.6%) | 52 (15.0%) | 47 (14.0%) | 64 (21.1%) | 80 (27.0%) | 63 (29.4%) | 13 (26.0%) |  |
| No difference between men and women | 29 (39.2%) | 126 (56.5%) | 225 (64.8%) | 195 (58.0%) | 134 (44.1%) | 122 (41.2%) | 74 (34.6%) | 10 (20.0%) |  |
| *% Total of agreement** | *2.8%* | *3.1%* | *1.4%* | *2.1%* | *2.6%* | *3.7%* | *3.8%* | *12.0%* |  |
| **Basketball is a game for females** | | | | | | | | | |
| Completely agree | 1 (1.4%) | 0 (0.0%) | 1 (0.3%) | 0 (0.0%) | 1 (0.3%) | 1 (0.3%) | 1 (0.5%) | 0 (0.0%) | **< 0.001** |
| Strongly agree | 0 (0.0%) | 1 (0.4%) | 2 (0.6%) | 1 (0.3%) | 0 (0.0%) | 1 (0.3%) | 0 (0.0%) | 1 (2.0%) |  |
| Somewhat agree | 2 (2.7%) | 5 (2.2%) | 4 (1.2%) | 2 (0.6%) | 5 (1.6%) | 3 (1.0%) | 1 (0.5%) | 1 (2.0%) |  |
| Slightly disagree | 16 (21.6%) | 31 (13.9%) | 24 (6.9%) | 38 (11.3%) | 39 (12.8%) | 22 (7.4%) | 25 (11.7%) | 9 (18.0%) |  |
| Strongly disagree | 22 (29.7%) | 23 (10.3%) | 33 (9.5%) | 28 (8.3%) | 37 (12.2%) | 51 (17.2%) | 32 (15.0%) | 11 (22.0%) |  |
| No difference between men and women | 33 (44.6%) | 163 (73.1%) | 283 (81.6%) | 267 (79.5%) | 222 (73.0%) | 218 (73.6%) | 155 (72.4%) | 28 (56.0%) |  |
| *% Total of agreement** | *4.1%* | *2.6%* | *2.1%* | *0.9%* | *1.9%* | *1.6%* | *1.0%* | *4.0%* |  |

*Note: The total percentage of agreement was calculated by summing the percentages of responses “completely agree”, “strongly agree”, and “somewhat agree”. This value is reported only for descriptive purposes and was not considered for the statistical analysis.

**Table 6Sb:** Stratification analyses conducted between each question composing the latent construct “Sports” (total of 10 questions) and gender categories. Results containing p-values < 0.05 are highlighted in bold.

|  | **Female (N=1292)** | **Male (N=534)** | **Other/Do not want to answer (N=18)** | **p-value** |
| --- | --- | --- | --- | --- |
| **Tennis is a sport for males** | | | | |
| Completely agree | 0 (0.0%) | 1 (0.2%) | 0 (0.0%) | 0.114 |
| Strongly agree | 2 (0.2%) | 1 (0.2%) | 0 (0.0%) |  |
| Somewhat agree | 5 (0.4%) | 16 (3.0%) | 1 (5.6%) |  |
| Slightly disagree | 48 (3.7%) | 42 (7.9%) | 1 (5.6%) |  |
| Strongly disagree | 366 (28.3%) | 97 (18.2%) | 3 (16.7%) |  |
| No difference between men and women | 871 (67.4%) | 377 (70.6%) | 13 (72.2%) |  |
| *% Total of agreement** | *0.6%* | *3.4%* | *5.6%* |  |
| **Artistic gymnastics is for females** | | | | |
| Completely agree | 10 (0.8%) | 28 (5.2%) | 0 (0.0%) | **< 0.001** |
| Strongly agree | 15 (1.2%) | 33 (6.2%) | 1 (5.6%) |  |
| Somewhat agree | 140 (10.8%) | 120 (22.5%) | 1 (5.6%) |  |
| Slightly disagree | 156 (12.1%) | 62 (11.6%) | 1 (5.6%) |  |
| Strongly disagree | 235 (18.2%) | 43 (8.1%) | 3 (16.7%) |  |
| No difference between men and women | 736 (57.0%) | 248 (46.4%) | 12 (66.7%) |  |
| *% Total of agreement** | *12.8%* | *27.9%* | *11.2%* |  |
| **Skiing is a sport for males** | | | | |
| Completely agree | 0 (0.0%) | 2 (0.4%) | 0 (0.0%) | 0.509 |
| Strongly agree | 0 (0.0%) | 0 (0.0%) | 0 (0.0%) |  |
| Somewhat agree | 3 (0.2%) | 12 (2.2%) | 1 (5.6%) |  |
| Slightly disagree | 29 (2.2%) | 24 (4.5%) | 0 (0.0%) |  |
| Strongly disagree | 348 (26.9%) | 97 (18.2%) | 4 (22.2%) |  |
| No difference between men and women | 912 (70.6%) | 399 (74.7%) | 13 (72.2%) |  |
| *% Total of agreement** | *0.2%* | *2.6%* | *5.6%* |  |
| **Rugby is a sport for males** | | | | |
| Completely agree | 28 (2.2%) | 59 (11.0%) | 1 (5.6%) | **< 0.001** |
| Strongly agree | 59 (4.6%) | 59 (11.0%) | 0 (0.0%) |  |
| Somewhat agree | 332 (25.7%) | 122 (22.8%) | 0 (0.0%) |  |
| Slightly disagree | 208 (16.1%) | 67 (12.5%) | 2 (11.1%) |  |
| Strongly disagree | 190 (14.7%) | 41 (7.7%) | 4 (22.2%) |  |
| No difference between men and women | 475 (36.8%) | 186 (34.8%) | 11 (61.1%) |  |
| *% Total of agreement** | *32.5%* | *44.8%* | *5.6%* |  |
| **Cycling is a sport for males** | | | | |
| Completely agree | 4 (0.3%) | 7 (1.3%) | 0 (0.0%) | **0.013** |
| Strongly agree | 9 (0.7%) | 7 (1.3%) | 0 (0.0%) |  |
| Somewhat agree | 72 (5.6%) | 58 (10.9%) | 1 (5.6%) |  |
| Slightly disagree | 128 (9.9%) | 47 (8.8%) | 0 (0.0%) |  |
| Strongly disagree | 267 (20.7%) | 77 (14.4%) | 5 (27.8%) |  |
| No difference between men and women | 812 (62.8%) | 338 (63.3%) | 12 (66.7%) |  |
| *% Total of agreement** | *6.6%* | *13.5%* | *5.6%* |  |
| **Boxing is a sport for males** | | | | |
| Completely agree | 33 (2.6%) | 43 (8.1%) | 1 (5.6%) | **< 0.001** |
| Strongly agree | 58 (4.5%) | 37 (6.9%) | 0 (0.0%) |  |
| Somewhat agree | 248 (19.2%) | 113 (21.2%) | 1 (5.6%) |  |
| Slightly disagree | 164 (12.7%) | 50 (9.4%) | 1 (5.6%) |  |
| Strongly disagree | 187 (14.5%) | 46 (8.6%) | 3 (16.7%) |  |
| No difference between men and women | 602 (46.6%) | 245 (45.9%) | 12 (66.7%) |  |
| *% Total of agreement** | *26.3%* | *36.2%* | *11.2%* |  |
| **Karate is a sport for males** | | | | |
| Completely agree | 6 (0.5%) | 13 (2.4%) | 0 (0.0%) | **< 0.001** |
| Strongly agree | 7 (0.5%) | 10 (1.9%) | 0 (0.0%) |  |
| Somewhat agree | 24 (1.9%) | 31 (5.8%) | 0 (0.0%) |  |
| Slightly disagree | 71 (5.5%) | 43 (8.1%) | 0 (0.0%) |  |
| Strongly disagree | 301 (23.3%) | 92 (17.2%) | 4 (22.2%) |  |
| No difference between men and women | 883 (68.3%) | 345 (64.6%) | 14 (77.8%) |  |
| *% Total of agreement** | *2.9%* | *10.1%* | *0.0%* |  |
| **Football is a sport for males** | | | | |
| Completely agree | 15 (1.2%) | 28 (5.2%) | 0 (0.0%) | **< 0.001** |
| Strongly agree | 19 (1.5%) | 30 (5.6%) | 1 (5.6%) |  |
| Somewhat agree | 83 (6.4%) | 79 (14.8%) | 0 (0.0%) |  |
| Slightly disagree | 155 (12.0%) | 60 (11.2%) | 1 (5.6%) |  |
| Strongly disagree | 311 (24.1%) | 65 (12.2%) | 5 (27.8%) |  |
| No difference between men and women | 709 (54.9%) | 272 (50.9%) | 11 (61.1%) |  |
| *% Total of agreement** | *9.1%* | *25.6%* | *5.6%* |  |
| **Rugby is a sport for females** | | | | |
| Completely agree | 1 (0.1%) | 3 (0.6%) | 0 (0.0%) | **0.028** |
| Strongly agree | 5 (0.4%) | 3 (0.6%) | 0 (0.0%) |  |
| Somewhat agree | 34 (2.6%) | 8 (1.5%) | 0 (0.0%) |  |
| Slightly disagree | 348 (26.9%) | 137 (25.7%) | 1 (5.6%) |  |
| Strongly disagree | 235 (18.2%) | 150 (28.1%) | 4 (22.2%) |  |
| No difference between men and women | 669 (51.8%) | 233 (43.6%) | 13 (72.2%) |  |
| *% Total of agreement** | *3.1%* | *2.7%* | *0.0%* |  |
| **Basketball is a game for females** | | | | |
| Completely agree | 3 (0.2%) | 2 (0.4%) | 0 (0.0%) | **< 0.001** |
| Strongly agree | 4 (0.3%) | 2 (0.4%) | 0 (0.0%) |  |
| Somewhat agree | 18 (1.4%) | 5 (0.9%) | 0 (0.0%) |  |
| Slightly disagree | 106 (8.2%) | 97 (18.2%) | 1 (5.6%) |  |
| Strongly disagree | 157 (12.2%) | 77 (14.4%) | 3 (16.7%) |  |
| No difference between men and women | 1004 (77.7%) | 351 (65.7%) | 14 (77.8%) |  |
| *% Total of agreement** | *1.9%* | *1.7%* | *0.0%* |  |

*Note: The total percentage of agreement was calculated by summing the percentages of responses “completely agree”, “strongly agree”, and “somewhat agree”. This value is reported only for descriptive purposes and was not considered for the statistical analysis.

**Table 6Sc:** Stratification analyses conducted between each question composing the latent construct “Sports” (total of 10 questions) and degree categories. Results containing p-values < 0.05 are highlighted in bold.

|  | **Elementary/Secondary school degrees (N=122)** | **High school degree (N=662)** | **University degree (N=737)** | **Post-University degree (N=323)** | **p-value** |
| --- | --- | --- | --- | --- | --- |
| **Tennis is a sport for males** | | | | | |
| Completely agree | 0 (0.0%) | 1 (0.2%) | 0 (0.0%) | 0 (0.0%) | **< 0.001** |
| Strongly agree | 0 (0.0%) | 1 (0.2%) | 1 (0.1%) | 1 (0.3%) |  |
| Somewhat agree | 9 (7.4%) | 8 (1.2%) | 3 (0.4%) | 2 (0.6%) |  |
| Slightly disagree | 10 (8.2%) | 32 (4.8%) | 26 (3.5%) | 23 (7.1%) |  |
| Strongly disagree | 26 (21.3%) | 185 (27.9%) | 188 (25.5%) | 67 (20.7%) |  |
| No difference between men and women | 77 (63.1%) | 435 (65.7%) | 519 (70.4%) | 230 (71.2%) |  |
| *% Total of agreement** | *7.4%* | *1.6%* | *0.5%* | *0.9%* |  |
| **Artistic gymnastics is for females** | | | | | |
| Completely agree | 12 (9.8%) | 14 (2.1%) | 3 (0.4%) | 9 (2.8%) | **< 0.001** |
| Strongly agree | 11 (9.0%) | 16 (2.4%) | 13 (1.8%) | 9 (2.8%) |  |
| Somewhat agree | 18 (14.8%) | 102 (15.4%) | 104 (14.1%) | 37 (11.5%) |  |
| Slightly disagree | 9 (7.4%) | 76 (11.5%) | 95 (12.9%) | 39 (12.1%) |  |
| Strongly disagree | 13 (10.7%) | 102 (15.4%) | 119 (16.1%) | 47 (14.6%) |  |
| No difference between men and women | 59 (48.4%) | 352 (53.2%) | 403 (54.7%) | 182 (56.3%) |  |
| *% Total of agreement** | *33.6%* | *19.9%* | *16.3%* | *17.1%* |  |
| **Skiing is a sport for males** | | | | | |
| Completely agree | 1 (0.8%) | 1 (0.2%) | 0 (0.0%) | 0 (0.0%) | **0.009** |
| Strongly agree | 0 (0.0%) | 0 (0.0%) | 0 (0.0%) | 0 (0.0%) |  |
| Somewhat agree | 6 (4.9%) | 5 (0.8%) | 1 (0.1%) | 4 (1.2%) |  |
| Slightly disagree | 6 (4.9%) | 14 (2.1%) | 21 (2.8%) | 12 (3.7%) |  |
| Strongly disagree | 27 (22.1%) | 175 (26.4%) | 183 (24.8%) | 64 (19.8%) |  |
| No difference between men and women | 82 (67.2%) | 467 (70.5%) | 532 (72.2%) | 243 (75.2%) |  |
| *% Total of agreement** | *5.7%* | *1.0%* | *0.1%* | *1.2%* |  |
| **Rugby is a sport for males** | | | | | |
| Completely agree | 26 (21.3%) | 32 (4.8%) | 15 (2.0%) | 15 (4.6%) | **< 0.001** |
| Strongly agree | 12 (9.8%) | 54 (8.2%) | 38 (5.2%) | 14 (4.3%) |  |
| Somewhat agree | 21 (17.2%) | 157 (23.7%) | 199 (27.0%) | 77 (23.8%) |  |
| Slightly disagree | 10 (8.2%) | 97 (14.7%) | 120 (16.3%) | 50 (15.5%) |  |
| Strongly disagree | 16 (13.1%) | 82 (12.4%) | 92 (12.5%) | 45 (13.9%) |  |
| No difference between men and women | 37 (30.3%) | 240 (36.3%) | 273 (37.0%) | 122 (37.8%) |  |
| *% Total of agreement** | *48.3%* | *36.7%* | *34.2%* | *32.7%* |  |
| **Cycling is a sport for males** | | | | | |
| Completely agree | 3 (2.5%) | 2 (0.3%) | 3 (0.4%) | 3 (0.9%) | **0.035** |
| Strongly agree | 4 (3.3%) | 7 (1.1%) | 4 (0.5%) | 1 (0.3%) |  |
| Somewhat agree | 14 (11.5%) | 48 (7.3%) | 46 (6.2%) | 23 (7.1%) |  |
| Slightly disagree | 11 (9.0%) | 53 (8.0%) | 85 (11.5%) | 26 (8.0%) |  |
| Strongly disagree | 17 (13.9%) | 140 (21.1%) | 134 (18.2%) | 58 (18.0%) |  |
| No difference between men and women | 73 (59.8%) | 412 (62.2%) | 465 (63.1%) | 212 (65.6%) |  |
| *% Total of agreement** | *17.3%* | *8.7%* | *7.1%* | *8.3%* |  |
| **Boxing is a sport for males** | | | | | |
| Completely agree | 19 (15.6%) | 25 (3.8%) | 20 (2.7%) | 13 (4.0%) | **0.018** |
| Strongly agree | 9 (7.4%) | 39 (5.9%) | 37 (5.0%) | 10 (3.1%) |  |
| Somewhat agree | 19 (15.6%) | 126 (19.0%) | 157 (21.3%) | 60 (18.6%) |  |
| Slightly disagree | 8 (6.6%) | 65 (9.8%) | 94 (12.8%) | 48 (14.9%) |  |
| Strongly disagree | 14 (11.5%) | 96 (14.5%) | 87 (11.8%) | 39 (12.1%) |  |
| No difference between men and women | 53 (43.4%) | 311 (47.0%) | 342 (46.4%) | 153 (47.4%) |  |
| *% Total of agreement** | *38.6%* | *28.7%* | *29.0%* | *25.7%* |  |
| **Karate is a sport for males** | | | | | |
| Completely agree | 9 (7.4%) | 5 (0.8%) | 2 (0.3%) | 3 (0.9%) | **< 0.001** |
| Strongly agree | 5 (4.1%) | 9 (1.4%) | 2 (0.3%) | 1 (0.3%) |  |
| Somewhat agree | 4 (3.3%) | 21 (3.2%) | 22 (3.0%) | 8 (2.5%) |  |
| Slightly disagree | 8 (6.6%) | 41 (6.2%) | 44 (6.0%) | 21 (6.5%) |  |
| Strongly disagree | 17 (13.9%) | 156 (23.6%) | 164 (22.3%) | 60 (18.6%) |  |
| No difference between men and women | 79 (64.8%) | 430 (65.0%) | 503 (68.2%) | 230 (71.2%) |  |
| *% Total of agreement** | *14.8%* | *5.4%* | *3.6%* | *3.7%* |  |
| **Football is a sport for males** | | | | | |
| Completely agree | 12 (9.8%) | 21 (3.2%) | 5 (0.7%) | 5 (1.5%) | **< 0.001** |
| Strongly agree | 10 (8.2%) | 19 (2.9%) | 15 (2.0%) | 6 (1.9%) |  |
| Somewhat agree | 15 (12.3%) | 68 (10.3%) | 60 (8.1%) | 19 (5.9%) |  |
| Slightly disagree | 10 (8.2%) | 70 (10.6%) | 87 (11.8%) | 49 (15.2%) |  |
| Strongly disagree | 15 (12.3%) | 147 (22.2%) | 161 (21.8%) | 58 (18.0%) |  |
| No difference between men and women | 60 (49.2%) | 337 (50.9%) | 409 (55.5%) | 186 (57.6%) |  |
| *% Total of agreement** | *30.3%* | *16.4%* | *10.8%* | *9.3%* |  |
| **Rugby is a sport for females** | | | | | |
| Completely agree | 0 (0.0%) | 3 (0.5%) | 1 (0.1%) | 0 (0.0%) | 0.756 |
| Strongly agree | 1 (0.8%) | 2 (0.3%) | 4 (0.5%) | 1 (0.3%) |  |
| Somewhat agree | 3 (2.5%) | 15 (2.3%) | 18 (2.4%) | 6 (1.9%) |  |
| Slightly disagree | 19 (15.6%) | 167 (25.2%) | 211 (28.6%) | 89 (27.6%) |  |
| Strongly disagree | 41 (33.6%) | 156 (23.6%) | 128 (17.4%) | 64 (19.8%) |  |
| No difference between men and women | 58 (47.5%) | 319 (48.2%) | 375 (50.9%) | 163 (50.5%) |  |
| *% Total of agreement** | *3.3%* | *3.1%* | *3.0%* | *2.2%* |  |
| **Basketball is a game for females** | | | | | |
| Completely agree | 1 (0.8%) | 0 (0.0%) | 2 (0.3%) | 2 (0.6%) | **0.002** |
| Strongly agree | 0 (0.0%) | 2 (0.3%) | 4 (0.5%) | 0 (0.0%) |  |
| Somewhat agree | 2 (1.6%) | 9 (1.4%) | 10 (1.4%) | 2 (0.6%) |  |
| Slightly disagree | 20 (16.4%) | 84 (12.7%) | 64 (8.7%) | 36 (11.1%) |  |
| Strongly disagree | 26 (21.3%) | 96 (14.5%) | 85 (11.5%) | 30 (9.3%) |  |
| No difference between men and women | 73 (59.8%) | 471 (71.1%) | 572 (77.6%) | 253 (78.3%) |  |
| *% Total of agreement** | *2.4%* | *1.7%* | *2.2%* | *1.2%* |  |

*Note: The total percentage of agreement was calculated by summing the percentages of responses “completely agree”, “strongly agree”, and “somewhat agree”. This value is reported only for descriptive purposes and was not considered for the statistical analysis.

**Table 7S:** Summary of the results of the principal component analysis performed on each latent construct, divided by all participants, and stratified by females and males. For each component, the cumulative variance, the item and its weight on the loadings is computed. Comp: Component; Var: Variance.

|  | **Home and family activities** | | | | | | |
| --- | --- | --- | --- | --- | --- | --- | --- |
|  |  | **Comp.1** | **Comp.2** | **Comp.3** | **Comp.4** | **Comp.5** | **Comp.6** |
| **All** | **Item** | Men drive better | Men drive better | Sewing is for men | Women must take care of the children |  |  |
|  | **Cumulative Var** | 0.47 | 0.60 | 0.68 | 0.73 |  |  |
|  | **Weight on the loadings** | 0.44 | 0.85 | 0.65 | -0.61 |  |  |
| **Females** | **Item** | Ironing is for women | Men drive better | Sewing is for men | Ironing is for women | Women must take care of the children |  |
|  | **Cumulative Var** | 0.40 | 0.52 | 0.61 | 0.69 | 0.76 |  |
|  | **Weight on the loadings** | 0.45 | 0.71 | 0.69 | -0.49 | 0.64 |  |
| **Males** | **Item** | Ironing is for women  Men drive better | Men drive better | Ironing is for women | Women must take care of the children |  |  |
|  | **Cumulative Var** | 0.54 | 0.67 | 0.73 | 0.78 |  |  |
|  | **Weight on the loadings** | 0.41 | 0.88 | 0.68 | -0.83 |  |  |
|  | **Games** | | | | | | |
|  |  | **Comp.1** | **Comp.2** | **Comp.3** | **Comp.4** | **Comp.5** | **Comp.6** |
| **All** | **Item** | Playing with dolls is for girls | Videogames are for males | The tricycle is for boys |  |  |  |
|  | **Cumulative Var** | 0.52 | 0.67 | 0.80 |  |  |  |
|  | **Weight on the loadings** | 0.72 | 0.85 | -0.46 |  |  |  |
| **Females** | **Item** | Playing with dolls is for girls | Videogames are for males | Playing with dolls is for girls |  |  |  |
|  | **Cumulative Var** | 0.48 | 0.66 | 0.78 |  |  |  |
|  | **Weight on the loadings** | 0.67 | 0.87 | 0.56 |  |  |  |
| **Males** | **Item** | Playing with dolls is for girls | Videogames are for males | Videogames are for males |  |  |  |
|  | **Cumulative Var** | 0.54 | 0.69 | 0.81 |  |  |  |
|  | **Weight on the loadings** | 0.72 | 0.66 | 0.58 |  |  |  |
|  | **Moral judgments** | | | | | | |
|  |  | **Comp.1** | **Comp.2** | **Comp.3** | **Comp.4** | **Comp.5** | **Comp.6** |
| **All** | **Item** | Men. more than women. can lose patience | Female infidelity is more serious than male infidelity |  |  |  |  |
|  | **Cumulative Var** | 0.66 | 0.89 |  |  |  |  |
|  | **Weight on the loadings** | 0.73 | 0.74 |  |  |  |  |
| **Females** | **Item** | Men. more than women. can lose patience | Female infidelity is more serious than male infidelity |  |  |  |  |
|  | **Cumulative Var** | 0.58 | 0.87 |  |  |  |  |
|  | **Weight on the loadings** | 0.76 | 0.76 |  |  |  |  |
| **Males** | **Item** | Men. more than women. can lose patience | Female infidelity is more serious than male infidelity |  |  |  |  |
|  | **Cumulative Var** | 0.73 | 0.92 |  |  |  |  |
|  | **Weight on the loadings** | 0.72 | 0.74 |  |  |  |  |
|  | **Personality traits** | | | | | | |
|  |  | **Comp.1** | **Comp.2** | **Comp.3** | **Comp.4** | **Comp.5** | **Comp.6** |
| **All** | **Item** | Women are more sensitive than men  Women are more determined than men  Women are more cunning than men | Women are more sensitive than men | Women are braver than men | For men. more than for women. it is important to success in job | For men. more than for women. it is important to success in job | Women are more cunning than men |
|  | **Cumulative Var** | 0.40 | 0.49 | 0.57 | 0.63 | 0.68 | 0.73 |
|  | **Weight on the loadings** | 0.33 | -0.52 | 0.78 | 0.77 | -0.47 | 0.71 |
| **Females** | **Item** | Women are braver than men | Women are more sensitive than men | Women are braver than men | For men. more than for women. it is important to success in job | Men are more aggressive than women | Women are more sensitive than men |
|  | **Cumulative Var** | 0.40 | 0.49 | 0.57 | 0.63 | 0.68 | 0.74 |
|  | **Weight on the loadings** | 0.37 | -0.52 | 0.71 | -0.85 | -0.50 | 0.48 |
| **Males** | **Item** | Women are more cunning than men | Women are more sensitive than men | Women are braver than men | For men. more than for women. it is important to success in job | Women are more cunning than men | Men are more aggressive |
|  | **Cumulative Var** | 0.40 | 0.49 | 0.57 | 0.63 | 0.68 | 0.74 |
|  | **Weight on the loadings** | 0.34 | -0.54 | 0.52 | 0.59 | -0.72 | 0.63 |
|  | **Profession/Jobs** | | | | | | |
|  |  | **Comp. 1** | **Comp.2** | **Comp.3** | **Comp.4** | **Comp.5** | **Comp.6** |
| **All** | **Item** | Babysitter is for women | Military profession is for men | Military profession is for men | Working in a butcher shop is for men |  |  |
|  | **Cumulative Var** | 0.48 | 0.57 | 0.66 | 0.74 |  |  |
|  | **Weight on the loadings** | 0.49 | 0.65 | -0.53 | -0.81 |  |  |
| **Females** | **Item** | Babysitter is for women | Military profession is for men | Babysitter is for women | Working in a butcher shop is for men | Being a bus driver is for women | Nursing profession is for women |
|  | **Cumulative Var** | 0.44 | 0.54 | 0.64 | 0.73 | 0.79 | 0.84 |
|  | **Weight on the loadings** | 0.52 | -0.80 | -0.74 | -0.73 | -0.67 | -0.67 |
| **Males** | **Item** | Military profession is for men  Babysitter is for women | Babysitter is for women | Military profession is for men | Working in a butcher shop is for men | Being a pilot is a job for men |  |
|  | **Cumulative Var** | 0.52 | 0.61 | 0.69 | 0.76 | 0.81 |  |
|  | **Weight on the loadings** | 0.44 | -0.76 | -0.74 | -0.81 | -0.83 |  |
|  | **Sports** | | | | | | |
|  |  | **Comp.1** | **Comp.2** | **Comp.3** | **Comp.4** | **Comp.5** | **Comp.6** |
| **All** | **Item** | Rugby is for men  Boxing is for men | Rugby is for men | Artistic gymnastics is for women |  |  |  |
|  | **Cumulative Var** | 0.59 | 0.68 | 0.76 |  |  |  |
|  | **Weight on the loadings** | 0.50 | -0.53 | -0.84 |  |  |  |
| **Females** | **Item** | Rugby is for men | Rugby is for men | Artistic gymnastics is for women | Boxing is for men |  |  |
|  | **Cumulative Var** | 0.57 | 0.67 | 0.75 | 0.81 |  |  |
|  | **Weight on the loadings** | 0.53 | -0.50 | -0.83 | -0.64 |  |  |
| **Males** | **Item** | Rugby is for men  Boxing is for men | Rugby is for men | Artistic gymnastics is for women |  |  |  |
|  | **Cumulative Var** | 0.57 | 0.67 | 0.75 |  |  |  |
|  | **Weight on the loadings** | 0.47 | -0.57 | -0.79 |  |  |  |
